# Early vertebrate chromosome duplications and the evolution of the neuropeptide Y receptor gene regions

**DOI:** 10.1186/1471-2148-8-184

**Published:** 2008-06-25

**Authors:** Tomas A Larsson, Frida Olsson, Gorel Sundstrom, Lars-Gustav Lundin, Sydney Brenner, Byrappa Venkatesh, Dan Larhammar

**Affiliations:** 1 Department of Neuroscience, Uppsala University, Box 593, 75124 Uppsala, Sweden; 2 Institute of Molecular and Cell Biology, Agency for Science, Technology and Research, Biopolis, 138673, Singapore

## Abstract

**Background:**

One of the many gene families that expanded in early vertebrate evolution is the neuropeptide (NPY) receptor family of G-protein coupled receptors. Earlier work by our lab suggested that several of the NPY receptor genes found in extant vertebrates resulted from two genome duplications before the origin of jawed vertebrates (gnathostomes) and one additional genome duplication in the actinopterygian lineage, based on their location on chromosomes sharing several gene families. In this study we have investigated, in five vertebrate genomes, 45 gene families with members close to the NPY receptor genes in the compact genomes of the teleost fishes *Tetraodon nigroviridis *and *Takifugu rubripes*. These correspond to *Homo sapiens *chromosomes 4, 5, 8 and 10.

**Results:**

Chromosome regions with conserved synteny were identified and confirmed by phylogenetic analyses in *H. sapiens, M. musculus, D. rerio, T. rubripes *and *T. nigroviridis*. 26 gene families, including the NPY receptor genes, (plus 3 described recently by other labs) showed a tree topology consistent with duplications in early vertebrate evolution and in the actinopterygian lineage, thereby supporting expansion through block duplications. Eight gene families had complications that precluded analysis (such as short sequence length or variable number of repeated domains) and another eight families did not support block duplications (because the paralogs in these families seem to have originated in another time window than the proposed genome duplication events). RT-PCR carried out with several tissues in *T. rubripes *revealed that all five NPY receptors were expressed in the brain and subtypes Y2, Y4 and Y8 were also expressed in peripheral organs.

**Conclusion:**

We conclude that the phylogenetic analyses and chromosomal locations of these gene families support duplications of large blocks of genes or even entire chromosomes. Thus, these results are consistent with two early vertebrate tetraploidizations forming a paralogon comprising human chromosomes 4, 5, 8 and 10 and one teleost tetraploidization. The combination of positional and phylogenetic data further strengthens the identification of orthologs and paralogs in the NPY receptor family.

## Background

The evolutionary relationships of the NPY-receptor family receptors in vertebrates have been difficult to resolve due to uneven evolutionary rates and because some subtypes are missing in some classes of vertebrates. By using information on chromosomal location, initially in pig and human [1,2], we suggested that chromosome duplications could account for the origin of several new family members. However, the relationships of the bony fish receptors called Y8a and Y8b, discovered in zebrafish and initially named Yc and Yb [3,4], respectively, remained speculative [5] because they seemed to lack mammalian and bird orthologs.

Gene duplication by tetraploidization in the chordate lineage was proposed by Susumu Ohno in 1970 [6], based upon chromosome numbers and DNA content in different lineages. The first gene mapping data supporting a tetraploidization scenario emerged in 1987 when two human Hox clusters were mapped to human chromosomes Hsa7 and Hsa17 (Hsa for Homo sapiens) which resembled one another also with regard to other gene families [7]. Lundin described similarities in the other two Hox-bearing chromosomes, thereby identifying a quartet of related regions [8,9]. The Hox chromosomes are now known to have involved duplication of more than 50 gene families [10-12].

In addition to the Hox-chromosome similarities, Lundin also reported resemblance within three other groups of human chromosomes. One group consisted of Hsa4 and Hsa5 [9], later found to contain NPY receptor genes [1] and extended to include Hsa8 and Hsa10 [13,14]. Relationships between other chromosomes have been described by several authors, see for instance [11,15-21]. Such groups of related, or paralogous, chromosome regions are called paralogons [22]. In tetrapod vertebrates, the paralogons are often comprised of quartets, consistent with a double tetraploidization scenario, called 2R for two rounds of genome doubling, before the origin of gnathostomes (jawed vertebrates) [23] although it is difficult to ascertain that the complete genome was quadrupled. Indeed, some regions do not have any paralogous counterparts [24]. More recently, a third tetraploidization has been identified in euteleost fish [25-28]. Several additional tetraploidizations have been described in specific lineages of for example fish and amphibians [29-32].

The sizes of the quadrupled paralogous gene regions have been difficult to determine because of numerous chromosomal rearrangements during the approximately 500 Myr since the tetraploidizations. Several vertebrate genome projects have recently been reported or are in progress, but due to incomplete assembly of the sequences into contigs or scaffolds, let alone chromosomes, these cannot always be used to analyze conserved synteny or paralogous gene regions. Another complicating factor has been the uneven divergence rates in some of the daughter genes after the duplications [10,33-35] thereby aggravating the dating of the duplications. Indeed, inconsistent gene family phylogenies have been used as an argument against the tetraploidization hypothesis [36], although this can be seen as a natural consequence of uneven selection pressures or uneven re-diploidization rates after the two tetraploidizations, particularly as these may have taken place very close in time [10,35,37,38].

Our laboratory has previously reported that the genes encoding NPY (neuropeptide Y)-family receptors, which belong to the superfamily of rhodopsin-like G-protein-coupled receptors (GPCRs), are located in the paralogon comprised of the human chromosomes Hsa4, 5 and 10 [1]. The fourth original chromosome member was shown to be partially represented by Hsa8 and Hsa2 [1,14], although neither of the latter two chromosomes harbors NPY receptor genes. Our observation was based on a comparison of the human, mouse and pig chromosome regions [1] and has subsequently been supported by our analysis of the chicken NPY receptor genes [39]. However, neither the organization of NPY receptor genes in the recently reported euteleost fish genomes nor the extent of the chromosome regions comprising this paralogon or the phylogenetic relationships of the gene families involved, have been analyzed in detail.

We report here studies of 45 gene families whose members are located on Hsa 4, 5, 8/2/7 and 10. We have investigated conservation of synteny in human, mouse and three euteleost fishes, starting with the compact genomes of *Tetraodon. nigroviridis *and *Takifugu. rubripes*, and performed phylogenetic analyses of these gene families. This approach has been named "transitive homology" [40,41] and allows for the identification of paralogous chromosomal segments despite the frequent loss of genes or rearrangement of gene order along chromosomes. The combined results of phylogenetic analyses and chromosomal locations reveal duplications of large chromosomal regions and are consistent with two basal vertebrate tetraploidizations, as well as the third tetraploidization in euteleosts. This analysis helps to clarify the evolutionary history of the chromosomal regions harboring the vertebrate NPY receptor genes and also further facilitates orthology/paralogy assignments of genes in the NPY receptor gene family.

## Results

### NPY receptors in *Takifugu rubripes *and *Tetraodon nigroviridis*

**Figure 1:**
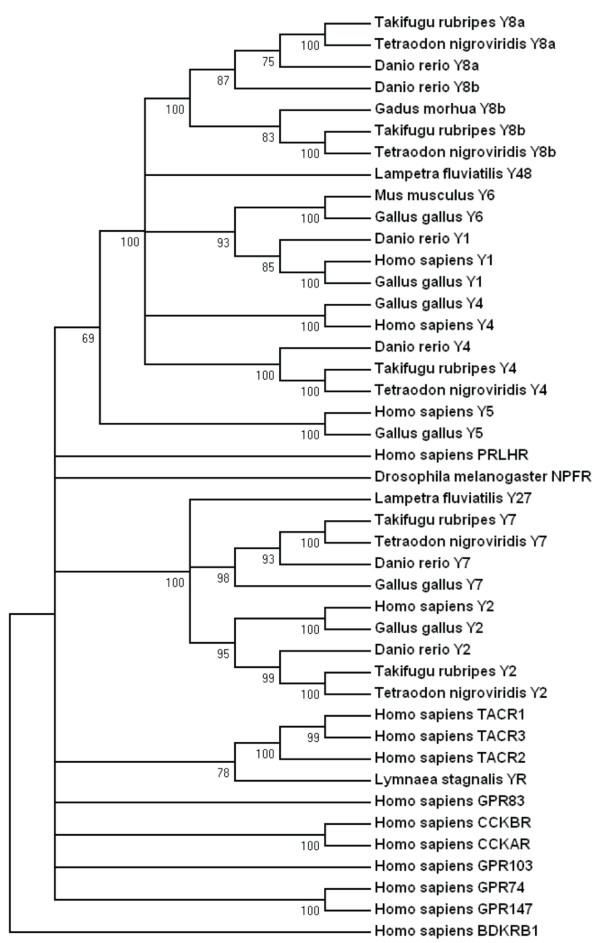
**Phylogenetic neighbor-joining tree of NPY receptors and closely related GPCRs**. Numbers below branches show percent bootstrap support for each node. Nodes with values below 50 have been collapsed. Human bradykinin receptor B1 was used to root the tree.

Both the *T. nigroviridis *genome and the *T. rubripes *genome was confirmed to harbor five NPY receptor genes previously found in *D. rerio *namely Y4 (Ya) [42], Y8a (Yc) [3] and Y8b (Yb) [4] belonging to the Y1 subfamily of receptors and Y2 and Y7 belonging to the Y2 subfamily. In *D. rerio *a Y1 receptor has been found recently that is not represented in the pufferfish genome databases [43]. So far no Y5 gene has been found in any of these three well-studied teleost fish species. The phylogenetic tree used to assign subfamily membership is shown in Fig. 1.

### RT-PCR and annotation of pufferfish genes

**Figure 2:**
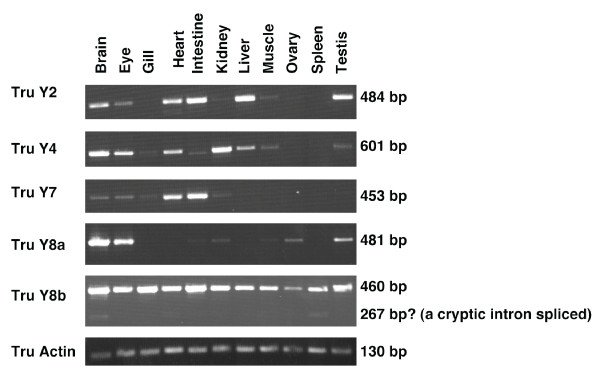
**RT-PCR in *Takifugu rubripes***. Agarose gel showing the expression of the five *Takifugu rubripes *receptor genes in eleven different tissues. Actin was used as control to verify quality and content of samples.

The RT-PCR carried out on eleven tissues in *T. rubripes *(Fig. 2) showed expression in brain and eye for all five receptors. Y8b was expressed in all eleven tissues while the other four receptors showed a more narrow expression pattern. Y8b also showed two distinct bands in some tissues because of alternative splicing. Interestingly, Y7 showed expression in several tissues in contrast to the very narrow expression observed for chicken Y7 [39].

**Figure 3:**
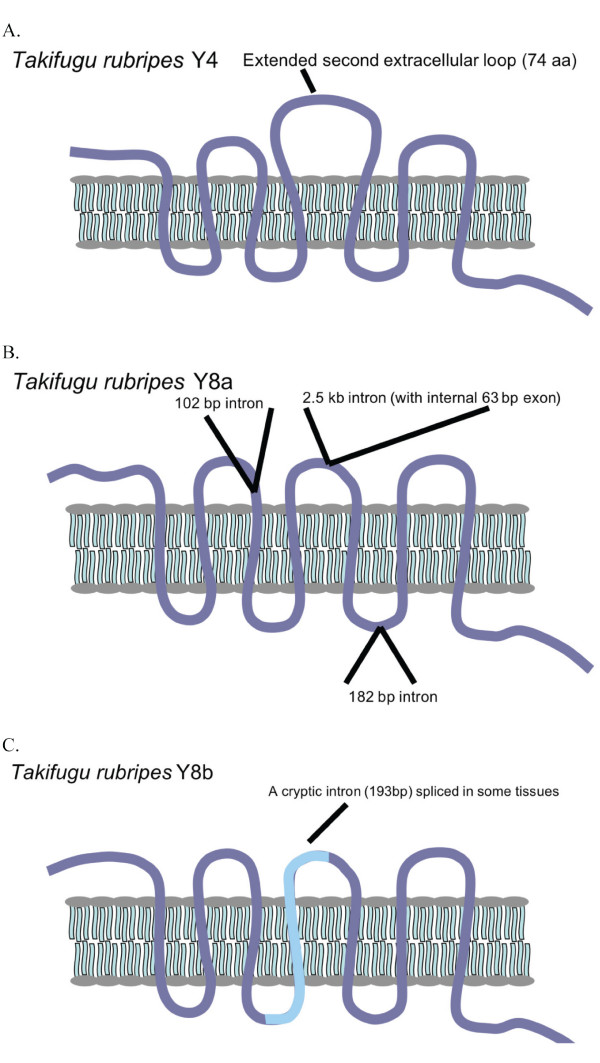
**Schematic picture of *Takifugu rubripes *NPY receptors**. Picture depicting the Y4 (panel A), Y8a (panel B) and Y8b (panel C) receptors in *Takifugu rubripes*, indicating the extended second extracellular loop in Y4, the three extra introns present in the Y8a receptor gene and the alternative splicing of Y8b.

The sequenced RT-PCR products revealed that the Y4 sequence contained an extended second extracellular loop in *T. rubripes *(Fig. 3A). This extension was also seen in the *T. nigroviridis *Y4 sequence obtained from the database. In addition, Y8a in *T. rubripes *has three novel introns. One intron comprising 102 bp is located in the end of the first extracellular loop. A second intron of 2.5 kb is present in the region encoding the middle of extracellular loop 2, and this intron contains an additional short (63 bp) exon that accounts for the extension of extracellular loop 2. A third 182 bp intron has been inserted in the region encoding intracellular loop 3 (Fig. 3B). Comparison with the *T. nigroviridis *Y8a sequence showed it to have the same overall organization (the zebrafish Y8a gene lacks introns in the coding region). The total length of Y8a is 450 aa in *T. nigroviridis *and 452 aa in *T. rubripes*. The *T. rubripes *Y8b gene lacks introns in the coding region apart from a cryptic intron spliced in some tissues (see Fig. 2 and 3C). The *T. rubripes *receptor sequences have been deposited to GenBank with the following accession numbers: [GenBank:EU104001 (Y2), GenBank:EU104002 (Y4), GenBank:EU104003 (Y7), GenBank:EU104004 (Y8a) and GenBank:EU104005 (Y8b)].

### Conserved synteny and paralogous regions

**Figure 4:**
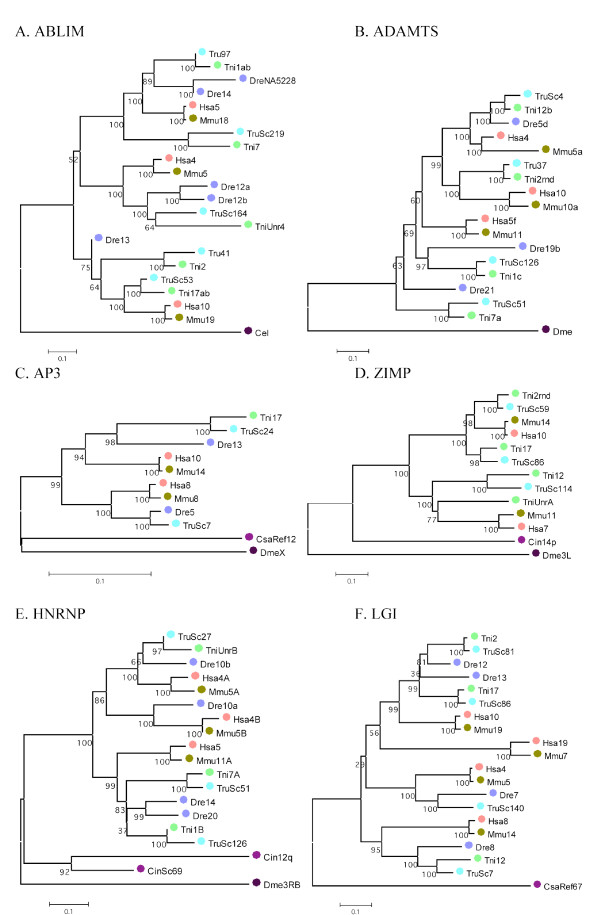
**A-F – Phylogenetic analysis of neighboring gene families**. Examples of the phylogenetic relationship for six neighboring gene families A (ABLIM), B (ADAMTS), C (AP3), D (ZIMP), E (HNRNP) and F (LGI). The trees were constructed using the neighbor-joining method as implemented in MEGA 3.1 with pair-wise deletion of gaps and poisson-corrected distances [117] All datasets were bootstrapped 1000 times (percent bootstrap support are indicated below each node). Species abbreviations are: Dme (*Drosophila melanogaster*), Cel (*Caenorhabditis elegans*), Cin (*Ciona intestinalis*), Csa (*Ciona savignyi*), Tru (*Takifugu rubripes*), Tni (*Tetraodon nigroviridis*), Dre (*Danio rerio*), Mmu (*Mus musculus*), Hsa (*Homo sapiens*). Numbers refers to chromosome number or scaffold number (ScXX), letters after chromosomal/scaffold number was arbitrarily assigned to family members located on the same chromosome to tell them apart. The scale below every tree indicates numbers of substitutions/site.

**Figure 5:**
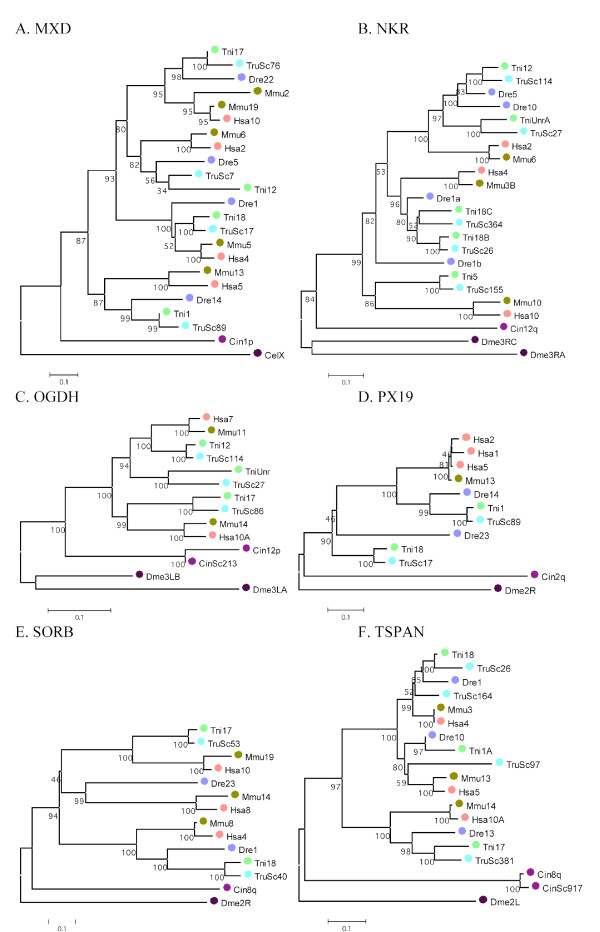
**A-F – Phylogenetic analysis of neighboring gene families**. A-F. Examples of the phylogenetic relationship for six additional neighboring gene families A (MAX), B (NKR), C (OGDH), D (PX19), E (SORB) and F (TSPAN). Trees were constructed and visualized in the same way as in Fig. 4.

**Figure 6:**
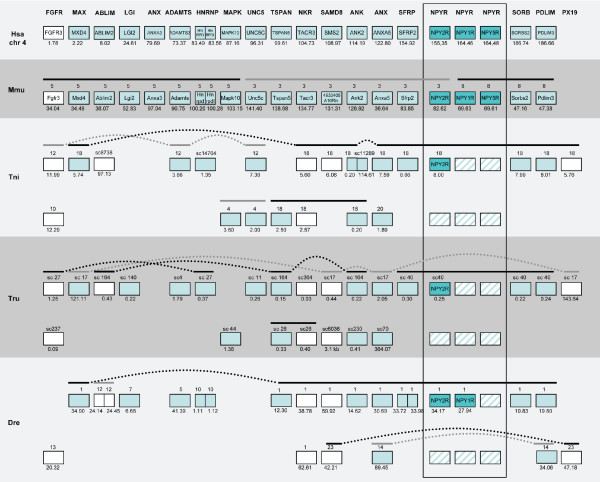
**Conserved synteny among vertebrate species compared to human chromosome 4**. Conservation of synteny for genes investigated in this study residing on human chromosome 4 compared to mouse, *T. rubripes*, *T. nigroviridis *and *D. rerio*. Numbers above boxes denotes chromosome numbers or scaffold names. Position of the genes (Mega base pairs) is given below each box. Genes are ordered according to their positions on the human chromosome. Genes linked in the other species are indicated with lines above boxes. Species name abbreviations are: Hsa (*Homo sapiens*), Mmu (*Mus musculus*), Tni (*Tetraodon nigroviridis*), Tru (*Takifugu rubripes*) and Dre (*Danio rerio*). The NPY receptors are indicated in darker color while loss of NPY receptor genes are indicated with striped boxes. White boxes indicate genes where the phylogenetic analysis is inconclusive but where position indicates orthology.

**Figure 7:**
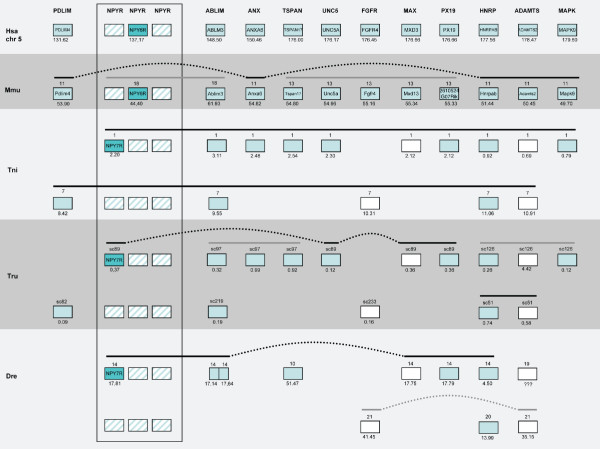
**Conserved synteny among vertebrate species compared to human chromosome 5**. Conservation of synteny for genes investigated in this study residing on human chromosome 5 compared to mouse, *T. rubripes*, *T. nigroviridis *and *D. rerio*. Chromosomes, scaffolds, gene positions, gene order and species abbreviations are given in the same way as in Fig. 6.

**Figure 8:**
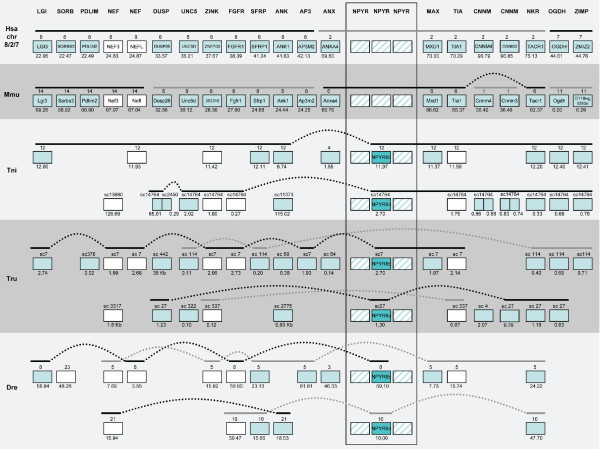
**Conserved synteny among vertebrate species compared to human chromosomes 8, 2 and 7**. Conservation of synteny for genes investigated in this study residing on human chromosome 8, 2 and 7 compared to mouse, *T. rubripes*, *T. nigroviridis *and *D. rerio*. Chromosomes, scaffolds, gene positions, gene order and species abbreviations are given in the same way as in Fig. 6.

A total of 35 gene families were found with members in three or four of the regions harboring the *T. nigroviridis *NPY receptor genes. In addition to these, another 9 families were included due to linkage to the NPY receptors in *T. rubripes*. Phylogenetic analysis of these 45 families (including the NPY receptor family) confirmed 26 to be compatible with an expansion in vertebrate evolution before the origin of gnathostomes. Neighbor-joining trees are shown in Fig. 4A–F and 5A–F for twelve of these families. The full set of NJ and ML-trees are available as Additional files 1 and 2, respectively. The total number of genes located in the investigated regions linked to the NPY receptor genes in *T. nigroviridis *was 556 (Y4), 310 (Y8a), 375 (Y8b), 487 (Y2) and 370 (Y7) according to the 34.1d version of the Ensembl database. This number of genes (2098) represents approximately 7.5% of the total gene number in the *T. nigroviridis *genome (total gene number estimated to be 28005 in this release of the database). The corresponding human orthologs situated on chromosomes 4, 5, 8/2/7 and 10 are dispersed over a large portion of these chromosomes. The synteny group associated with *T. nigroviridis *Y8a/Y8b seems to have been broken up in the human genome because some families are located on chromosome 8 and a few are on chromosomes 2 and 7. In addition to the 35 gene families with at least three members on the *T. nigroviridis *chromosomes, 127 gene families were identified that are represented on two of the chromosomes [see Additional file 3]. The conservation of synteny for the four chromosomes in human and mouse and eight chromosomes in the three fish species is illustrated in Fig. 6, 7, 8, 9. A schematic view of the evolution of 16 of the investigated gene families and the NPY receptor family is shown in Fig. 10.

**Figure 9:**
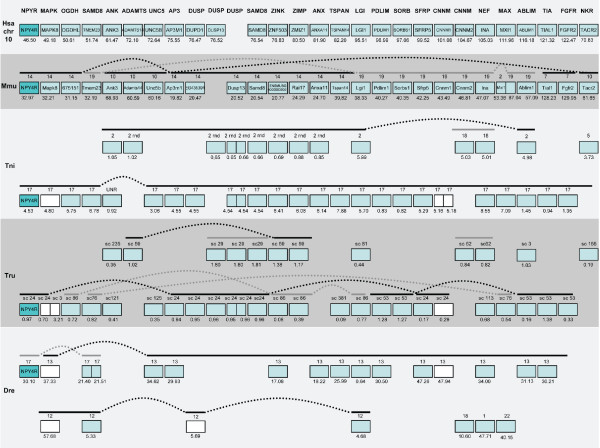
**Conserved synteny among vertebrate species compared to human chromosome 10**. Conservation of synteny for genes investigated in this study residing on human chromosome 10 compared to mouse, *T. rubripes*, *T. nigroviridis *and *D. rerio*. Chromosomes, scaffolds, gene positions, gene order and species abbreviations are given in the same way as in Fig. 6.

**Figure 10:**
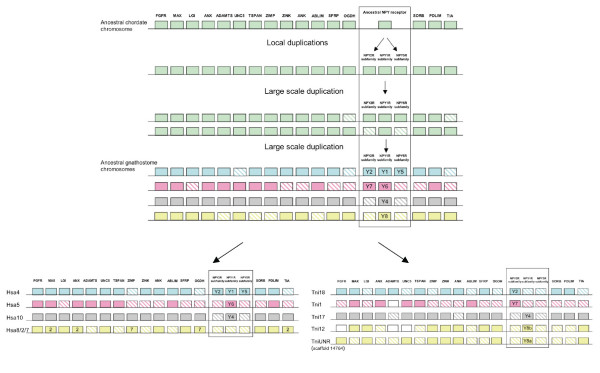
**Proposed evolutionary scenario for the NPY receptor gene chromosomes**. Schematic picture showing the proposed evolutionary history for 16 gene families located on the same chromosomes as the NPY receptor genes in *T. nigroviridis *and human. Note that position of genes is shuffled to simplify the picture. Striped boxes indicate gene losses. Position for genes on Hsa2 and Hsa7 is indicated in the boxes.

### Statistical testing of paralogous regions

The statistical test of the investigated paralogous regions based on a binomial test as used previously by Vienne et al. [14] shows that their positions are different from a random distribution (P << 0.05) in both the genomes of *T. nigroviridis *(98 paralogs in total with 26 outside the investigated regions) and human (74 paralogs with 5 paralogs outside of the investigated regions). These results are in agreement with previous results investigating other genes present on Hsa 4, 5, 8 and 10 [14].

### Summary of gene families analyzed in this study

Descriptions of the 25 subfamilies linked to the NPY receptor genes investigated in detail in this study are given below, i.e., the families that have members on 3 or 4 of the paralogous chromosomal regions in *T. nigroviridis *and families known to be linked to the NPY receptor genes in the *T. rubripes *genome. Three families that also support block or chromosome duplications were recently analyzed by other groups and are therefore not described here, namely two subfamilies of adrenergic receptors and the ADAM family of metzincins [44,45].

Eight families with members on these chromosomes showed a tree topology seemingly inconsistent with expansion in early vertebrate evolution i.e. with several outgroup sequences dispersed among the vertebrate sequences, suggesting earlier origin of these paralogs. In addition to these families, eight families had to be left out of the analysis because of short sequence length, high conservation and therefore uninformative alignments or difficulties to generate reasonable alignments due to varying numbers of repeated domains. For a complete list of all families in the regions investigated (including the 127 families with only 2 members in *T. nigroviridis *and families studied due to members being present on the NPY receptor scaffolds in *T. rubripes*) see Additional file 3. For several of the analyzed families the number of fish sequences predicted to be part of the family according to the Ensembl database is higher than the number in our final trees. This is because we had to exclude some family members that lack one or several domains in order to obtain alignments of sufficient quality for phylogenetic analysis. Thus the number of fish paralogs in some of the families analyzed is a conservative representation of the actual number. We believe that this will be improved with more refined versions of the fish genomes (especially the *Danio rerio *genome) as well as comparison with additional genomes not available at the onset of this study.

#### ABLIM

The actin-binding LIM family has four members in the human genome. They are characterized by the presence of 4 LIM-domains and one villin domain. These proteins have been implicated in modulation of cell shape and cell differentiation through interaction with the actin cytoskeleton [46,47]. The orthologous protein found in *C. elegans *has also been shown to interact with actin and has been shown to mediate axon guidance [48]. Expression of the different members of this protein family has been observed in distinct areas of the nervous system for example retina (ABLIM-1) [46], caudate/putamen (ABLIM-2), olfactory bulb (ABLIM-3), hippocampus (ABLIM-2 and ABLIM-3) and cerebellum (ABLIM-2 and ABLIM-3) [47]. The topology of the tree is in agreement with expansion of this gene family in the vertebrate lineage.

#### ADAMTS

The ADAMTS protein family (a disintegrin-like and metalloprotease protein with trombospondin motifs) includes proteins with a metalloprotease domain, disintegrin and spacer domains and a number of trombospondin repeats. Members of this protein family have been implicated in several diseases [49]. The human genome has so far been shown to contain more than 20 members of this gene family. The initial phylogenetic analysis identified a subfamily with members on human chromosomes 4 (ADAMTS3), 5 (ADAMTS2) and 10 (ADAMTS14), also identified in other studies [49]. The final analysis of this subfamily is in agreement with an expansion of this gene family in the vertebrate lineage before the divergence of actinopterygians and sarcopterygians.

#### Ankyrin (ANK)

The ankyrin gene family is one of the families earlier found to be part of a paralogon [1,14] with members on human chromosomes 4, 8 and 10. We recovered a similar topology to previous studies with high bootstrap support. In addition the tree showed a topology consistent with extra duplication events in the teleost lineage, i.e. two fish genes corresponding to one tetrapod gene. The 33 aa ankyrin repeat is one of the most common protein domains in the pfam database.

#### Annexin (ANX)

A core domain consisting of four repeated units, each about 70 amino acids long, characterizes the annexin family. Each repeat unit contains 5 alpha helices that usually contain a "type 2" motif for binding of Ca^2+ ^ions [50]. The phylogenetic analysis recovered a topology expected by expansion in the early vertebrate lineage and also evidence for local duplications before the origin of vertebrates as well as additional fish specific and a few tetrapod specific duplications. In addition, the positions of the genes investigated in this study agree with block duplications of the chromosomal regions harboring these genes.

#### AP3

This family contains two members in the human genome (AP3M1 and AP3M2) on Hsa8 and Hsa10 consistent with early vertebrate block duplication. They are members of a larger family of proteins called adaptins that are important for vesicular transport common to all eukaryotes [51,52]. The mammalian AP-3 complex has been demonstrated to interact with clathrin and has been implicated in lysosomal membrane protein trafficking, sorting of melanosomal proteins and neurotransmitter/synaptic vesicle formation [51].

#### CNNM

The CNNM protein family (also referred to as ACDP) contains four members in both the human and the mouse genomes [53,54]. These genes share one highly conserved domain, the ACD domain, which is present in a large number of species. The functions of these proteins are largely unknown. Immunofluorescence studies showed all four members to be localized to the nucleus and it has been speculated that these genes are probably involved in cell cycle regulation due to their similarity to cyclins [53]. The topology of the tree indicates a local duplication before the split of actinopterygians and sarcopterygians followed by block duplications and also some additional local duplicates in the fish lineage.

#### DUSP

The dual specificity phosphatases (or MAP kinase phosphatases) are evolutionarily conserved enzymes that are important in the regulation of apoptosis, cell proliferation and cell differentiation. They exert their effects by dephosphorylating and thereby inactivating MAP kinases [55]. The recovered topology in this study is in agreement with an expansion of this gene family in the vertebrate lineage. However the relationship of the different paralogs was not similar to that proposed earlier [14] possibly due to inclusion of other species in this analysis. Based on our analysis it is unlikely that the mouse gene named Dusp13 is the ortholog of human DUSP13.

#### Fibroblast growth factor receptor (FGFR)

The fibroblast growth factor receptors comprise one of the families already described as being a part of this paralogon [1,14]. The topology of the phylogenetic tree is consistent with an expansion in the vertebrate lineage followed by further expansion in the teleost lineage. Interestingly, one of the families included in our initial list of gene families linked to the NPY receptor genes in *T. nigroviridis *is a subfamily of the fibroblast growth factors (FGFs) suggesting that both ligands and receptors were duplicated in the same time window. Hurst and Lercher observed that many ligand genes are linked to their receptor genes (including several of the FGFs and FGFRs) and that this could be due to block duplication that would keep the ratio of gene products at the same level as before duplication [56]. Our analysis of the FGF family was inconclusive in assigning orthology for the different members due to the rather short length of the sequences (~120aa). For detailed overviews of FGF and FGFR evolution with discussion about the role of gene and genome duplication see [57-59].

#### HNRNP

The HNRNP family is a family of proteins that contain two RBD or RRM domains. These proteins are known to interact with telomeric repeats d(TTAGGG) and to 3'splice sites r(UUAG/G) and have also been proposed to have a role in the regulation of mRNA stability. One of the human members, HNRPD has previously been mapped to Hsa 4q21 and been shown to have a highly conserved ortholog in the mouse genome [60]. Our present analysis recovered a subfamily with two members on Hsa4 and one member on Hsa5. The relationship of the fish sequences included was not resolved by this analysis, possibly due to loss of family members in human and mouse.

#### LGI

The gene *LGI1 *(leucine-rich gene – glioma inactivated) was discovered by positional cloning in 1998 and found to be mainly expressed in neural tissues, particularly in the brain. The gene is localized to human chromosome 10q24, a region found to be rearranged or deleted in several types of malignant brain tumors. Because of this *LGI1 *was proposed to be a tumor suppressor gene [61]. Three additional genes with similarity to *LGI1 *were found in the human genome and it was shown that these four genes constitute a subfamily of leucine rich repeat (LRR) genes [62]. The topology of the tree is consistent with duplications before the sarcopterygian-actinopterygian split, but one of the genes is located on Hsa19 rather than Hsa5.

#### MAPK

A subfamily of the large superfamily of mitogen-activated protein kinases (MAPK) was identified as being part of this paralogon. This subfamily contains the human members MAPK8, MAPK9 and MAPK10 (JNK1, JNK2 and JNK3). These proteins are involved in a wide variety of cellular processes like cell growth, proliferation, differentiation, immunity and development [63]. For a recent review of their role in vertebrate development see [64]. The gene duplications agree well with the block duplications prior to the sarcopterygian-actinopterygian divergence.

#### MXD

The MXD protein family has four members in the human genome. These proteins are characterized by a common basic-helix-loop-helix leucine zipper domain necessary for the formation of heterodimers with other proteins, such as Myc and Max [65]. The function of these proteins as regulators of Myc and Max activity with implications for tumour formation has recently been reviewed [66]. The topology of the phylogenetic tree and the positional information strongly supports the paralogy of the studied regions. It does not contain any extra fish specific duplicates.

#### NEF (Intermediate filaments/neurofilaments)

All intermediate filaments have a similar structural organization with a central alpha-helical rod domain that begins and ends with highly conserved aa-motifs necessary for correct assembly. Intermediate filament proteins have earlier been viewed as scaffolding structural proteins but more and more data suggest much more dynamic roles for these proteins [67]. The phylogeny of this protein family supports expansion in the vertebrate lineage. Interestingly, it seems like one subfamily in this gene family belongs to the HOX paralogon (with members on Hsa 2, 7, 12 and 17), which also contains the NPY peptide family. The tree also indicates a local duplication before the divergence of actinopterygians and sarcopterygians and additional fish specific duplications.

#### NKR

The tachykinin receptors are GPCRs that bind the amidated neuropeptides substance P, neurokinin A and neurokinin B as well as some other related peptides [68]. In vertebrates, three different tachykinin receptors have been described so far [68]. Our phylogenetic analysis of this receptor family shows a topology consistent with an expansion in the vertebrate lineage in agreement with the previous hypothesis [2], as well as an additional expansion consistent with block duplication in the teleost lineage.

#### OGDH

Oxoglutarate dehydrogenase (OGDH also designated E1k or lipoamide) makes up a part of the enzyme complex responsible for conversion of α-ketoglutarate to succinyl coenzyme A in the Krebs cycle [69]. The gene for OGDH and one related sequence (OGDHL) have previously been mapped to Hsa 7 and Hsa 10 respectively [70]. Both of the pufferfish species have an ortholog of OGDH and a presumed 3R co-ortholog (Fig. 8) and an OGDHL ortholog (Fig. 9). Oxoglutarate dehydrogenase has previously been shown to be involved in the production of reactive oxygen species in the brain of mice and thereby has been ascribed a role in neuronal cell death [71]. Apart from this, little is known about the function and evolution of this protein family.

#### PDLIM

The PDZ and LIM domain-containing family is a multi domain protein family characterized by the presence of one PDZ and one or several LIM domains. Both the ABLIM family described above and the PDLIM family described here belong to the same superfamily [72]. Our analysis uncovered a topology in accordance with expansion in the vertebrate lineage. This family contains a full quartet in the human genome as expected by 2R without loss of genes, although it does not display an (A,B)(C,D)-topology, presumably due to unequal evolutionary rates after duplication.

#### PX19

This family contains a conserved domain named MSF (after the protein MSF1 found in the yeast *Saccaromyces cerevisiae*). This family contains three closely related members of duplicated genes in the human lineage clearly duplicated after 2R but the two fish genes support the paralogon described here. The function of these proteins is unknown but they are thought to be involved in intra-mitochondrial protein sorting. One of the human genes is present on Hsa5 and earlier observed to be linked to one member of the MXD family (see above) [73].

#### SAMD8

The SAMD8 family (here named after one of its members) contains three different genes in the human genome: SAMD8 (referred to as sphingomyelin synthase-related protein 1 or sterile alpha motif domain-containing 8), TMEM23 or Mob (referred to as transmembrane protein 23, sphingomyelin synthase 1 or protein mob) and SMS2 (Sphingomyelin synthase 2). Proteins in this family all contain a SAM domain and 4–6 transmembrane domains and constitute a subset of a larger family of spingomyelin synthases [74]. Our analysis was indicative of an early local duplication event before the divergence between actinopterygians and sarcopterygians, with two genes still present on Hsa10 and one on Hsa4.

#### SFRP

The secreted frizzled related proteins (SFRP, also referred to as secreted apoptosis related proteins, SARPS) have five members in the human genome, three of which are located in the regions analyzed here. The SFRP family is characterized by a frizzled domain and a C-terminal netrin domain. Proteins of the SFRP family have been implicated in regulating Wnt-frizzled signalling either by interacting with Wnts or the frizzled receptors [75,76]. Our phylogenetic analysis defines these three genes as a subgroup that has expanded during the evolution of vertebrates. Rattner et al. described this family in the mouse and showed that the members of this family was linked to some of the genes investigated in this study [77]. Of the fishes included here, only *Danio rerio *is indicative of any extra duplication.

#### SORB

The SORB or vinexin family has three members in the human genome namely vinexin, CAP (c-Cbl associated protein)/ponsin and ArgBP2 (Arg-binding protein 2). Common for all these proteins is that they contain a sorbin homology domain (pfamID PF02208) and three SH3 domains [78]. Members of this family have been implicated in the regulation of cell adhesion, and cytoskeletal organization and also growth factor signalling by functioning as adaptor proteins, connecting various other proteins [78]. This family supports an expansion in the proposed time window but does not show any evidence for a fish-specific duplication.

#### TIA

The two RNA binding proteins TIA1 (RNA-binding protein TIA-1) [79] and TIAR (TIA-1-related protein) [80] are the sole human members of a protein family that has been implicated in induction of apoptosis in certain cell types. Although the gene family only contains two members in the genomes of human and mouse, the phylogenetic and positional information support the conserved synteny of these chromosomes in this study. In the fishes this family supports the block duplication hypothesis for the chromosomes harboring the NPY8a and NPY8b receptors despite the failure to determine clear orthology relationships.

#### TSPAN

Tetraspanins are a large family of membrane proteins with four transmembrane domains (hence the name). These proteins are present in a wide variety of organisms and it has been proposed that the tetraspanin-like proteins present in plants share a common origin with tetraspanins in animals [81]. Tetraspanins are generally expressed in all cell types and usually several types are co-expressed. Their functions include various types of cell-cell and matrix-cell interactions and they have been implied in forming membrane microdomain structures and thereby working as "molecular facilitators" [82] or "molecular organizers" [81]. A total of 33 tetraspanins has previously been reported in human and 47 in bony fishes [81]. We identified a subfamily comprised of several vertebrate sequences as well as invertebrate outgroup sequences that support the block duplication hypothesis.

#### UNC5

The transmembrane protein UNC-5 was first characterized in *C. elegans *and implicated in regulation of netrin signalling since mutation of unc-5 causes neural migration defects while ectopic expression of unc-5 causes netrin-dependent redirection of axon growth in some neurons. Four different paralogs of UNC-5-like proteins have been found in vertebrates. These proteins contain two extracellular immunoglobulin-like domains and two extracellular trombospondin type 1 (TSP_1) domains [83] and three intracellular domains (ZU-5, DB and DD) [84]. The neuron guidance functions of this receptor family in response to netrins and possible interaction partners is reviewed in [84]. The topology of the tree is compatible with the 2R hypothesis and also shows some fish-specific duplicates. The fact that one rarely sees the perfect (A,B)(C,D)-topology for quartets of genes could be explained by unequal evolutionary rates following duplication (see also PDLIM above). However, when phylogenies and positional information are taken together the block duplication hypothesis is supported.

#### ZIMP

The ZIMP family of proteins contains a conserved SP-Ring/Miz domain, which they share with other PIAS proteins (protein inhibitors of activated STATS). In human, this family contains two members on Hsa7 and Hsa10 [85]. Both these proteins have been shown to regulate androgen receptor activity [86,87]. Zimp10 has also been described to have a role in TGF-β/Smad signaling [88]. The phylogenetic analysis and position of the fish genes support the block duplication of the chromosomal segment harboring the Y8a and Y8b receptors.

#### ZINK

The ZINK family contains two proteins (zink finger protein 703 and zink finger protein 503) in the human genome. Not much is known about the functions of these proteins. They both contain a classical C2H2 zinc finger domain (pfam ID PF00096). The positional and phylogenetic information for these two genes are in agreement with block duplication before the actinopterygian-sarcopterygian split. The *T. nigroviridis *genome has two additional members resulting from the teleost fish tertaploidization on Tni2 and Tni Unr.

## Discussion

The aim of the present study was to analyze in detail the phylogeny of gene families neighboring the NPY receptor genes to see if their evolutionary history was consistent with block/chromosome duplications [1,2,5]. We approached this by analyzing gene families on either side of the NPY-receptor family genes in the *T. nigroviridis *and *T. rubripes *genomes. We also used the information from the analyses of neighboring gene families to assign orthology and paralogy of the NPY receptor genes. The present study identified the receptors Y2, Y4, Y7, Y8a and Y8b in the two pufferfish genomes (Fig. 1). The chromosomal locations of the Y8a and Y8b genes show that they reside on two related fish chromosomes that most likely arose in the teleost tetraploidization (3R). Furthermore, the gene neighbors on these chromosomes strongly suggest that the corresponding ancestral teleost chromosome belongs to a quartet of ancestral gnathostome chromosomes that most likely arose in the proposed basal vertebrate tetraploidizations (Fig. 6, 7, 8, 9). The zebrafish Y8a gene is located on chromosome 17 according to the database version used in this study but has previously been mapped to chromosome 10 [42] (see Fig. 8). Thus, we can confirm and extend the proposed gene duplication scheme [5] so that it accounts for all of the NPY-family receptors in mammalian and teleost genomes (Fig. 10): an ancestral local triplication was followed by the basal vertebrate tetraploidizations whereupon several genes were probably lost, resulting in an ancestral gnathostome repertoire of seven NPY receptor genes.

In the tetrapod lineage, Y7 and Y8 are present in the frog *Xenopus tropicalis *(unpublished data) and Y7 is still present in chicken [39] while both the Y7 and Y8 genes seem to have been lost in the lineage leading to mammals. In the actinopterygian lineage leading to euteleosts, one additional copy arose in the teleost 3R tetraploidization, while two genes seem to have been lost in euteleosts, Y5 and Y6. We recently discovered the Y1 gene in the zebrafish genome but it has so far not been found in the genomes of pufferfishes, medaka or stickleback and may have been lost. For a description of the NPY receptor repertoire in basal teleosts see Salaneck et al. [43]. Finally, also the prolactin releasing hormone receptor gene shares the same degree of identity with the NPY-family receptors as the Y1–Y2–Y5 subfamilies display to one another [89], but the preferred ligand is not NPY and therefore we have not included it in the duplication scheme.

Our characterization of the five *T. rubripes *genes revealed that both Y4 and Y8a have received insertions in the coding region after divergence from the lineage leading to zebrafish, as seen for the melanocortin receptor genes MC2R and MC5R in pufferfish [90], despite the intron and general genome compaction of these genomes [91]. In the case of Y4 the insertion extends extracellular loop 2 with 74 amino acids (Fig. 3A). The Y8a gene has undergone no less than three insertions of introns whose positions are projected onto the protein structure shown in Fig. 3B. The presence of the insertion in Y4 mRNA and the removal of the three Y8a introns were confirmed by RACE and PCR in *T. rubripes*. The largest of the Y8a introns is 2.5 kb and carries a small exon that extends extracellular loop 2 with 21 amino acids. In the Y8b gene one cryptic intron is spliced and this shortened splice variant is present in a minor proportion of the mRNAs and presumably leads to a nonfunctional partial receptor protein. This cryptic intron splice site is also present in the medaka gene found in the genome database. We suggest that the Y4 insertion was also probably a reinserted intron that subsequently lost its splice signals, but thanks to its small size and the maintained reading frame it could be tolerated as a protein expansion in extracellular loop 2. Functional expression of the receptors will be necessary to see if the extensions of loop 2 in Y4 and Y8a affect the ligand-binding properties of the receptors. Studies of the anatomical distribution of the mRNAs for the five NPY receptors in *T. rubripes*, as detected by RT-PCR, show that all five are present in the brain and eye. Interestingly, Y8a and Y8b differ greatly in their distribution in that Y8b is expressed in all organs investigated whereas Y8a shows the narrowest distribution of all receptors, although Y8a and Y8b are most closely related to each other having originated in 3R. Functional information is still missing for many fish NPY-family receptors. The pufferfish NPY system is more complicated than in most other vertebrates because there are four peptide ligands due to 3R duplicates of both NPY and its relative PYY [92].

Several authors have discussed what criteria one should have for identifying paralogous regions and to safely infer that block duplications or chromosome duplications have occurred [93-96]. We argue that as many species as possible, with divergencies close in time to the proposed chromosome duplication events, should be included in the analyses to be able to date duplications and to reveal fluctuations of evolutionary rates among gene duplicates and across lineages. This also helps identify translocations and inversions as well as lineage-specific duplications and deletions. Due to high frequency of chromosomal rearrangements, inter- as well as intra-chromosomal, the gene content of chromosomal blocks can be considered sufficient to identify duplications on these time scales (several hundred million years). Given the high rate of deletion after duplication we want to emphasize the importance of combining map-based and phylogenetic approaches in order to understand the evolution of genomic regions. In this way, gene families with only two members (as opposed to the expected 4 as predicted by 2R) still give important information.

It has been suggested that the observed patterns of paralogy within genomes, often interpreted to be the remnants of large-scale duplications, could be the result of convergent evolution [97]. We believe this alternative explanation to be unlikely not only because polyploidization has been shown to be common in many lineages but mainly because reconstructions of ancestral chromosomal regions based on comparisons of vertebrate genomes shows that these paralogous regions span large genomic regions in many species [21]. If many small independent events produced the observed patterns of chromosomal similarity, one would need to infer that these events occurred in a relatively short period of time before the vertebrate radiation, otherwise one has to invoke several independent but identical duplication and translocation events giving the same chromosomal organization in different lineages. To our knowledge, no mechanism has been described in metazoans that could support such a scenario.

Among the gene neighbors of the NPY receptor genes, we analyzed 44 gene families with members on the chromosomes bearing NPY-receptor genes. In fact, chromosomes orthologous to three of the four human chromosomes were all found to be present in duplicate in the fish genomes, consistent with the 3R teleost tetraploidization (see Fig. 7, 8 and 9). This pattern has been referred to as "doubly conserved synteny" [26]. The phylogenetic relationships of 25 of these gene families are consistent with concomitant block or chromosome duplications. Another three gene families included in the initial table [see Additional file 3], have earlier been proposed to be part of this paralogon and recently been studied in detail by others (two subfamilies of adrenergic receptors [44,98] and members of the large gene superfamily of metzincins other than the ADAMTS family included in our analysis [45]).

Eight gene families had to be excluded from analysis because they had too many family members to achieve a reasonably clear picture of their evolutionary relationships or problems in analyzing them due to too high sequence conservation to be informative or varying number of protein domains making it hard to obtain unequivocal alignments. More thorough analysis using diagnostic positions and comparisons of intron-exon structure could be used to clarify the relation of the different paralogs of these families. Thus, our study provides a minimal estimate of the number of gene families that expanded simultaneously with the NPY receptor genes.

Out of the 44 gene families a total of 26+3 support block/chromosome duplications whereas eight gene families are unsupportive of the block duplication scheme with invertebrate sequences located between the vertebrate paralogs. The reasons for this may be earlier expansions of these families, uneven evolutionary rates among the daughter genes, gene conversion between family members or simply translocation of distant family members to the chromosomes in this study.

We uncovered evidence for extra fish duplicates, in agreement with 3R, for 18 of these 26 gene families. However, because we were forced to exclude several gene family members as they lacked one or a few protein domains, this is certainly an underestimate of the true number (see above). It has been pointed out earlier that the number of genes retained after 3R is rather low as compared to after 1R and 2R [99]. One good example of this is the well studied Hox clusters that have lost many duplicates [100].

The fact that there are 127 gene families with members on two of the investigated *T. nigroviridis *chromosomes may further corroborate the conserved synteny between the chromosomal regions in this study, although they need to be analyzed phylogenetically as well. Among these 127 pairs of paralogs, all ten possible combinations of the five *T. nigroviridis *chromosomes are represented. The lack of sequence data from several important species is still a limiting factor in investigation of the early vertebrate chromosome duplications. In particular, sequences from the cephalochordate *Branchiostoma floridae *(first draft version of assembly recently released but with limited data on gene linkage) and representatives of the jawless and cartilaginous fishes will help to date the block/chromosome duplication events when more extensive gene contigs have been generated.

One interesting observation is that members of some of the gene families studied here have been reported to interact with each other in signaling networks or possess domains that commonly interact with each other. This opens for the possibility that blocks of genes that were duplicated simultaneously can co-evolve and therefore one might detect subfunctionalization of entire gene networks and not only of different paralogs within one family. Examples of such families according to the BioGRID database of protein interactions [101,102] are the MAPK and the DUSP families, the ABLIM and NEF families, the ANX and NEF families, the CNNM and HNRNP families, the PDLIM and NEF families, the SAMD8 and ADAMTS families and the SAMD8 and the HNRNP families. However, these families contain many members and are known to have a diverse set of interaction partners and therefore functional experiments are needed to specifically test the interaction of the paralogs analyzed in this study. This observation also leads to speculation about differential retention of gene family members after duplication because of their different functions or involvement in signaling networks. A higher retention rate after duplication of genes and specialization of different paralogs that are tied up in signaling networks has been proposed earlier [103]. This also has been shown for genetic networks in yeast (*Saccharomyces cerevisiae*) duplicated by tetraploidization around 100 million years ago [104] as well as for duplicates in *Arabidopsis thaliana *produced by the most recent polyploidizations in this species that took place 20 to 60 million years ago [105,106]. These processes are also linked to the predictions regarding gene fate after duplication deduced from models of subfunctionalization [107-110] stating that paralogs could be fixed in the genome by a partitioning of ancestral functions, something that would lead to a higher retention of gene duplicates that could subsequently evolve new functions [111,112].

In earlier analyses of these paralogous regions using several other gene families, the order of duplication events has been inferred based on the phylogenetic trees giving highest support for the (Hsa8, Hsa10), (Hsa4, Hsa5) topology [14]. In our dataset this is also the most frequent relationship observed for the human chromosomes, possibly reflecting the order of duplication events.

## Conclusion

In summary, we have characterized the NPY receptor repertoire in the two pufferfishes *T. rubripes *and *T. nigroviridis *and compared the chromosomal regions where the receptor genes are located in one additional fish and two mammalian species. The conserved synteny shows that many of the gene families were located together in the same chromosome regions of the common ancestor of gnathostomes more than 400 Myr ago [113] (for a summary see Fig. 10). Our results are in line with the tetraploidizations in early vertebrate evolution as well as an additional tetraploidization in teleosts. Although gene losses are frequent after duplication it is possible to infer paralogy and orthology in this way by analyzing both phylogentic and positional information simultaneously. This "transitive homology" approach [40,41] in combination with dating of duplication events in relation to speciation events is in our opinion, as shown by the present study, a more reliable way to unravel the evolutionary history of gene families in cases where phylogenetic analyses alone are not fully informative.

## Methods

### Identification and analysis of NPY receptor genes in *Tetraodon nigroviridis *and *Takifugu rubripes*

BLAST searches were carried out on the Ensembl database version 35.1d [114] using human and zebrafish NPY receptor sequences in order to identify all NPY receptor sequences in the genomes of the two pufferfishes *T. rubripes *and *T. nigroviridis*. The sequences found were aligned with previously known NPY receptor sequences and closely related peptide binding receptors using the Windows version of ClustalX 1.81 [115,116]. The alignment was manually edited to remove poorly aligned residues. Thereafter an initial phylogenetic analysis was performed using the neighbor-joining method in MEGA3.1 [117] with standard settings in order to assign which sub-families of receptors that were represented in the pufferfish genomes. In addition to the NJ-tree a quartet-puzzling tree was constructed with Treepuzzle 5.2 [118] using the same alignment. This tree was made with the following settings: 9 categories of sites (8 gamma + 1 invariant, parameters estimated from the dataset using the "exact-slow" option) with 10000 puzzling steps using the JTT substitution matrix (tree not shown).

The NJ-tree was bootstrapped 1000 times. Several closely related human sequences were included in the analyses (see Fig. 1). Human bradykinin B1 receptor was used to root the tree and nodes with bootstrap support values below 50% were collapsed (see Fig. 1).

### RT-PCR in *Takifugu rubripes*

Total RNA was isolated from eleven *T. rubripes *tissues using TRIzol reagent (Invitrogen, USA) according to manufacturer's protocol. Purified total RNA was reverse transcribed and single-strand 5'RACE-ready cDNA was prepared using SMART RACE cDNA Amplification Kit (Clontech, USA). Primers used for the receptors as well as an internal actin control are listed in Table 1. The PCR was carried out according to the following protocol: a denaturation step at 95°C for 2 min, 35 cycles of 95°C 30 sec, 55°C for 1 min, 72°C for 1 min followed by a final elongation step at 72°C for 5 min. Identity of representative RT-PCR products were confirmed by sequencing on an Applied Biosystems 3700 DNA Analyzer using dye-terminator chemistry.

**Table 1:** Primers used for RT-PCR on eleven *Takifugu rubripes *tissues.

Gene	Forward primer	Reverse primer	Size of fragment
Y2	5'-ACTCTCATCTACACGCTGTACGG-3'	5'-CATCAGCAACCATCTTGGTGGTCTT-3'	484
Y4	5'-TCATGGACCATTGGGTGTTTGGCTC-3'	5'-GCCATGCGCTGGCACTCTGGA-3'	601
Y7	5'-CACTCTGGTTTACACTCTGCTGGAT-3'	5'-TCGTTGCGAGTGGATGGGCTGA-3'	453
Y8a	5'-ACCGCTGGGTGCTGGGAGAG-3'	5'-CGTCGTTTCAGGCGAAGGAAGAT-3'	481
Y8b	5'-GACCGCTGGATCCTGGGCGAT-3'	5'-TGTTTCTCTTCTGGGCGCCGT-3'	460 (267)
Actin	5'-AACTGGGAYGACATGGAGAA-3'	5'-TTGAAGGTCTCAAACATGAT-3'	130

### Phylogenetic analysis of neighboring genes

Starting from the confirmed receptor genes in the *T. nigroviridis *genome, all Ensembl Gene IDs for genes positioned four megabases on each side of the receptor genes were downloaded and saved in an Excel file. In addition to Ensembl Gene IDs the file contained information on Ensembl Family ID and family description as well as information about chromosomal position and human orthologs. This list was sorted based on Ensembl Family ID and all multiple entries for the same gene due to multiple transcripts were removed. The gene families containing members close to three or more of the *T. nigroviridis *NPY receptor genes were used for phylogenetic analysis. In addition to gene families found in this way, several families with members on the NPY receptor gene harboring scaffolds in *T. rubripes *were included [see Additional file 3]. All amino acid sequences of members included in the Ensembl families were downloaded from *T. nigroviridis*, *T. rubripes*, *Danio rerio, Homo sapiens *and *Mus musculus*. Invertebrate sequences representing at least one outgroup species were included in order to root the trees. For the main part of the trees both *Drosophila melanogaster *and *Ciona intestinalis *was used as outgroups. In cases where no clear orthologs could be found in these two species we used sequences from other invertebrate genomes available in the Ensembl database [114] (see figure legends and additional files for complete description of outgroups). Sequences for each family were aligned using MEGA 3.1 with default settings. All alignments were manually inspected and short and poorly aligned sequences were removed. Sequence alignments were further adjusted with the aid of the pfam database [119] for prediction of protein domains. Relevant literature describing the families was also used to find description of the domains. Thereafter the cut sequences were realigned and neighbor-joining trees was constructed using MEGA 3.1 for each family with pairwise deletion of gaps, 1000 bootstrap replicates and poisson-corrected distances. Alignments for the 26 families analyzed in detail are available [see Additional file 4].

Initial phylogenetic trees were inspected for topologies consistent with an expansion of the family in vertebrate evolution i.e. the outgroup sequence/sequences rooting several of the sequences residing on the particular chromosomes under study (for example Hsa 4, 5, 8 and 10). In cases where a large number of sequences were included in the Ensembl family, the initial phylogenetic tree was used to find relevant sub families represented by multiple vertebrate sequences and at least one outgroup sequence. The identified sub families were realigned and subjected to neighbor-joining analysis as described above. In addition to gene families represented on three or more of the *T. nigroviridis *chromosomes all families with members on two of the chromosomes were saved in a table [see Additional file 3]. Phylogenetic trees with a topology consistent with expansion in vertebrate evolution before the origin of gnathostomes using the NJ-method were examined using the Quartet-puzzling method in the Windows version of Treepuzzle 5.2 [118] to further support the result. The settings used for each analysis was the same as mentioned above for the NPY receptor family but with varying number of puzzling steps (1000–25000) depending on the size of the family.

### Statistical testing of paralogous regions

The position of paralogs were statistically tested for random distribution in both the human and *T. nigroviridis *genomes using the binomial test as described earlier by Vienne et al. [14]. Genes belonging to the same family residing close to each other on the same chromosome that grouped together in the phylogenetic trees were counted as one member because they are most probably the result of recent lineage specific local duplications and thereby not of interest in our analysis. The chromosomes tested for random distribution were the ones shown by phylogenetic analysis to contain several paralogs (i.e human chromosomes 4, 5, 8/2/7 and 10). For details on statistical analysis: [see Additional file 5].

## Authors' contributions

TAL performed the phylogenetic and chromosome analyses and drafted and coordinated the manuscript, FO performed many of the initial analyses, GS participated in the phylogenetic and chromosome analyses and contributed to the manuscript. L–GL spawned the concept of duplication of these chromosomal regions and contributed conceptually. SB participated in the design of experiments and contributed conceptually. BV performed the experimental work in *T. rubripes *and wrote part of the manuscript. DL conceived and initiated the study, and participated in its design and coordination and helped to draft the manuscript. All authors read and approved the final manuscript.

## Supplementary Material

Additional file 1 Neighbor-joining trees for the 26 gene families analyzed in detail. Click here for file

Additional file 2 Quartet puzzling trees for the 26 gene families analyzed in detail. Click here for file

Additional file 3 List of gene families linked to the NPY receptor genes in *Tetraodon nigroviridis*. Click here for file

Additional file 4 Compressed archive of alignment files for all analyzed families in fasta format. Click here for file

Additional file 5 Summary of statistical testing of paralogous regions. Click here for file
