# Supplementary material for: Early vertebrate chromosome duplications and the evolution of the neuropeptide Y receptor gene regions
Source: BMC Evol Biol. 2008 Jun 25;8:184. doi: 10.1186/1471-2148-8-184 (PMC2453138; doi:10.1186/1471-2148-8-184)
Supplement: Additional file 1 — Neighbor-joining trees for the 26 gene families analyzed in detail. [file 1471-2148-8-184-S1.pdf]

ABLIM NJ

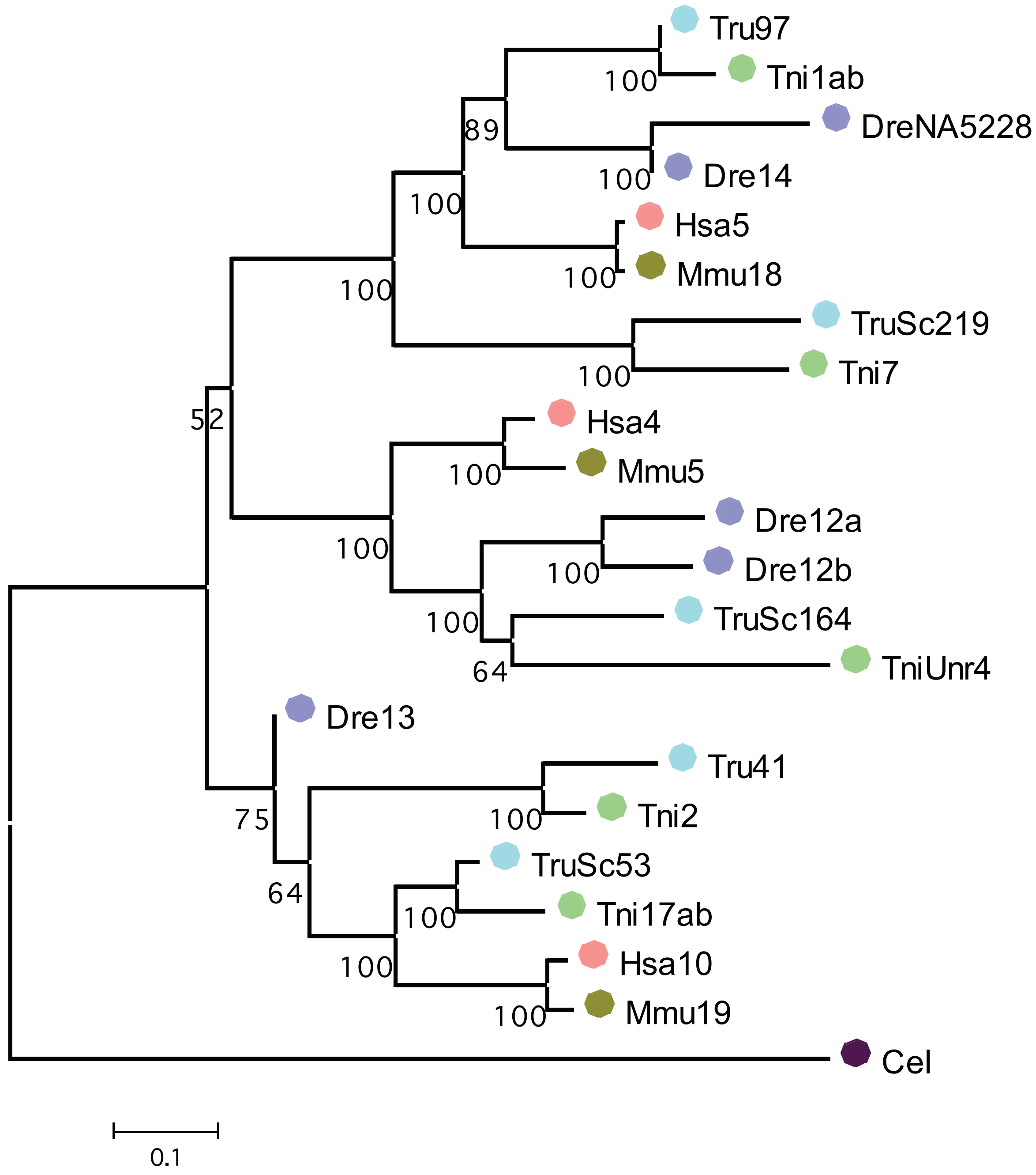
Tru97
100
Tni1ab
89
DreNA5228
100
Dre14
100
Hsa5
100
Mmu18
100
TruSc219
100
Tni7
52
Hsa4
100
Mmu5
100
Dre12a
100
Dre12b
100
TruSc164
64
TniUnr4
Dre13
Tru41
75
100
Tni2
64
TruSc53
100
Tni17ab
100
Hsa10
100
Mmu19
Cel
0.1

ADAMTS NJ

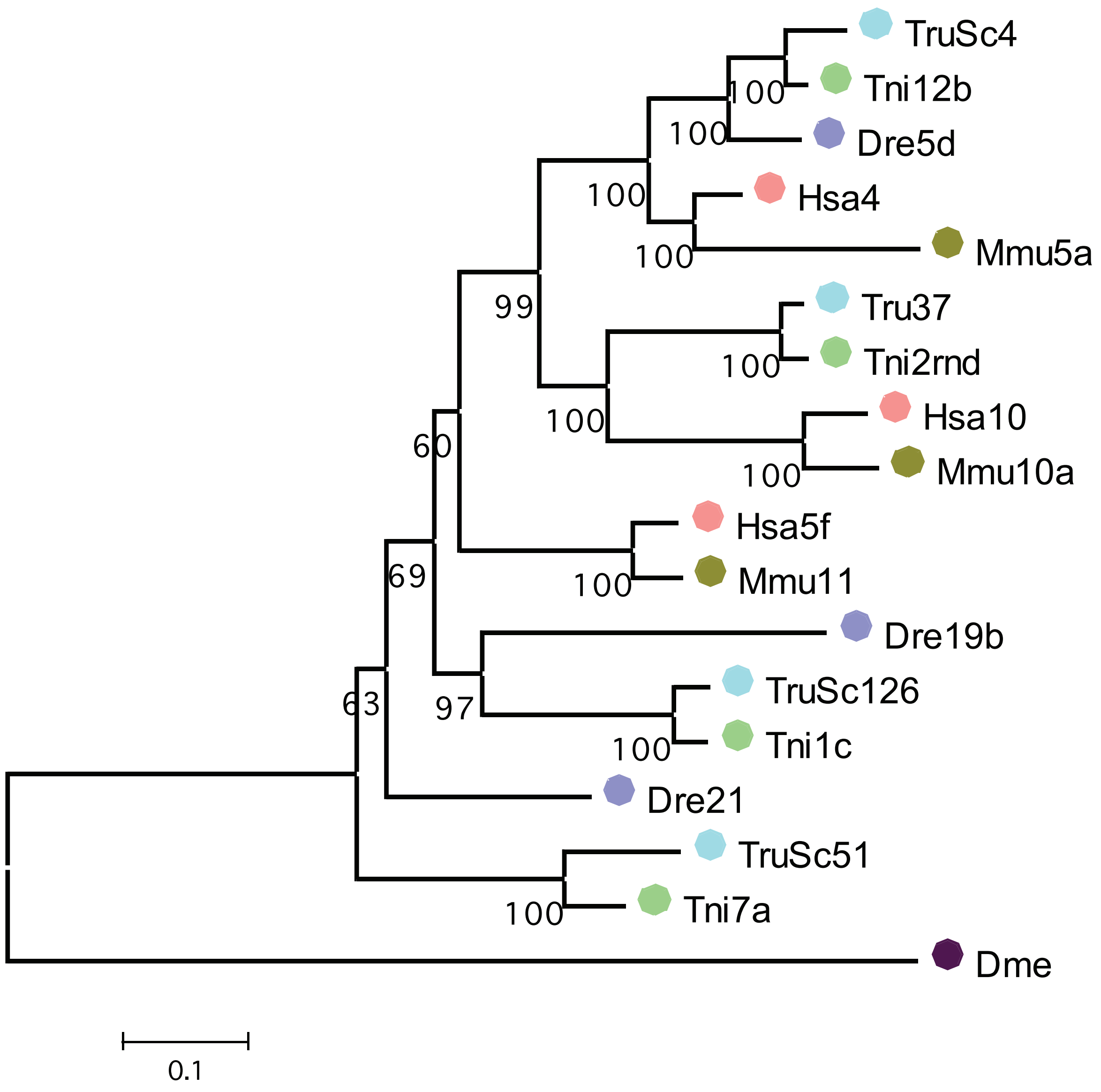
TruSc4
Tni12b
Dre5d
Hsa4
Mmu5a
Tru37
Tni2rnd
Hsa10
Mmu10a
Hsa5f
Mmu11
Dre19b
TruSc126
Tni1c
Dre21
TruSc51
Tni7a
Dme
100
100
100
100
99
100
100
100
60
100
69
97
63
100
100
0.1

Ankyrin NJ

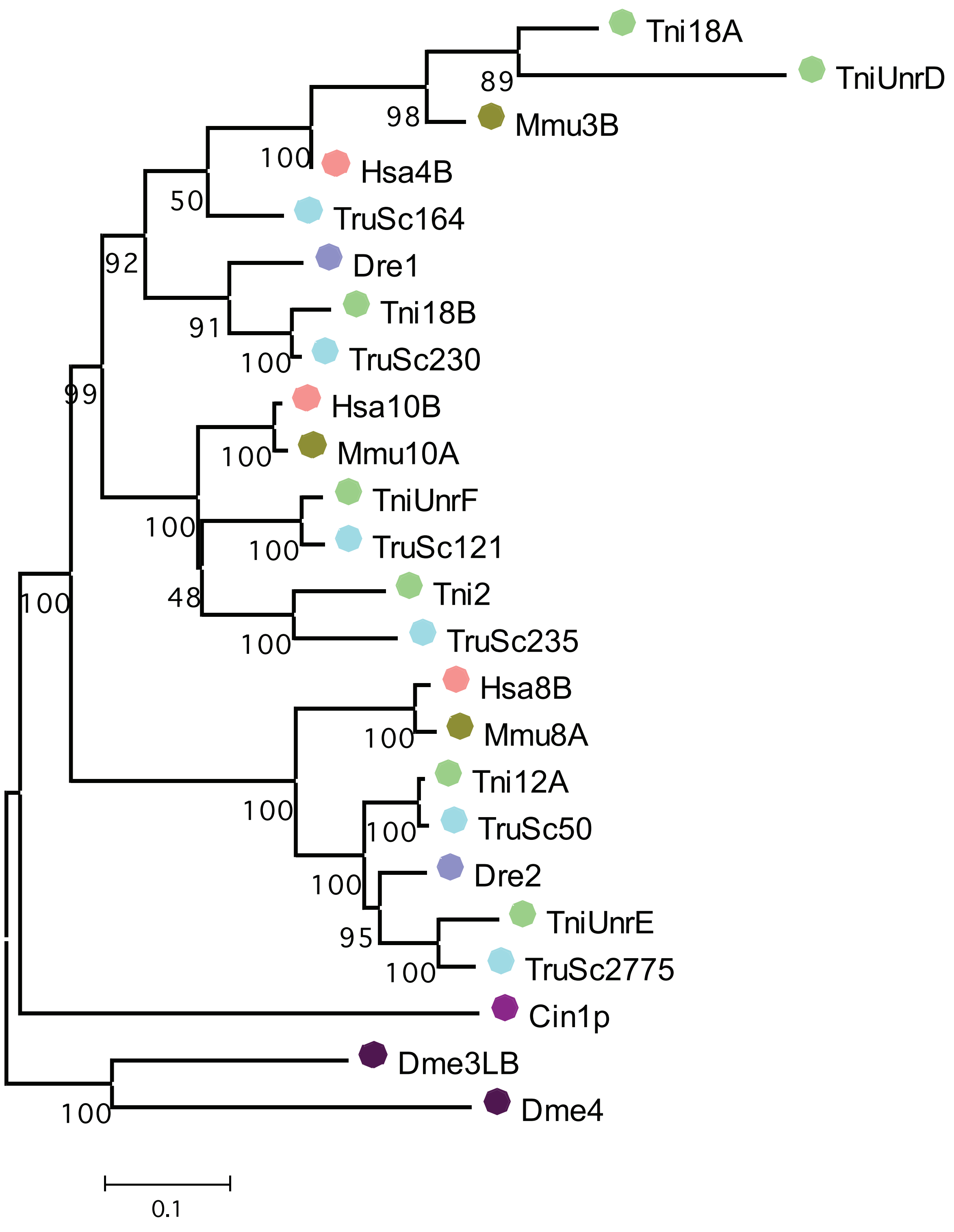
Tni18A
TniUnrD
Mmu3B
Hsa4B
TruSc164
Dre1
Tni18B
TruSc230
Hsa10B
Mmu10A
TniUnrF
TruSc121
Tni2
TruSc235
Hsa8B
Mmu8A
Tni12A
TruSc50
Dre2
TniUnrE
TruSc2775
Cin1p
Dme3LB
Dme4
89
98
100
50
92
91
100
99
100
100
100
48
100
100
100
100
100
100
95
100
100
100
0.1

ANX NJ

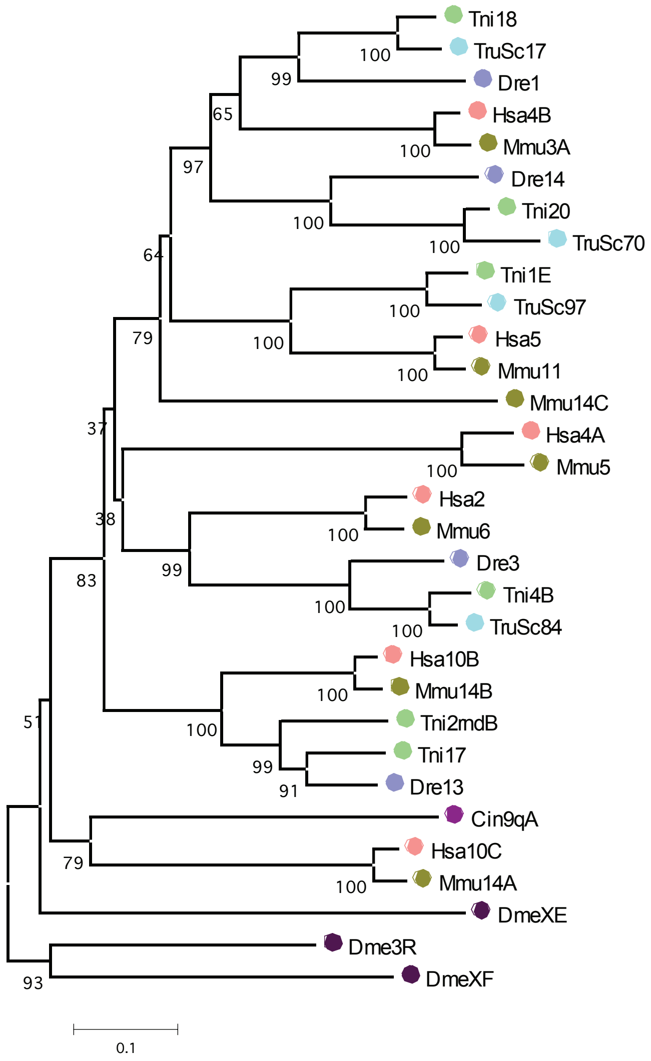
Tni18
TruSc17
Dre1
Hsa4B
Mmu3A
Dre14
Tni20
TruSc70
Tni1E
TruSc97
Hsa5
Mmu11
Mmu14C
Hsa4A
Mmu5
Hsa2
Mmu6
Dre3
Tni4B
TruSc84
Hsa10B
Mmu14B
Tni2mdB
Tni17
Dre13
Cin9qA
Hsa10C
Mmu14A
DmeXE
Dme3R
DmeXF
100
99
65
100
97
100
100
64
100
79
100
100
37
100
38
100
99
100
100
83
100
100
51
99
91
79
100
93
0.1

AP3 NJ

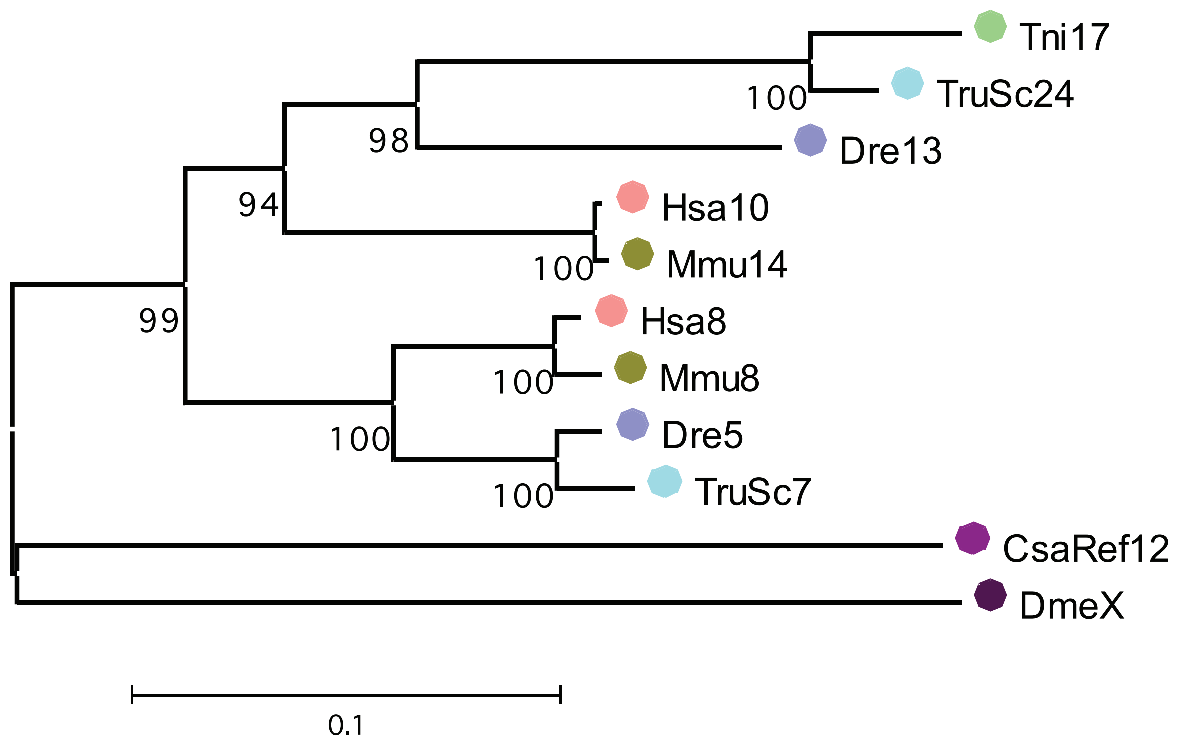

Tni17
TruSc24
Dre13
Hsa10
Mmu14
Hsa8
Mmu8
Dre5
TruSc7
CsaRef12
DmeX
100
98
94
100
99
100
100
100
0.1

CNNM1 NJ

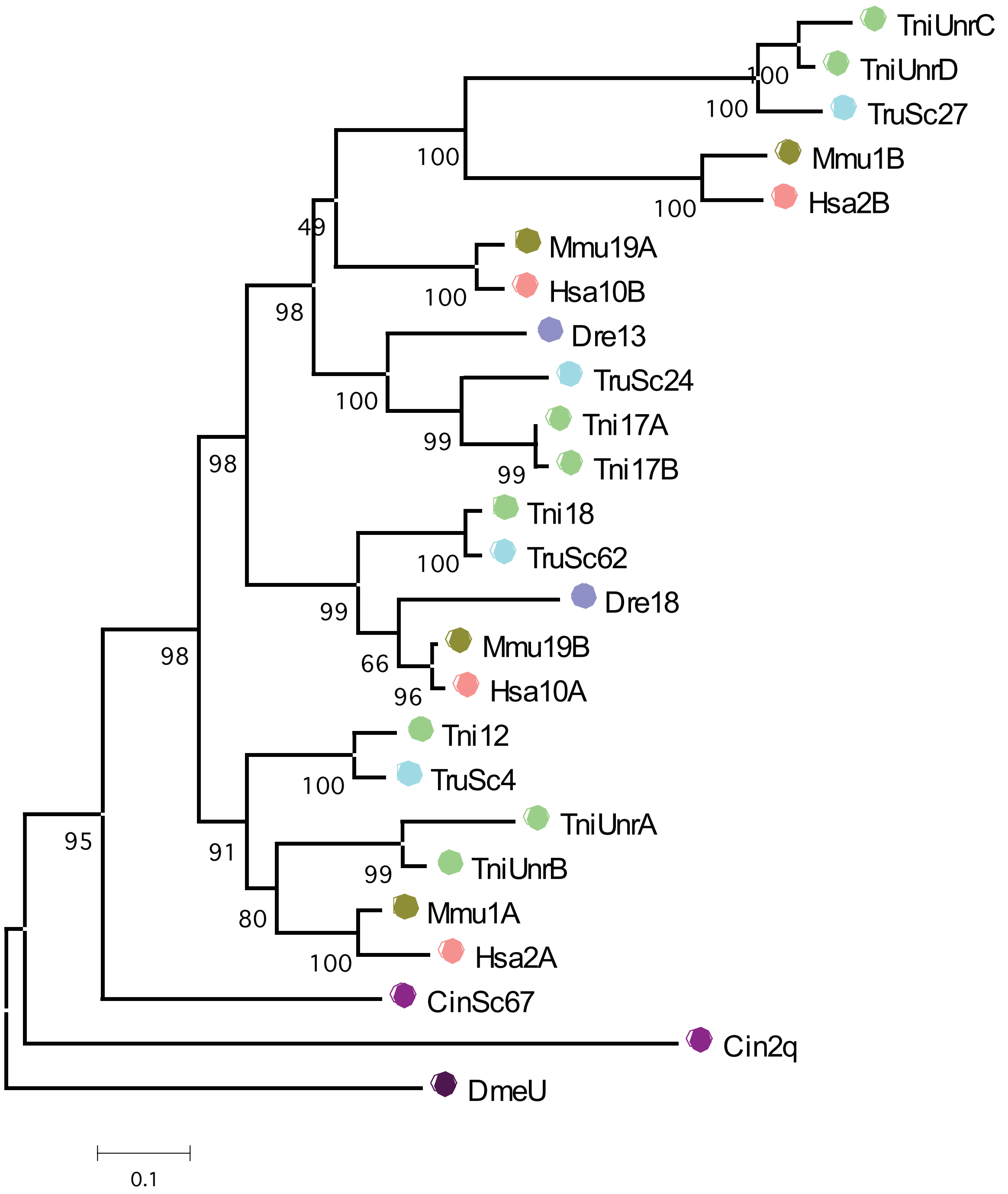
TniUnrC
TniUnrD
TruSc27
Mmu1B
Hsa2B
Mmu19A
Hsa10B
Dre13
TruSc24
Tni17A
Tni17B
Tni18
TruSc62
Dre18
Mmu19B
Hsa10A
Tni12
TruSc4
TniUnrA
TniUnrB
Mmu1A
Hsa2A
CinSc67
Cin2q
DmeU
100
100
100
100
49
100
98
100
99
99
98
100
99
99
66
96
98
100
95
91
99
80
100
0.1

DUSP NJ

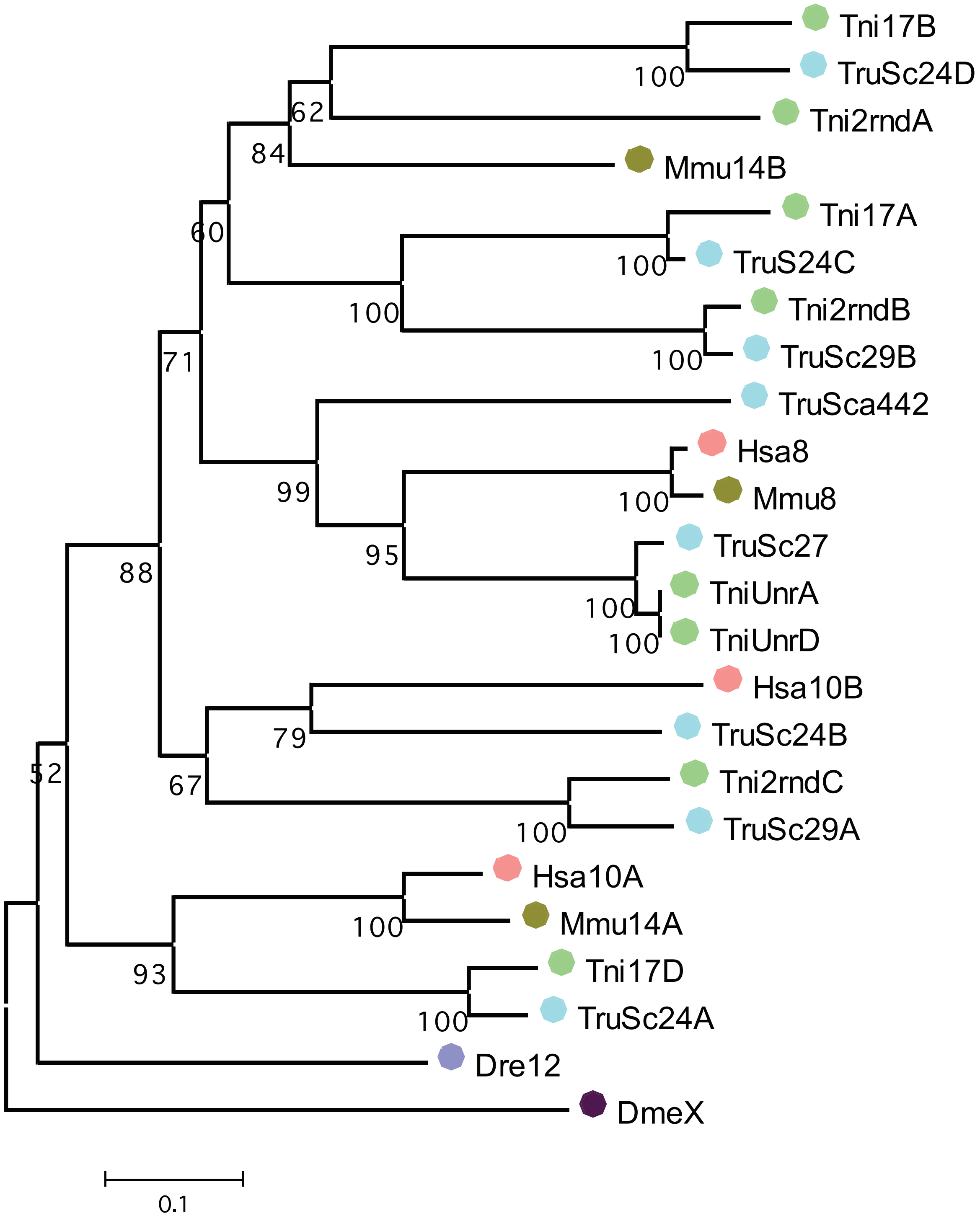
Tni17B
TruSc24D
Tni2rndA
Mmu14B
Tni17A
TruS24C
Tni2rndB
TruSc29B
TruSca442
Hsa8
Mmu8
TruSc27
TniUnrA
TniUnrD
Hsa10B
TruSc24B
Tni2rndC
TruSc29A
Hsa10A
Mmu14A
Tni17D
TruSc24A
Dre12
DmeX
100
62
84
60
100
100
100
71
99
100
95
100
100
88
79
67
100
52
100
93
100
0.1

FGFR NJ

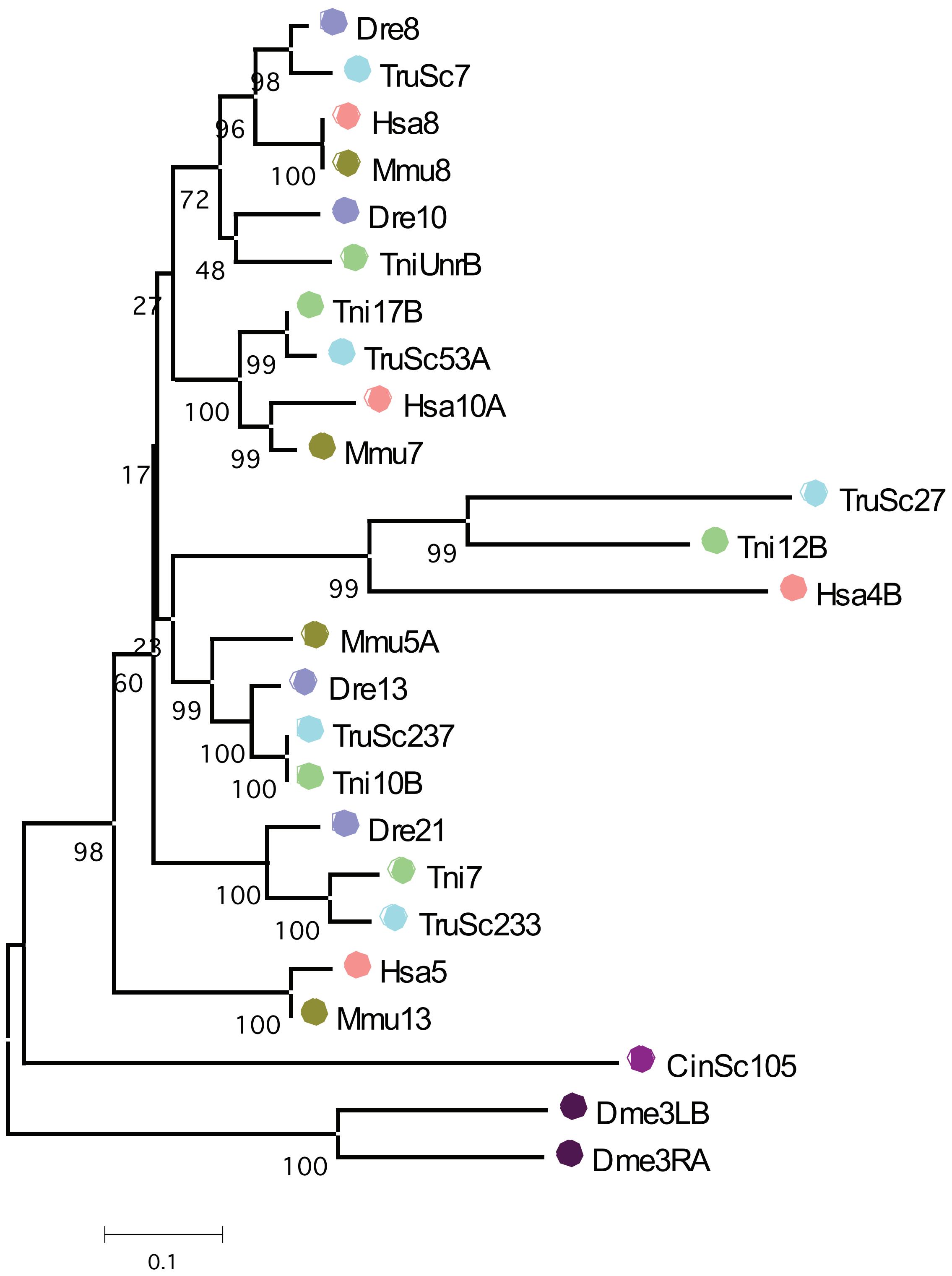
Dre8
TruSc7
Hsa8
Mmu8
Dre10
TniUnrB
Tni17B
TruSc53A
Hsa10A
Mmu7
TruSc27
Tni12B
Hsa4B
Mmu5A
Dre13
TruSc237
Tni10B
Dre21
Tni7
TruSc233
Hsa5
Mmu13
CinSc105
Dme3LB
Dme3RA
98
96
100
72
48
27
99
100
99
17
99
99
23
60
99
100
100
98
100
100
100
100
0.1

HNRP NJ

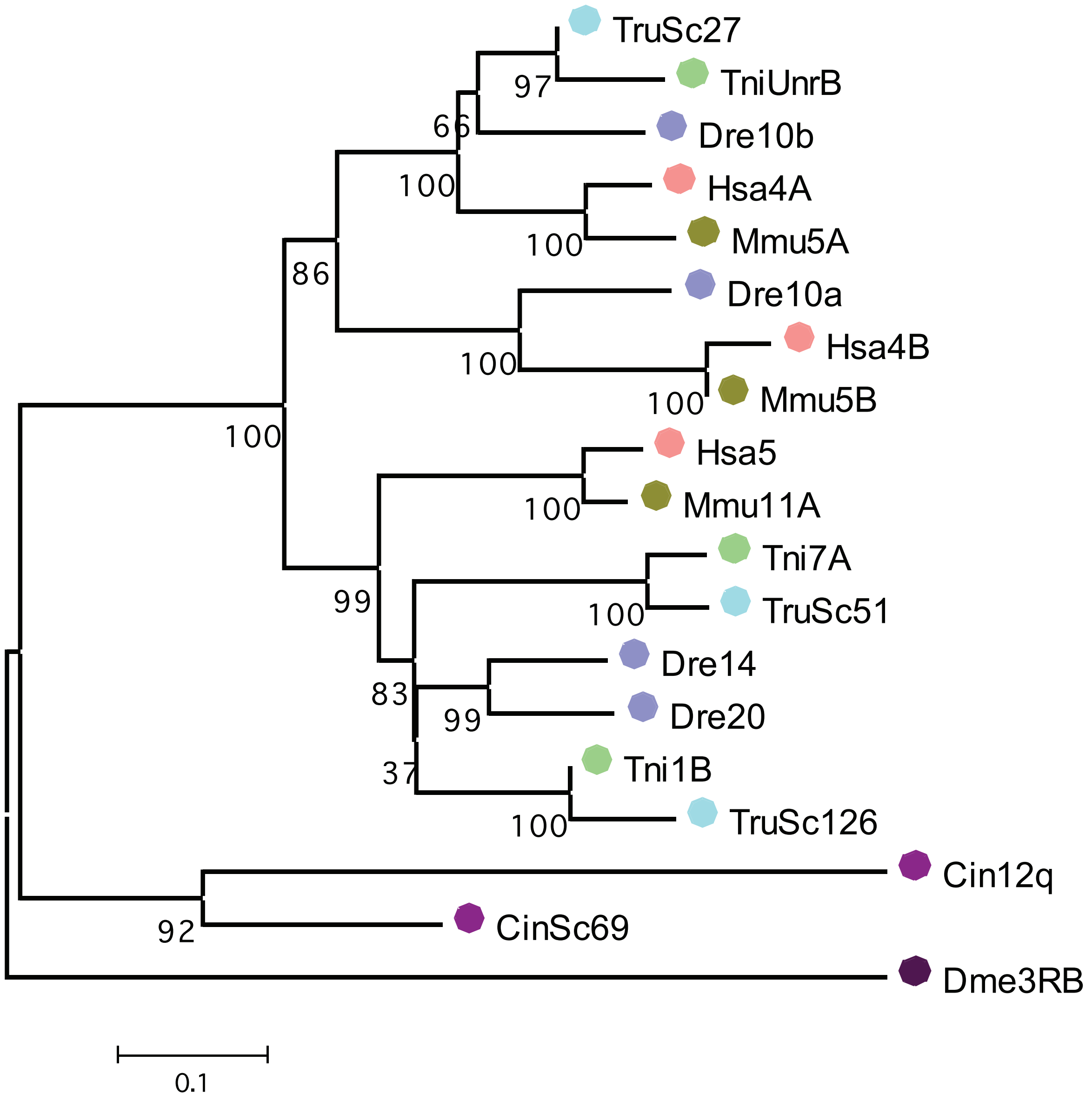
TruSc27
TniUnrB
Dre10b
Hsa4A
Mmu5A
Dre10a
Hsa4B
Mmu5B
Hsa5
Mmu11A
Tni7A
TruSc51
Dre14
Dre20
Tni1B
TruSc126
Cin12q
CinSc69
Dme3RB
97
66
100
100
86
100
100
100
100
99
100
83
99
37
100
92
0.1

LGI NJ

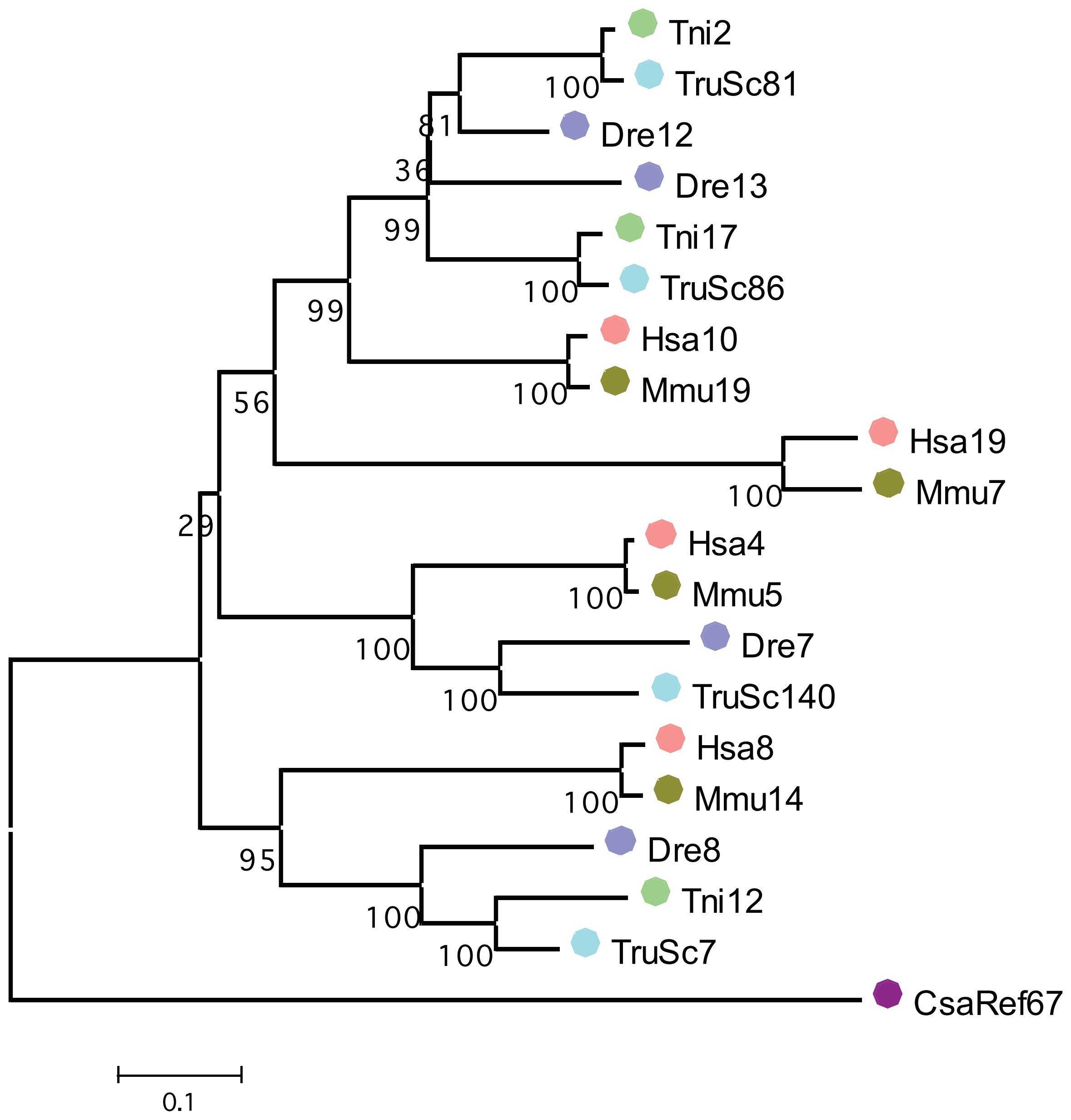
Tni2
TruSc81
Dre12
Dre13
Tni17
TruSc86
Hsa10
Mmu19
Hsa19
Mmu7
Hsa4
Mmu5
Dre7
TruSc140
Hsa8
Mmu14
Dre8
Tni12
TruSc7
CsaRef67
100
81
36
99
100
99
100
56
100
29
100
100
100
100
95
100
100
0.1

MAX NJ

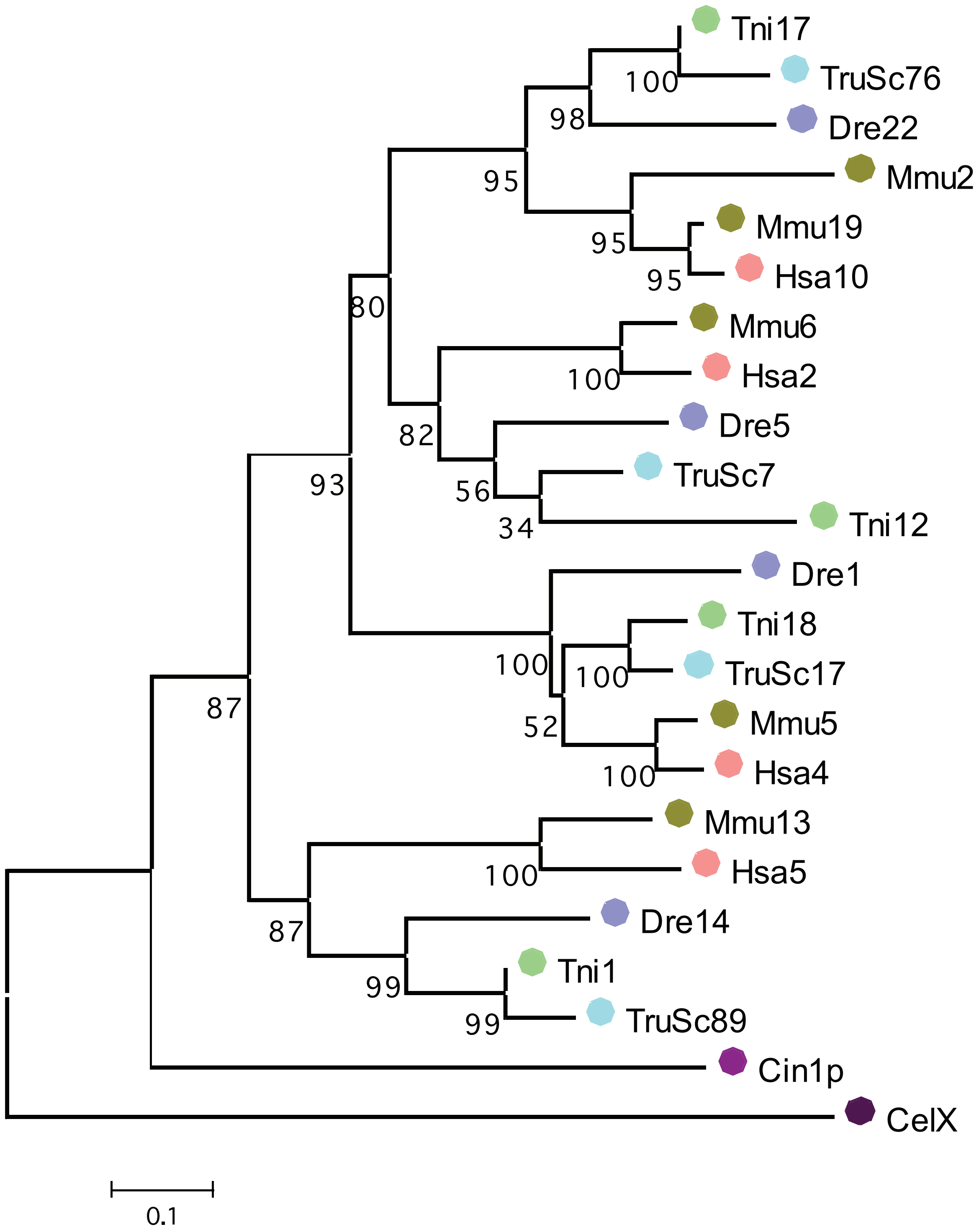

Tni17
TruSc76
Dre22
Mmu2
Mmu19
Hsa10
Mmu6
Hsa2
Dre5
TruSc7
Tni12
Dre1
Tni18
TruSc17
Mmu5
Hsa4
Mmu13
Hsa5
Dre14
Tni1
TruSc89
Cin1p
CelX
100
98
95
95
95
80
100
82
56
34
93
100
100
52
100
87
100
87
99
99
0.1

Mitogen NJ

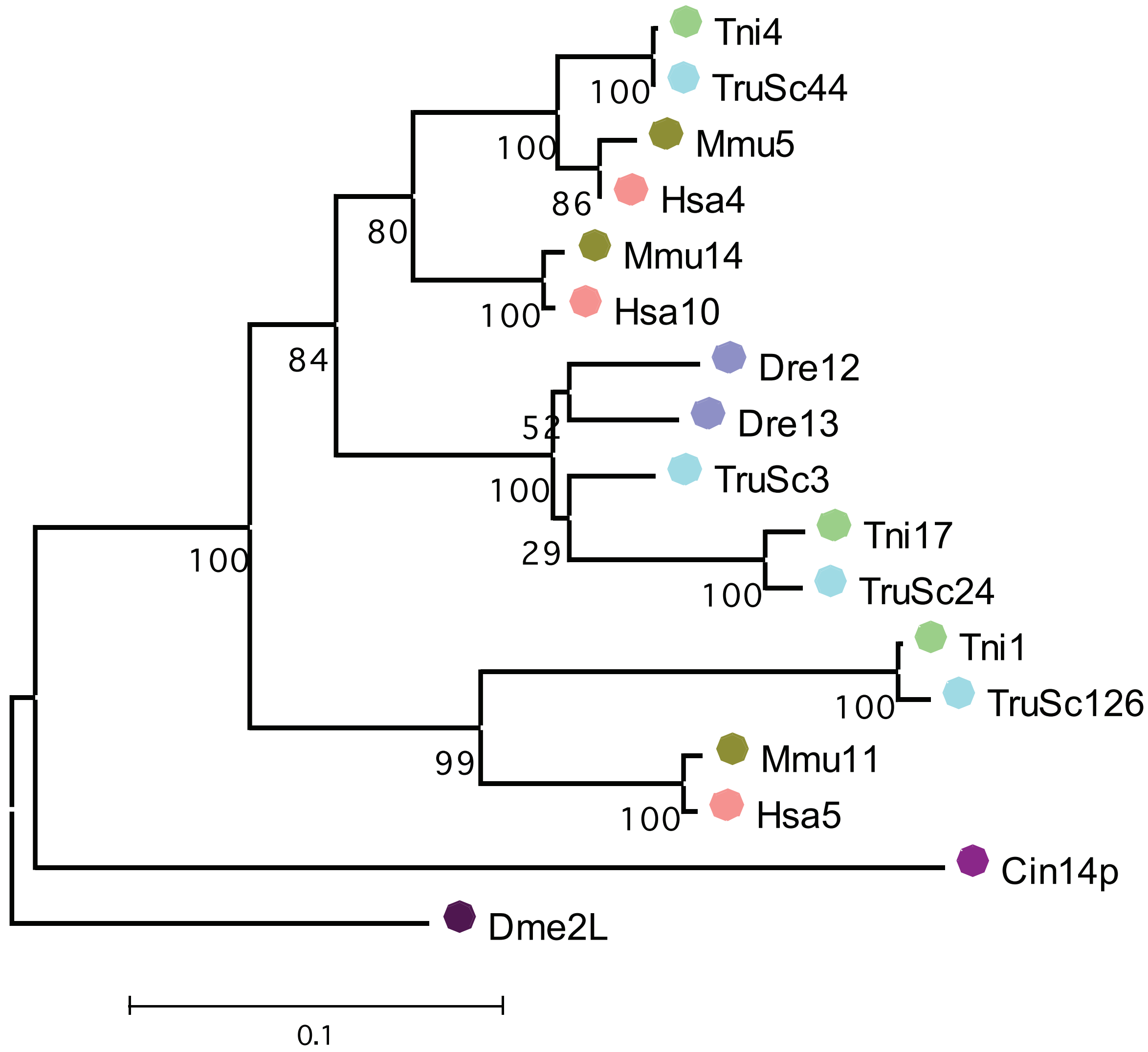

Tni4
TruSc44
100
Mmu5
100
Hsa4
86
80
Mmu14
Hsa10
100
84
Dre12
Dre13
52
TruSc3
100
Tni17
29
TruSc24
100
100
Tni1
TruSc126
100
99
Mmu11
Hsa5
100
Cin14p
Dme2L
0.1

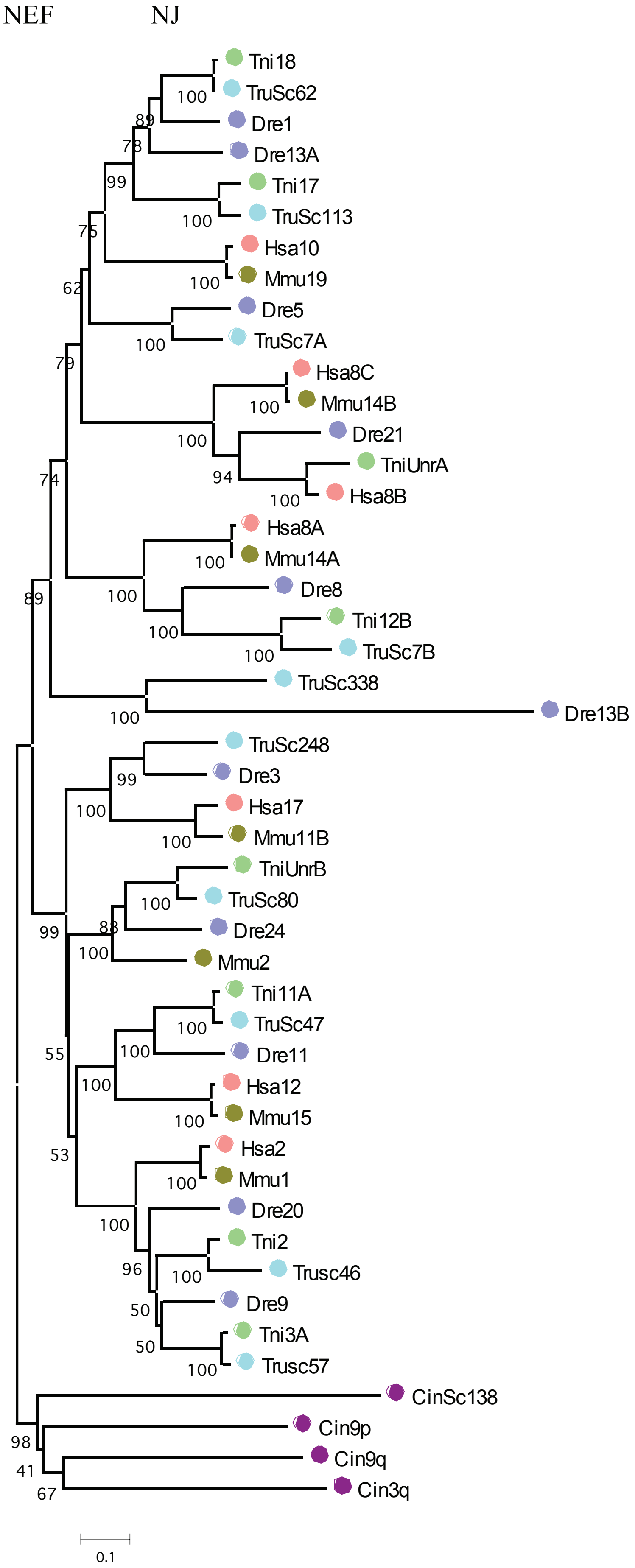

NEF
NJ
Tni18
TruSc62
Dre1
Dre13A
Tni17
TruSc113
Hsa10
Mmu19
Dre5
TruSc7A
Hsa8C
Mmu14B
Dre21
TniUnrA
Hsa8B
Hsa8A
Mmu14A
Dre8
Tni12B
TruSc7B
TruSc338
Dre13B
TruSc248
Dre3
Hsa17
Mmu11B
TniUnrB
TruSc80
Dre24
Mmu2
Tni11A
TruSc47
Dre11
Hsa12
Mmu15
Hsa2
Mmu1
Dre20
Tni2
Trusc46
Dre9
Tni3A
Trusc57
CinSc138
Cin9p
Cin9q
Cin3q
0.1

NKR NJ

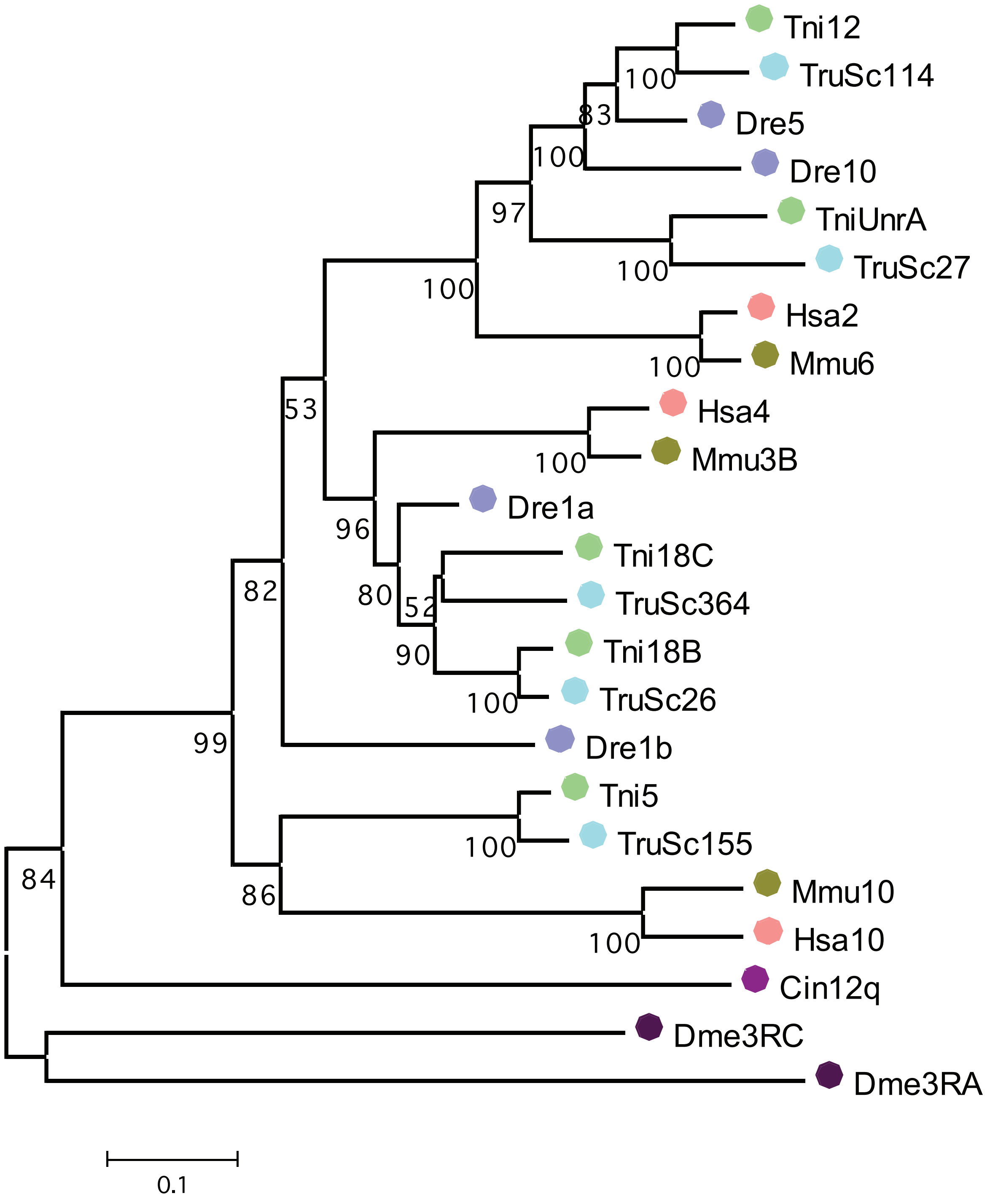
Tni12
TruSc114
Dre5
Dre10
TniUnrA
TruSc27
Hsa2
Mmu6
Hsa4
Mmu3B
Dre1a
Tni18C
TruSc364
Tni18B
TruSc26
Dre1b
Tni5
TruSc155
Mmu10
Hsa10
Cin12q
Dme3RC
Dme3RA
100
83
100
97
100
100
100
53
100
96
80
52
90
100
82
99
100
86
100
84
0.1

Oxoglut NJ

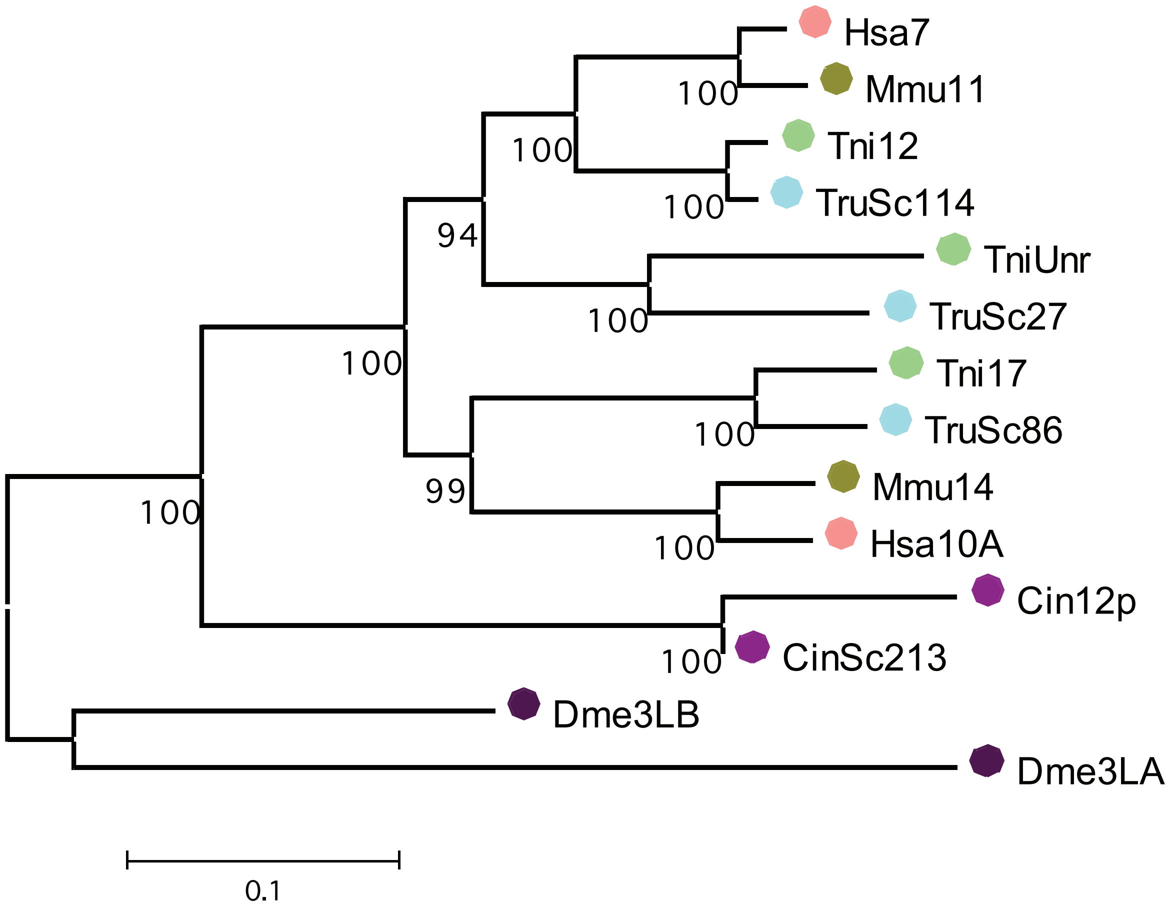

Hsa7
Mmu11
Tni12
TruSc114
TniUnr
TruSc27
Tni17
TruSc86
Mmu14
Hsa10A
Cin12p
CinSc213
Dme3LB
Dme3LA
100
100
100
94
100
100
100
99
100
100
100
0.1

PDLIM3 NJ

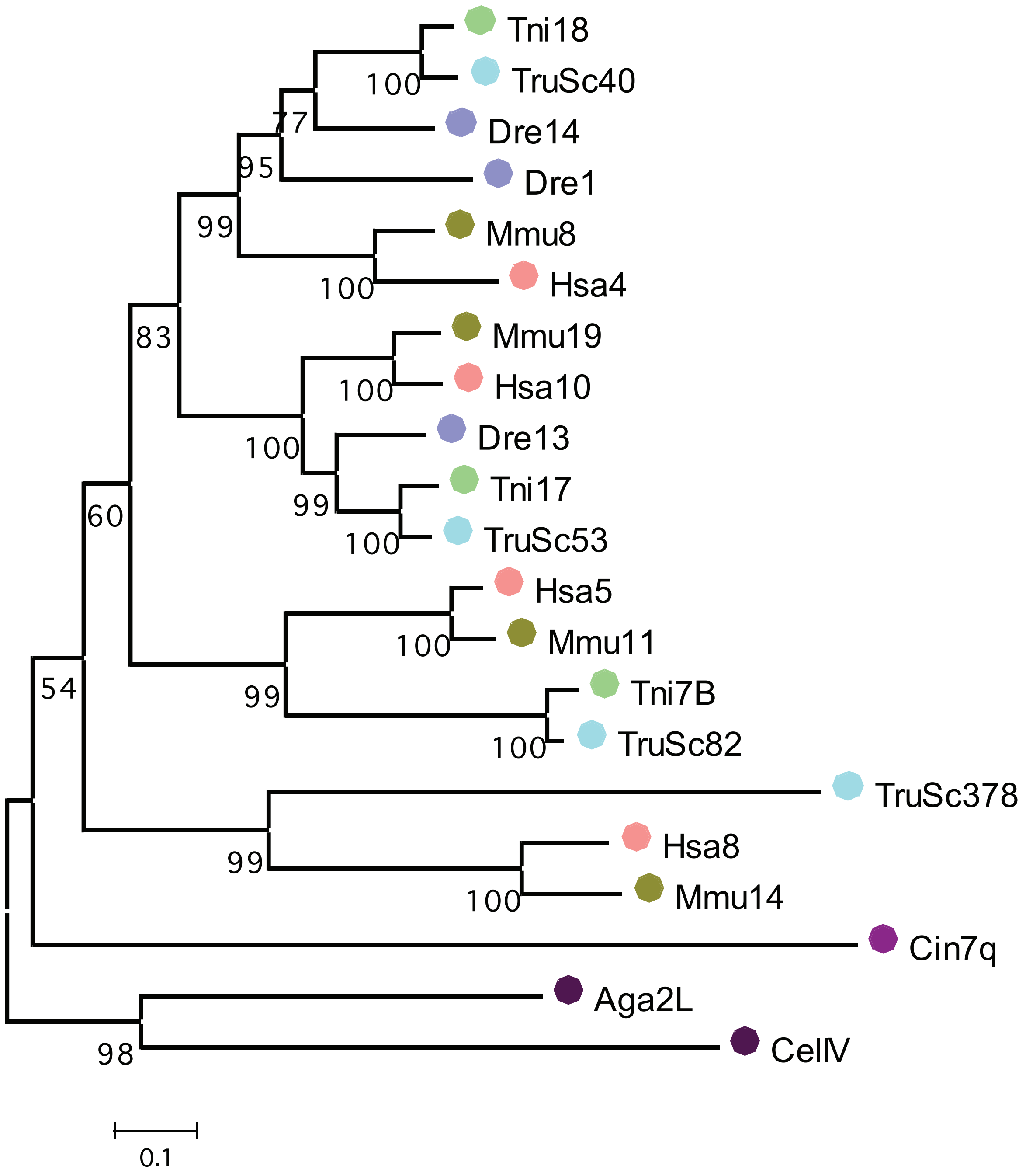
Tni18
TruSc40
Dre14
Dre1
Mmu8
Hsa4
Mmu19
Hsa10
Dre13
Tni17
TruSc53
Hsa5
Mmu11
Tni7B
TruSc82
TruSc378
Hsa8
Mmu14
Cin7q
Aga2L
CelIV
100
77
95
99
100
83
100
100
99
100
60
100
99
100
54
99
100
98
0.1

PX19 NJ

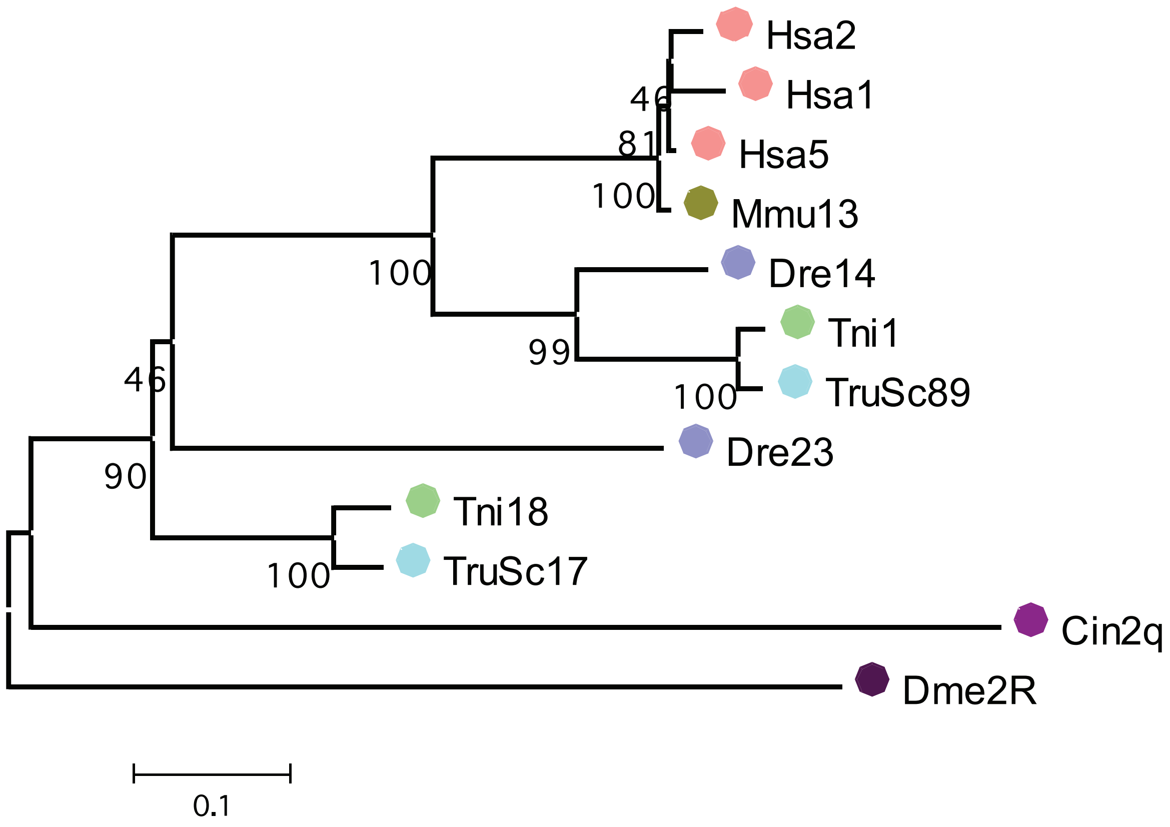
Hsa2
Hsa1
Hsa5
Mmu13
Dre14
Tni1
TruSc89
Dre23
Tni18
TruSc17
Cin2q
Dme2R
46
81
100
100
99
100
46
90
100
0.1

SAMD8 NJ

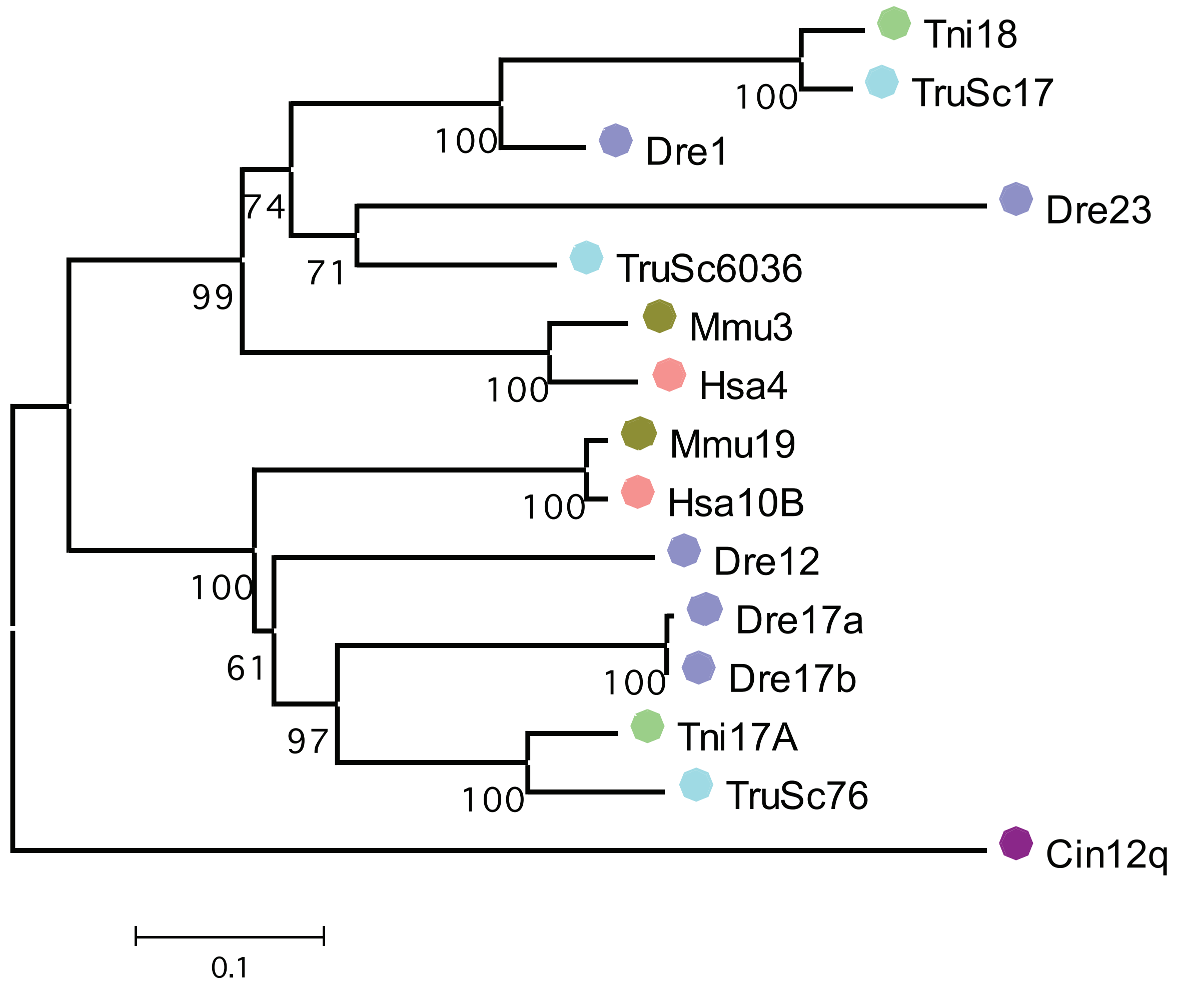

SAMD8Final2SUB2

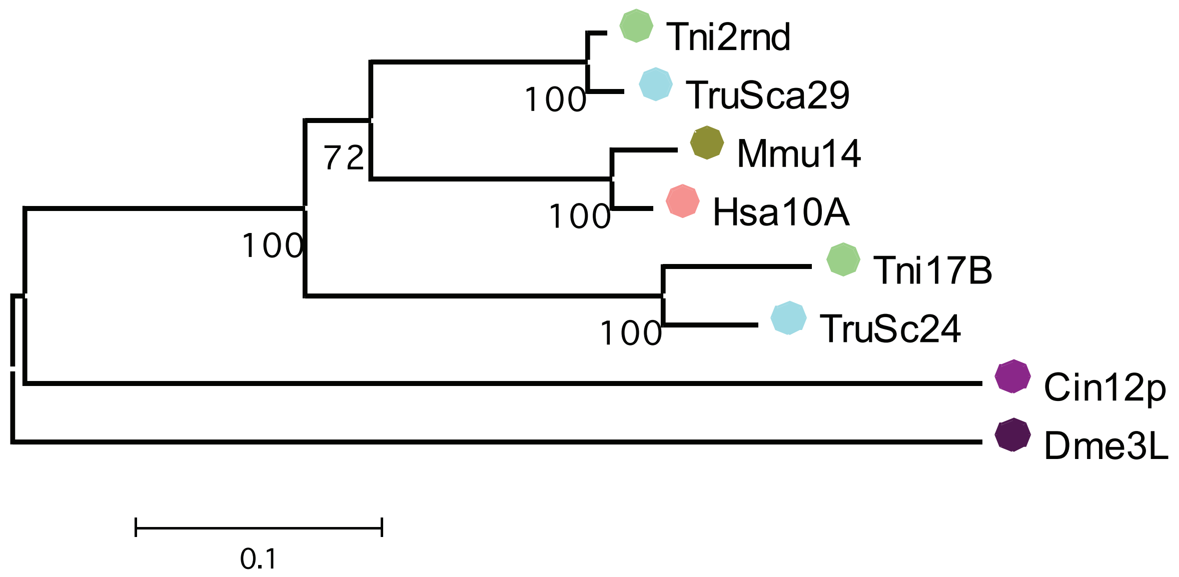

SFRP NJ

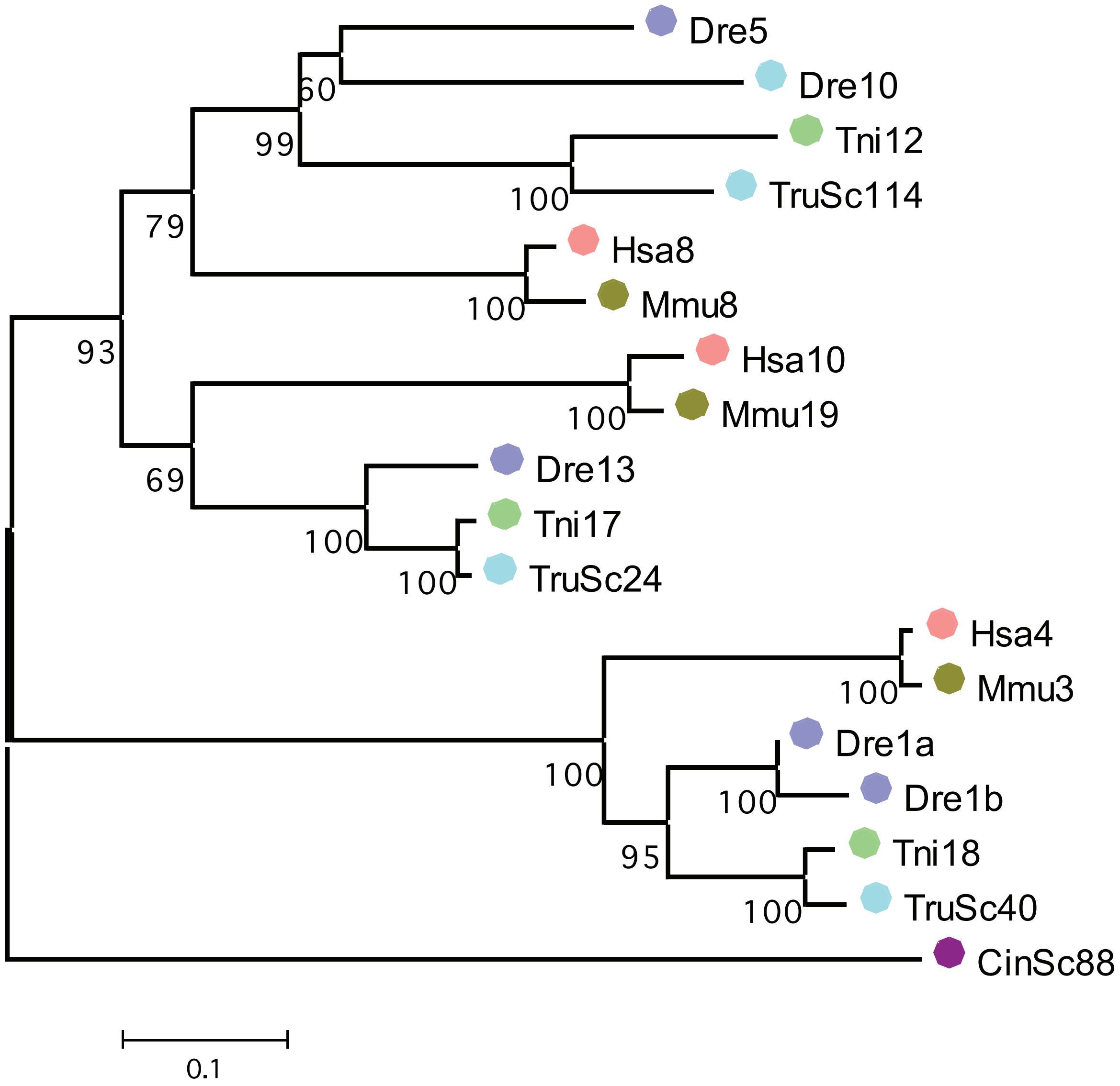
Dre5
Dre10
Tni12
TruSc114
Hsa8
Mmu8
Hsa10
Mmu19
Dre13
Tni17
TruSc24
Hsa4
Mmu3
Dre1a
Dre1b
Tni18
TruSc40
CinSc88
60
99
100
79
100
93
100
69
100
100
100
100
100
95
100
0.1

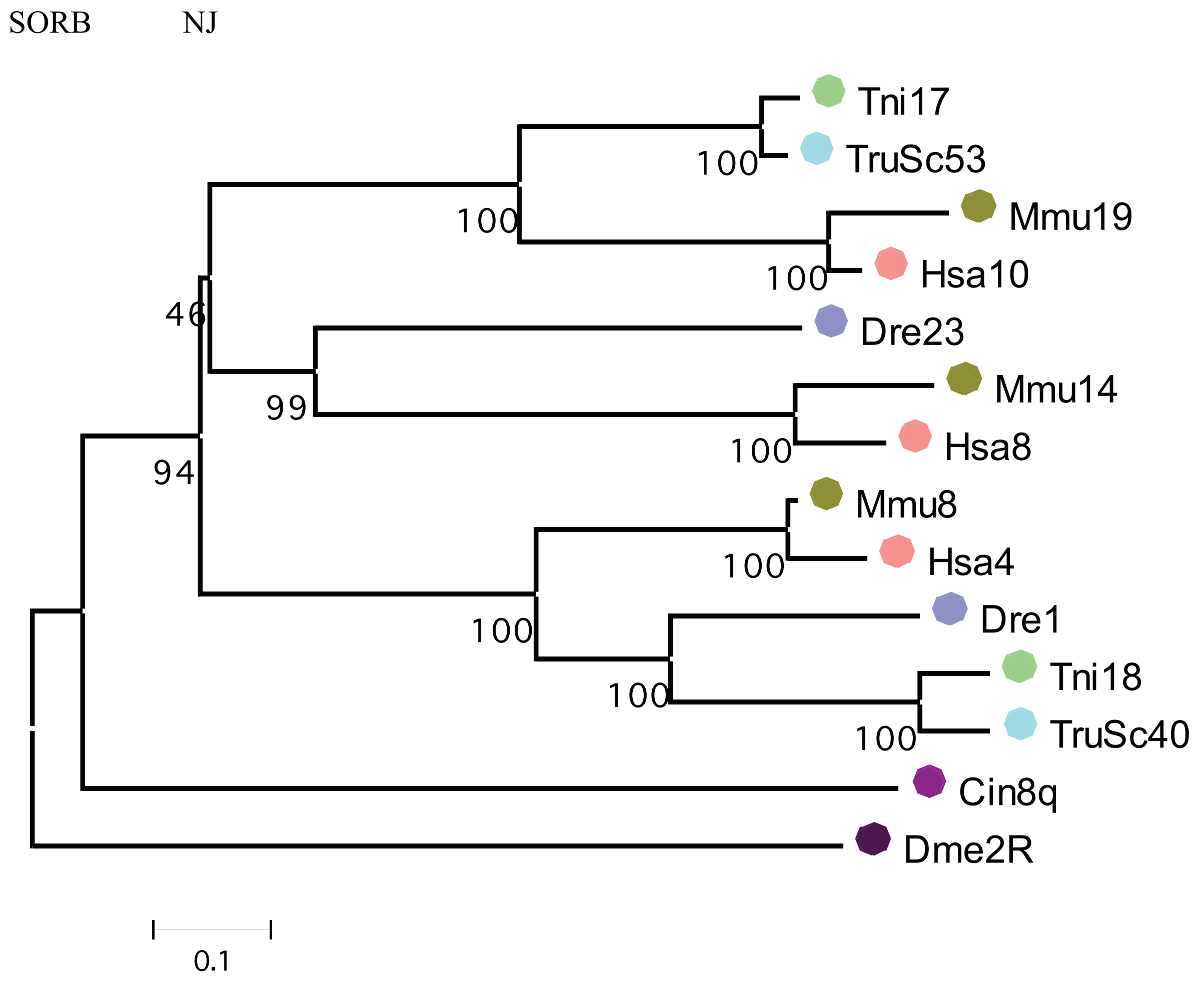
SORB
NJ
Tni17
TruSc53
100
100
Mmu19
Hsa10
100
46
Dre23
99
Mmu14
Hsa8
100
94
Mmu8
100
Hsa4
100
Dre1
100
Tni18
TruSc40
100
Cin8q
Dme2R
0.1

TIA NJ

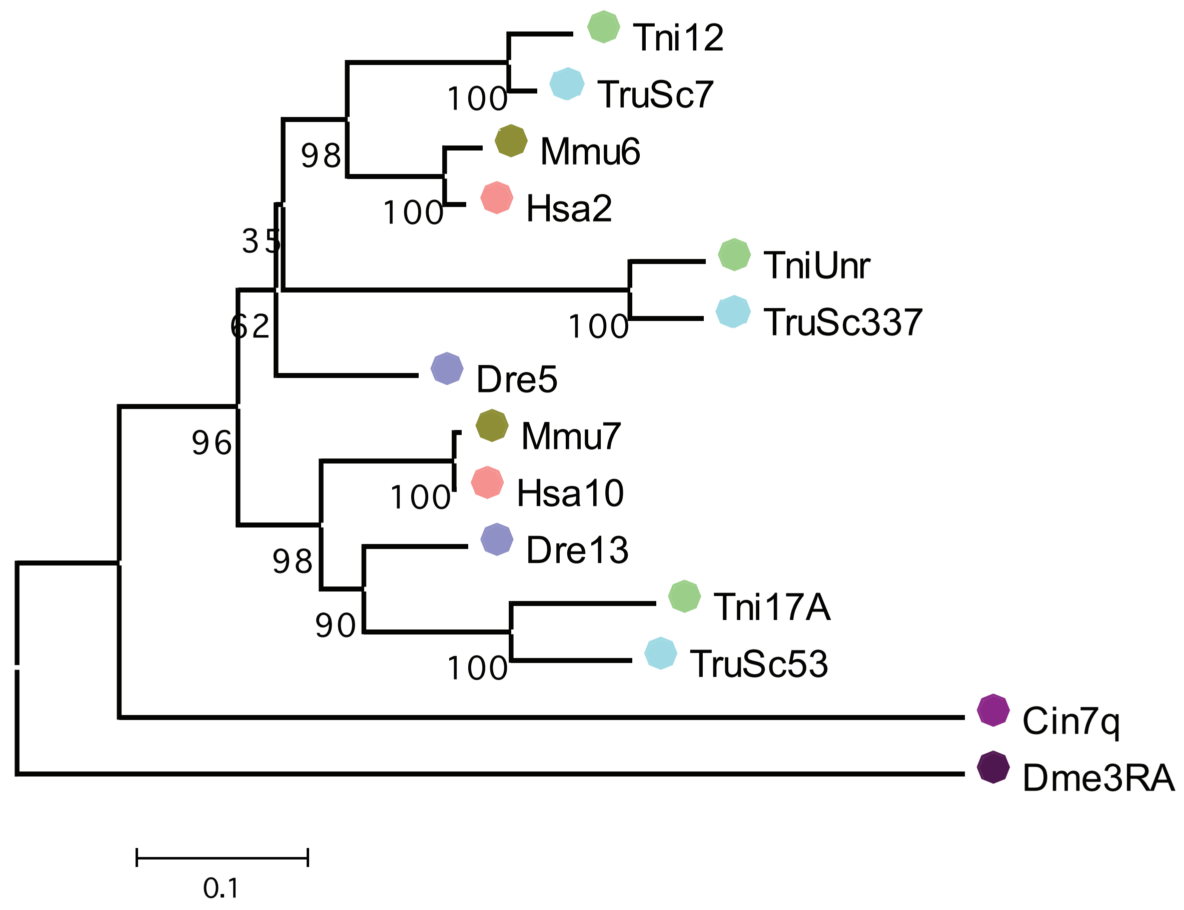

Tni12
TruSc7
100
98
Mmu6
Hsa2
100
35
TniUnr
TruSc337
100
62
Dre5
96
Mmu7
Hsa10
100
Dre13
98
90
Tni17A
TruSc53
100
Cin7q
Dme3RA
0.1

TSPAN NJ

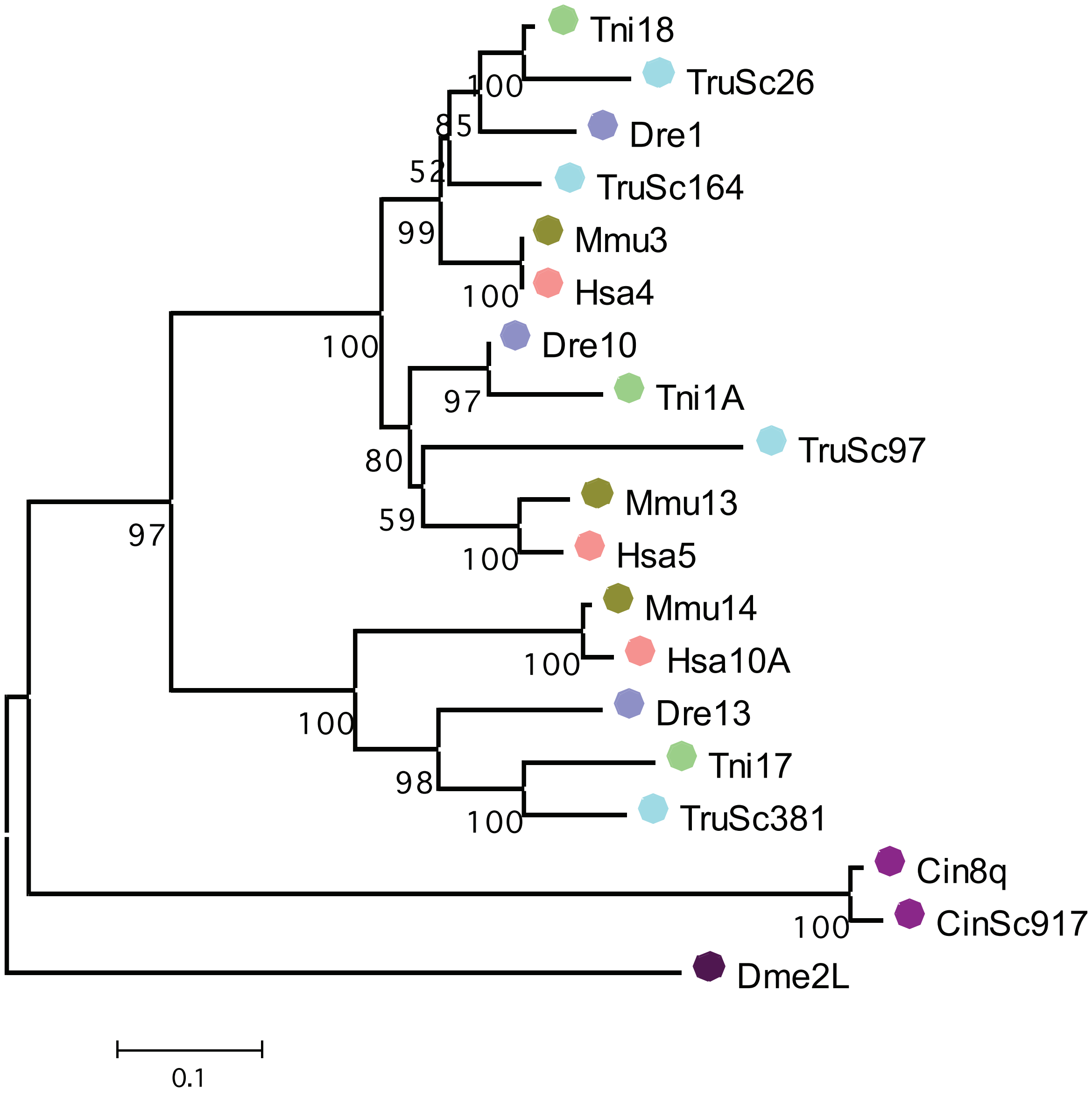

Tni18
TruSc26
Dre1
TruSc164
Mmu3
Hsa4
Dre10
Tni1A
TruSc97
Mmu13
Hsa5
Mmu14
Hsa10A
Dre13
Tni17
TruSc381
Cin8q
CinSc917
Dme2L
100
85
52
99
100
100
97
80
59
100
97
100
100
98
100
100
0.1

UNC5 NJ

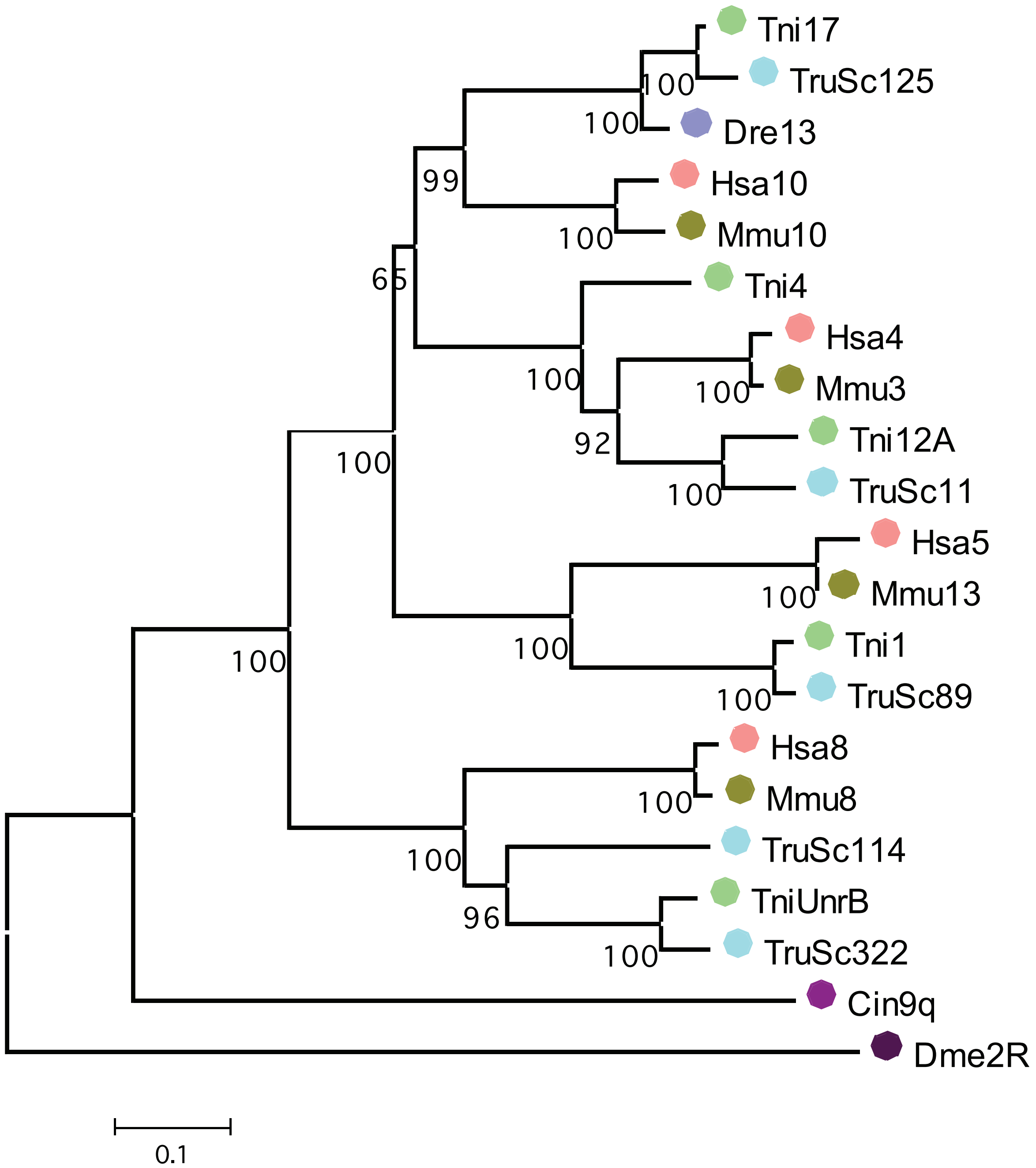
Tni17
TruSc125
Dre13
Hsa10
Mmu10
Tni4
Hsa4
Mmu3
Tni12A
TruSc11
Hsa5
Mmu13
Tni1
TruSc89
Hsa8
Mmu8
TruSc114
TniUnrB
TruSc322
Cin9q
Dme2R
100
100
99
100
65
100
100
92
100
100
100
100
100
100
100
100
96
100
0.1

ZIMP NJ

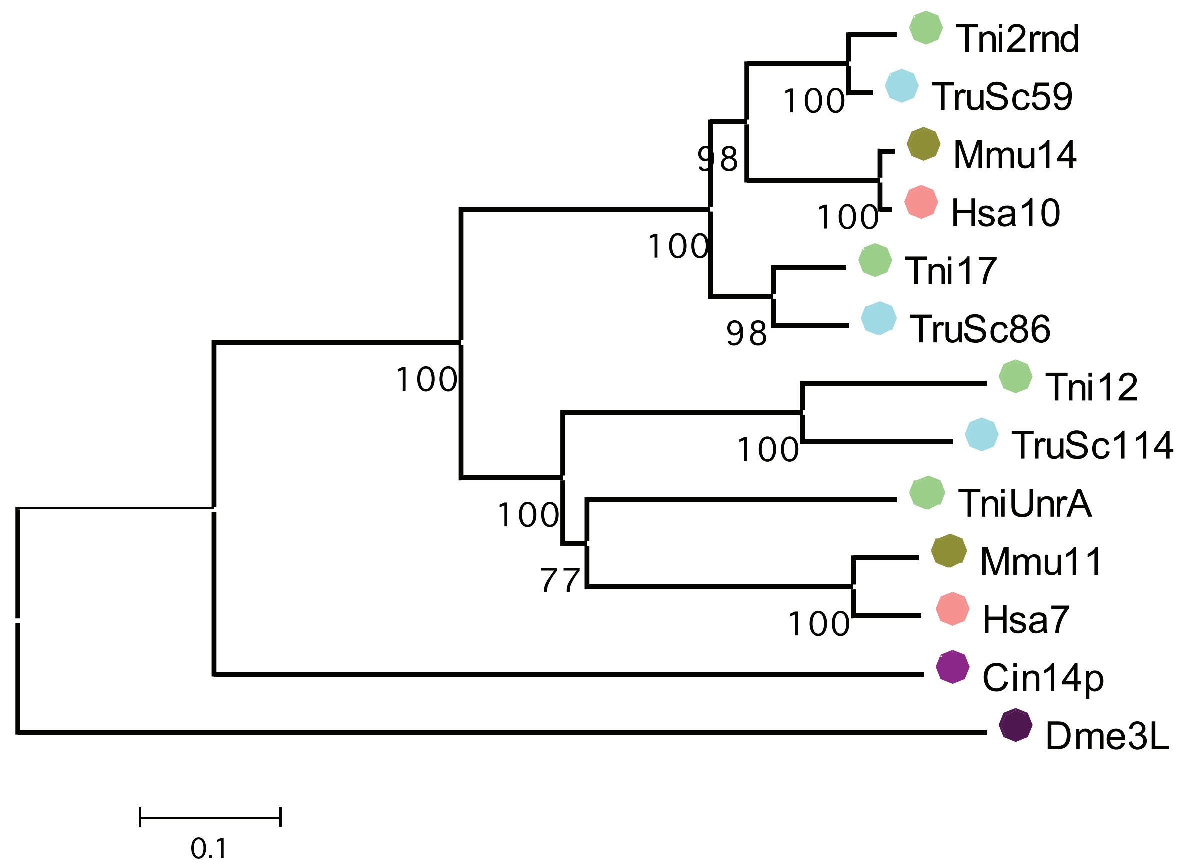

Tni2rnd
TruSc59
Mmu14
Hsa10
Tni17
TruSc86
Tni12
TruSc114
TniUnrA
Mmu11
Hsa7
Cin14p
Dme3L
100
98
100
100
98
100
100
100
77
100
0.1

ZINK NJ

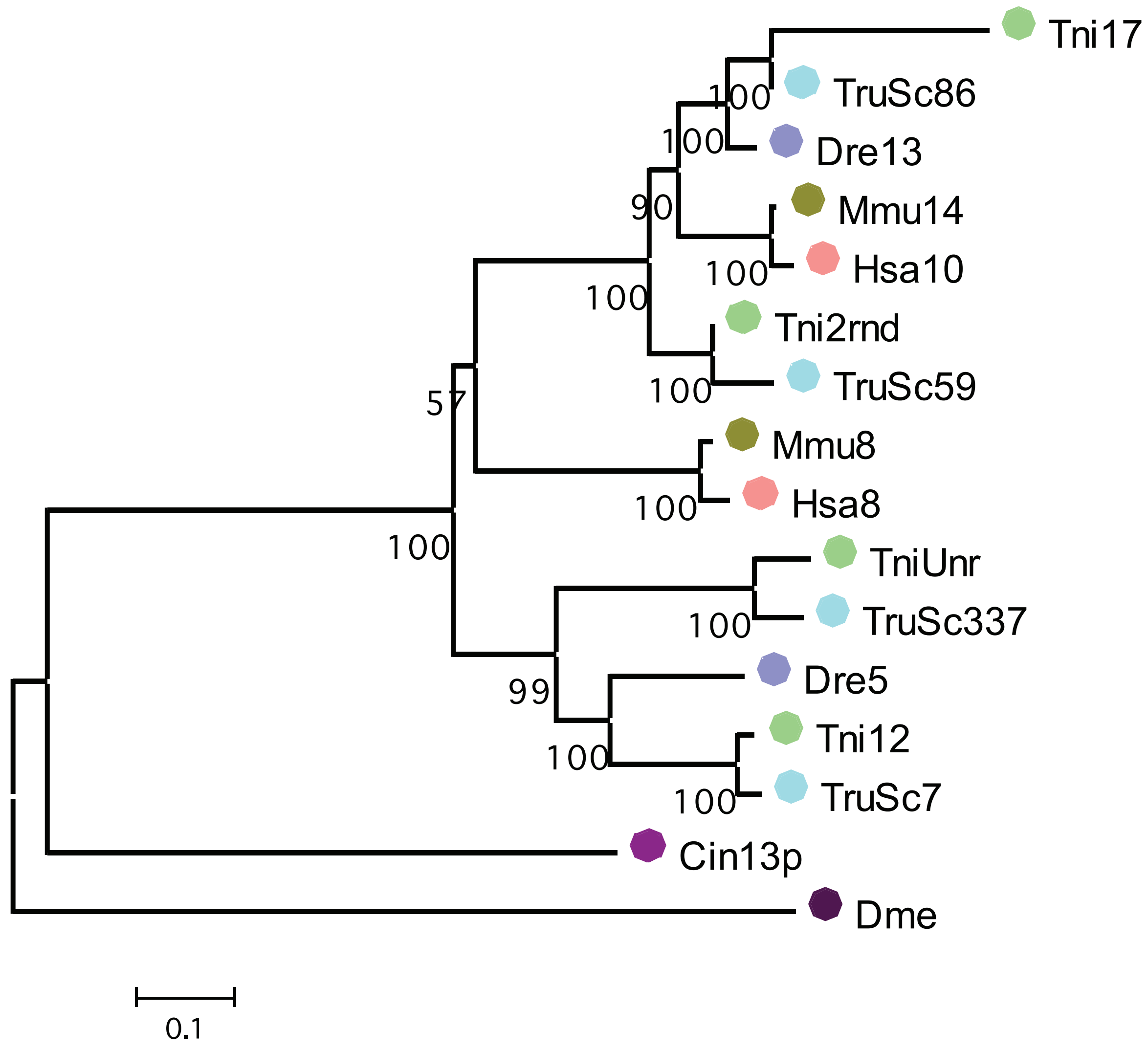

Tni17
TruSc86
Dre13
Mmu14
Hsa10
Tni2rnd
TruSc59
Mmu8
Hsa8
TniUnr
TruSc337
Dre5
Tni12
TruSc7
Cin13p
Dme
100
100
90
100
100
100
57
100
100
100
99
100
100
0.1
