# Supplementary material for: Early vertebrate chromosome duplications and the evolution of the neuropeptide Y receptor gene regions
Source: BMC Evol Biol. 2008 Jun 25;8:184. doi: 10.1186/1471-2148-8-184 (PMC2453138; doi:10.1186/1471-2148-8-184)
Supplement: Additional file 2 — Quartet puzzling trees for the 26 gene families analyzed in detail. [file 1471-2148-8-184-S2.pdf]

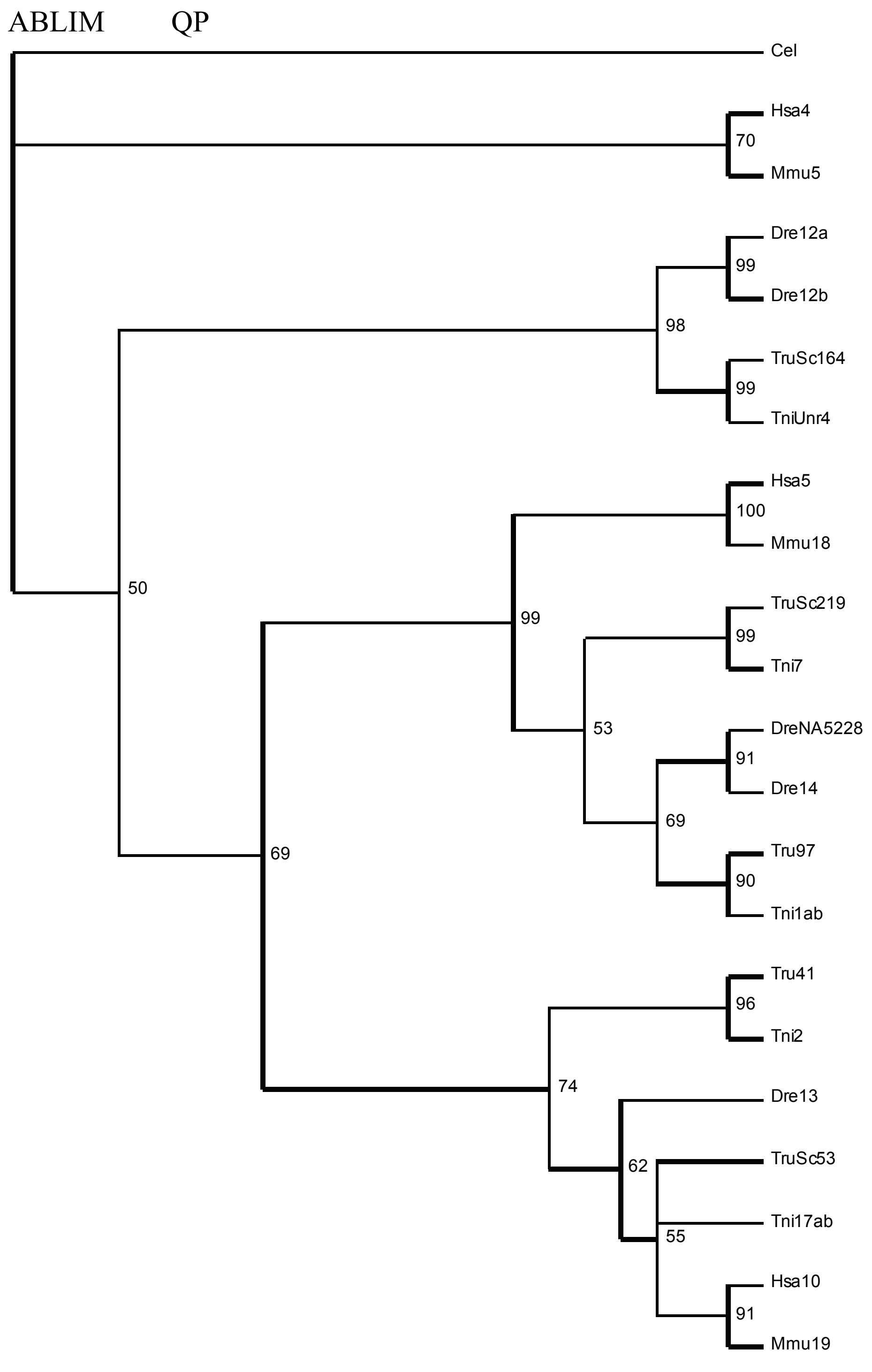
ABLIM
QP
Cel
Hsa4
70
Mmu5
Dre12a
99
Dre12b
98
TruSc164
99
TniUnr4
Hsa5
100
Mmu18
50
TruSc219
99
99
Tni7
53
DreNA5228
91
Dre14
69
Tru97
69
90
Tni1ab
Tru41
96
Tni2
74
Dre13
62
TruSc53
Tni17ab
55
Hsa10
91
Mmu19

ADAMTS QP

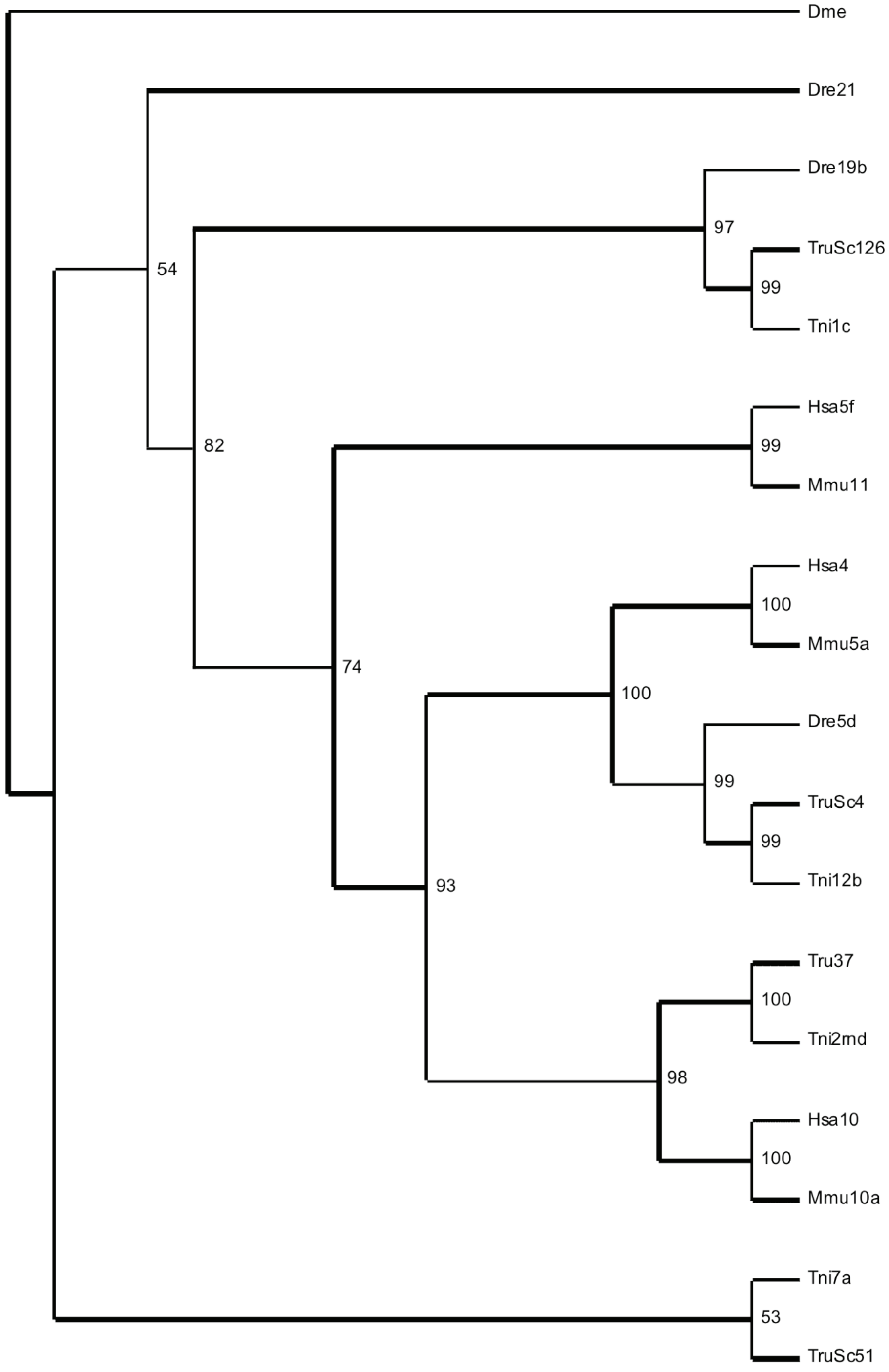
Dme
Dre21
Dre19b
97
TruSc126
99
Tni1c
54
Hsa5f
99
Mmu11
82
Hsa4
100
Mmu5a
74
100
Dre5d
99
TruSc4
99
Tni12b
93
Tru37
100
Tni2md
98
Hsa10
100
Mmu10a
Tni7a
53
TruSc51

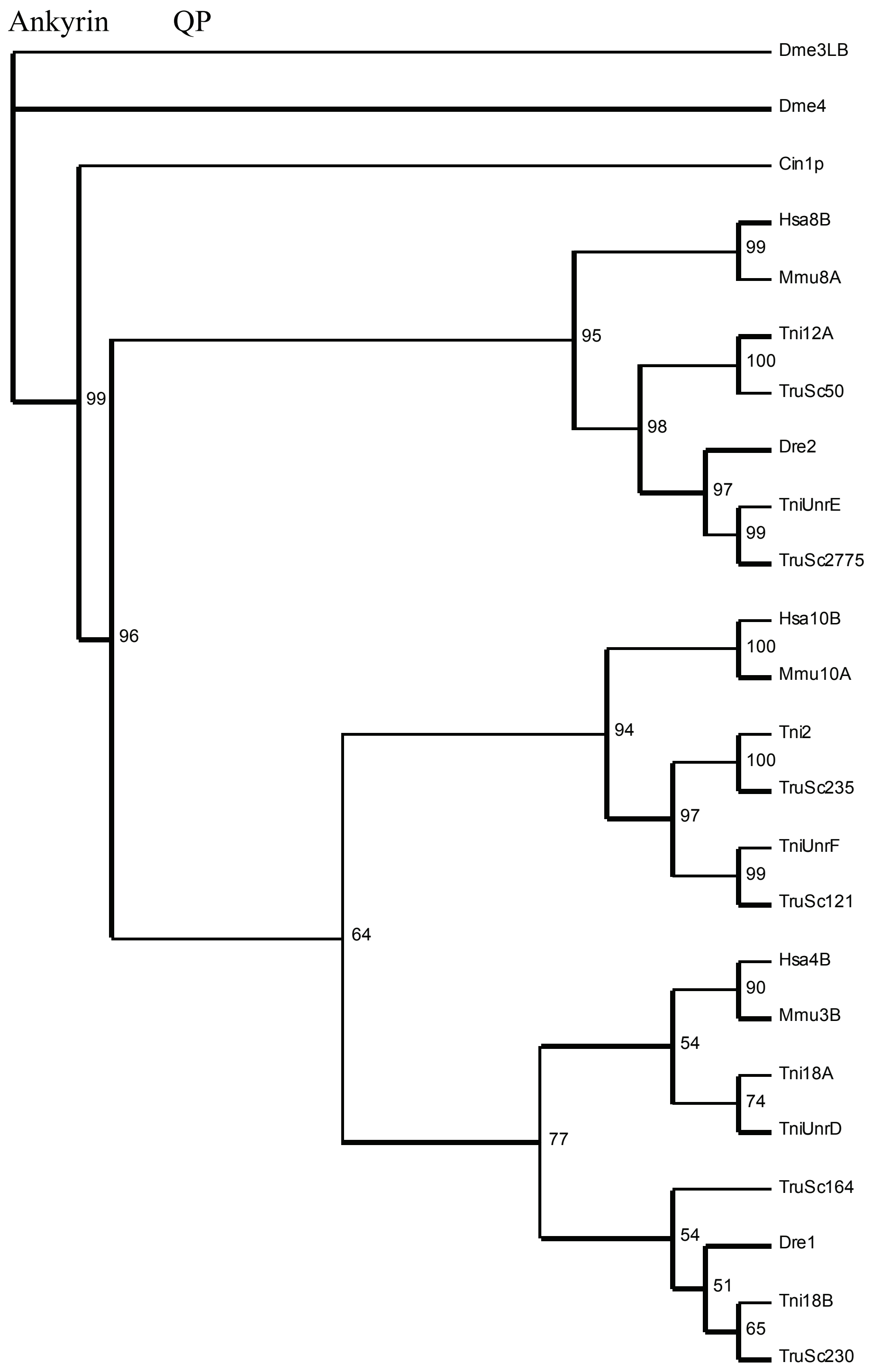

Ankyrin
QP
Dme3LB
Dme4
Cin1p
Hsa8B
99
Mmu8A
Tni12A
95
100
TruSc50
99
98
Dre2
97
TniUnrE
99
TruSc2775
Hsa10B
96
100
Mmu10A
94
Tni2
100
TruSc235
97
TniUnrF
99
TruSc121
64
Hsa4B
90
Mmu3B
54
Tni18A
74
TniUnrD
77
TruSc164
54
Dre1
51
Tni18B
65
TruSc230

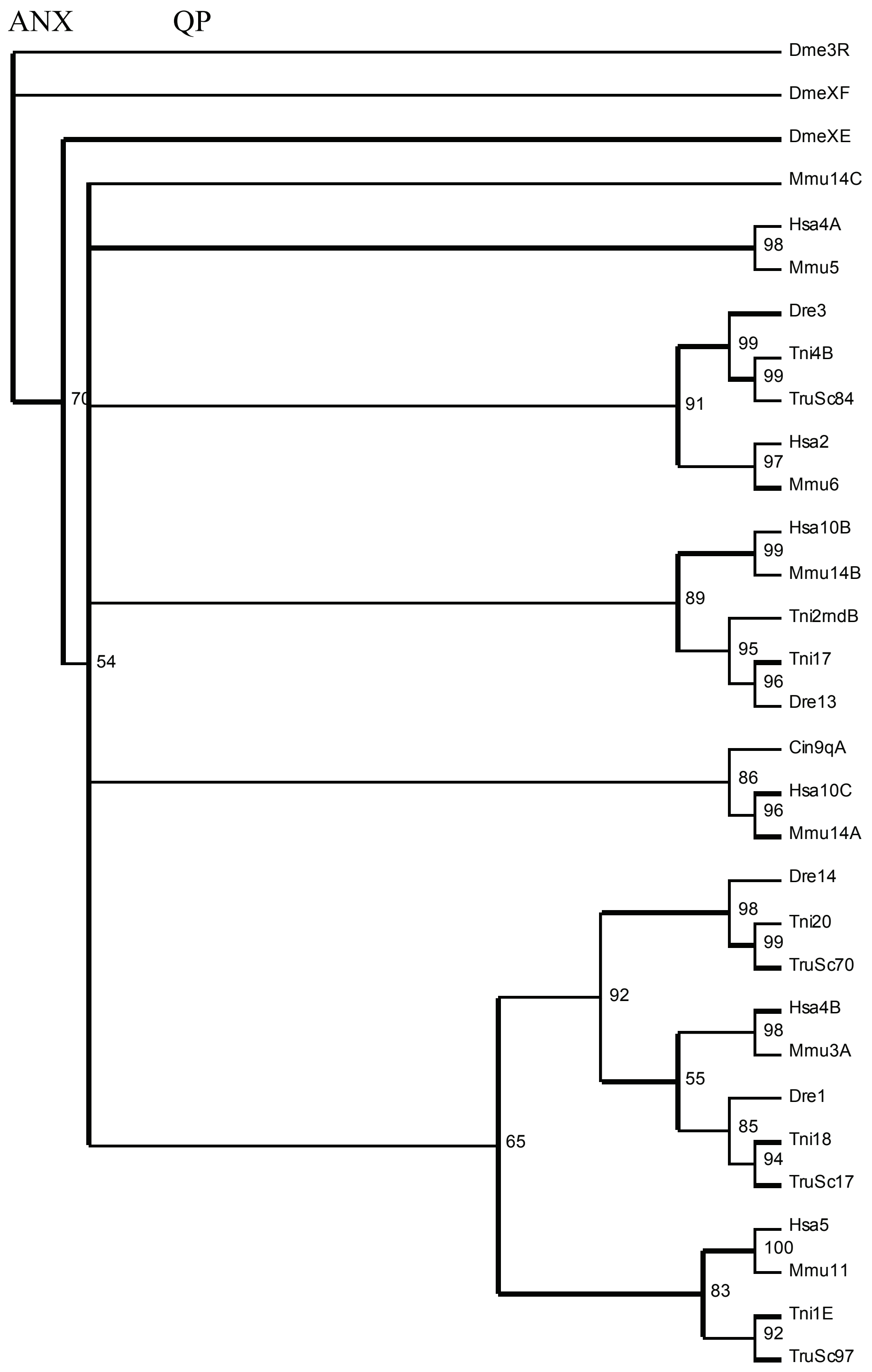
ANX
QP
Dme3R
DmeXF
DmeXE
Mmu14C
Hsa4A
98
Mmu5
Dre3
99
Tni4B
99
TruSc84
70
91
Hsa2
97
Mmu6
Hsa10B
99
Mmu14B
89
Tni2mdB
95
Tni17
54
96
Dre13
Cin9qA
86
Hsa10C
96
Mmu14A
Dre14
98
Tni20
99
TruSc70
92
Hsa4B
98
Mmu3A
55
Dre1
85
Tni18
65
94
TruSc17
Hsa5
100
Mmu11
83
Tni1E
92
TruSc97

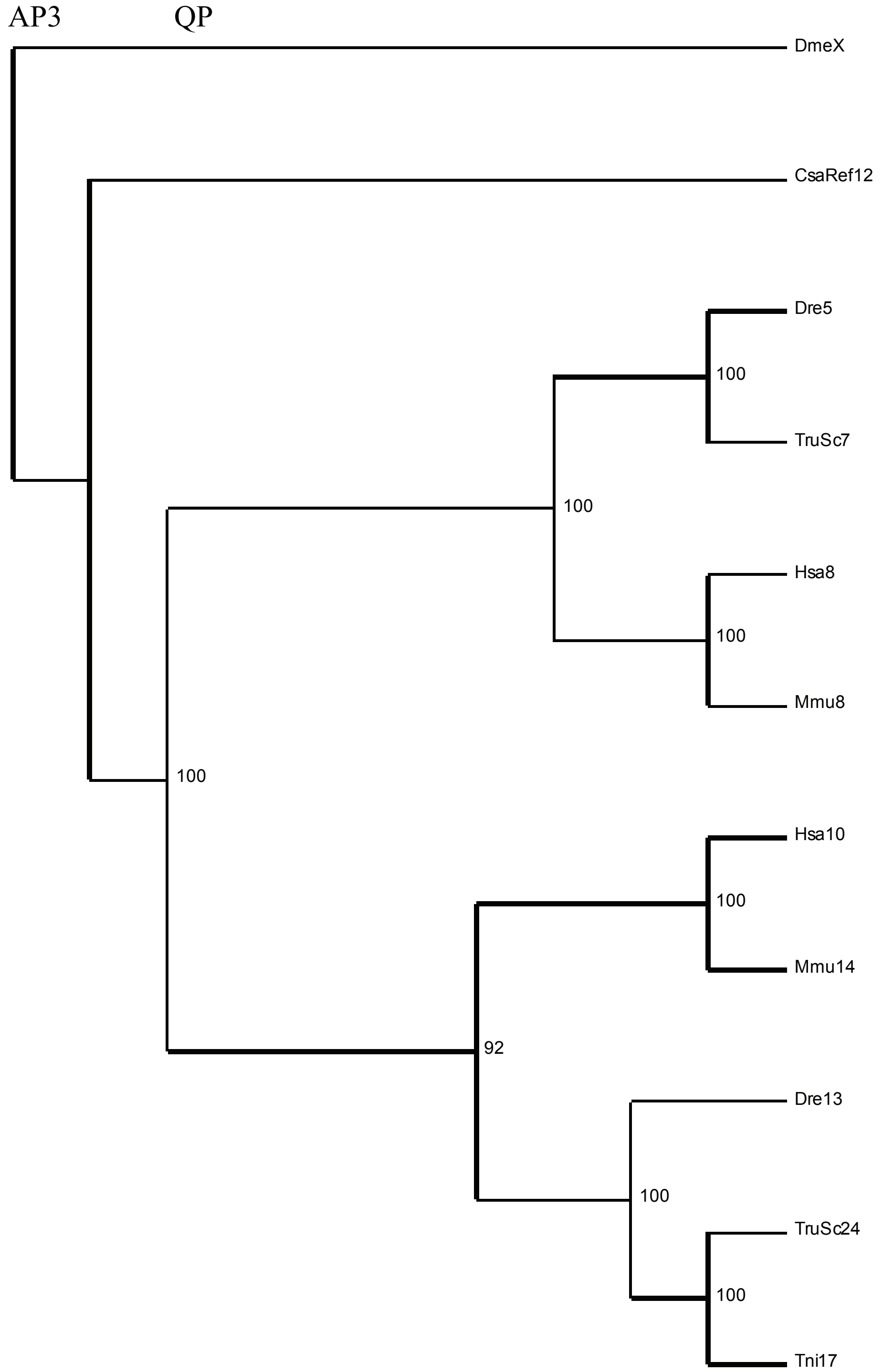
AP3
QP
DmeX
CsaRef12
Dre5
100
TruSc7
100
Hsa8
100
Mmu8
100
Hsa10
100
Mmu14
92
Dre13
100
TruSc24
100
Tni17

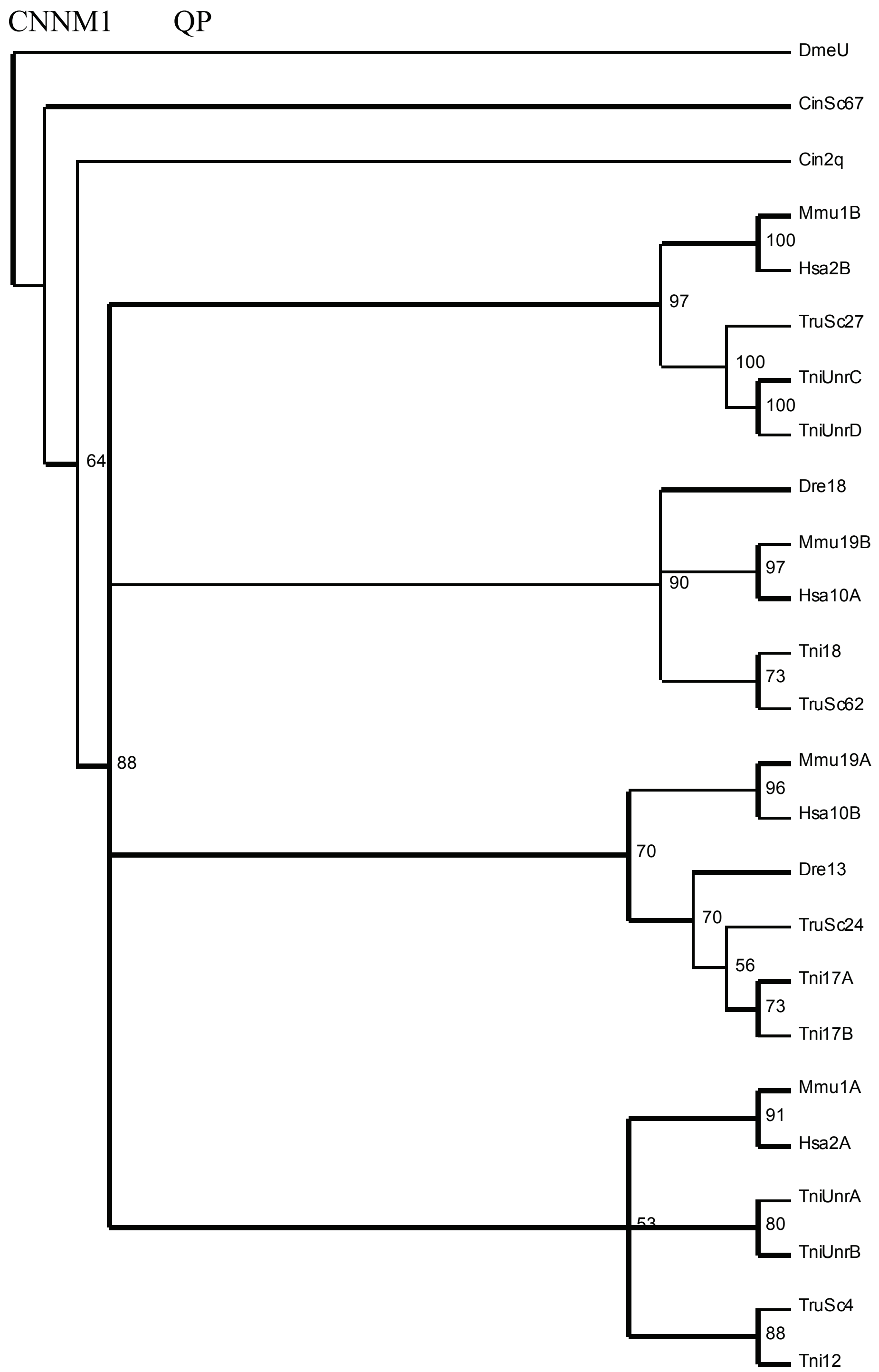
CNNM1
QP
DmeU
CinSc67
Cin2q
Mmu1B
100
Hsa2B
97
TruSc27
100
TniUnrC
100
TniUnrD
64
Dre18
Mmu19B
97
Hsa10A
90
Tni18
73
TruSc62
88
Mmu19A
96
Hsa10B
70
Dre13
70
TruSc24
56
Tni17A
73
Tni17B
Mmu1A
91
Hsa2A
TniUnrA
80
TniUnrB
TruSc4
88
Tni12

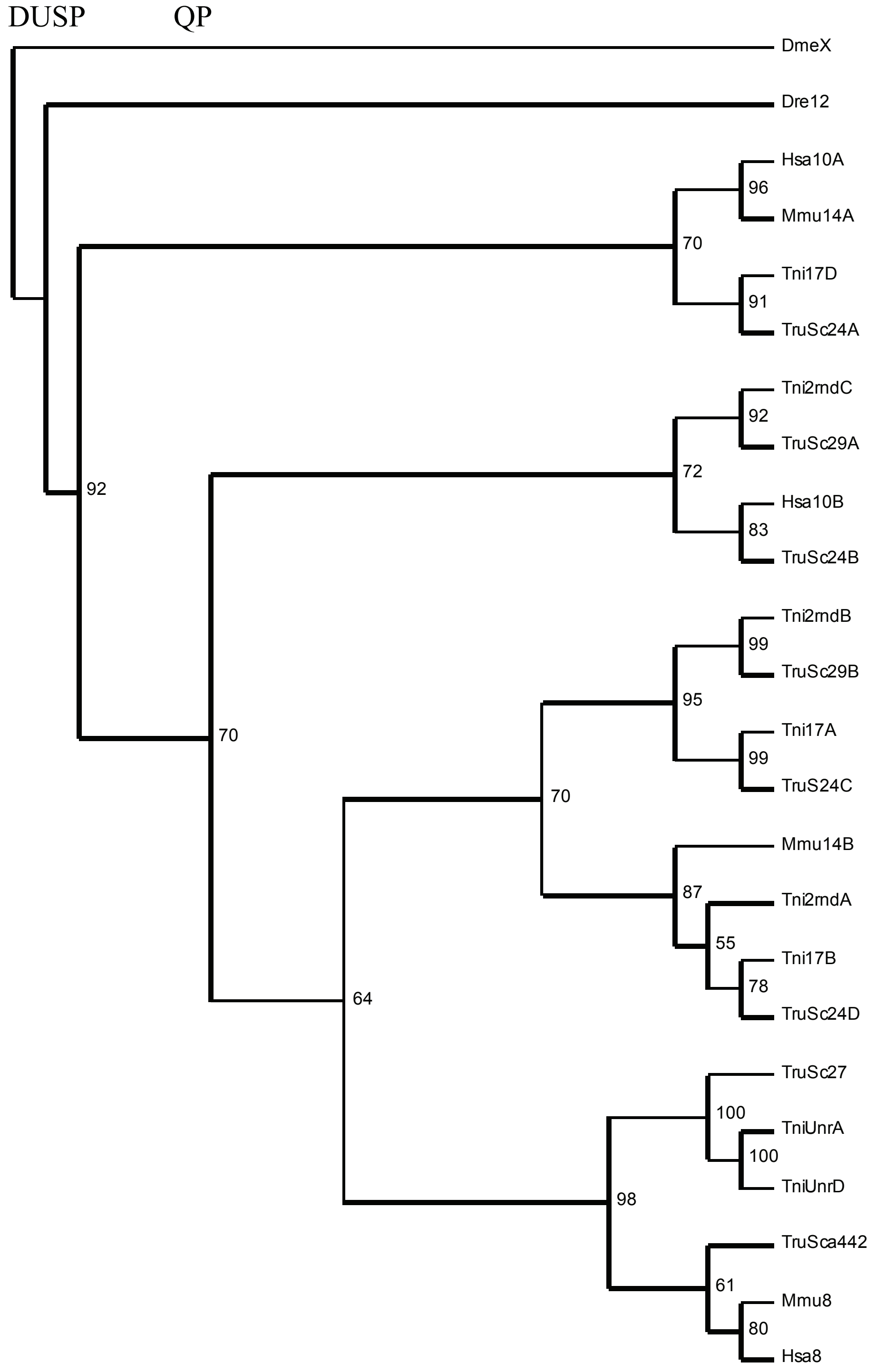
DUSP
QP
DmeX
Dre12
Hsa10A
96
Mmu14A
70
Tni17D
91
TruSc24A
Tni2mdC
92
TruSc29A
92
72
Hsa10B
83
TruSc24B
Tni2mdB
99
TruSc29B
95
Tni17A
70
99
TruS24C
70
Mmu14B
87
Tni2mdA
55
Tni17B
78
64
TruSc24D
TruSc27
100
TniUnrA
100
TniUnrD
98
TruSca442
61
Mmu8
80
Hsa8

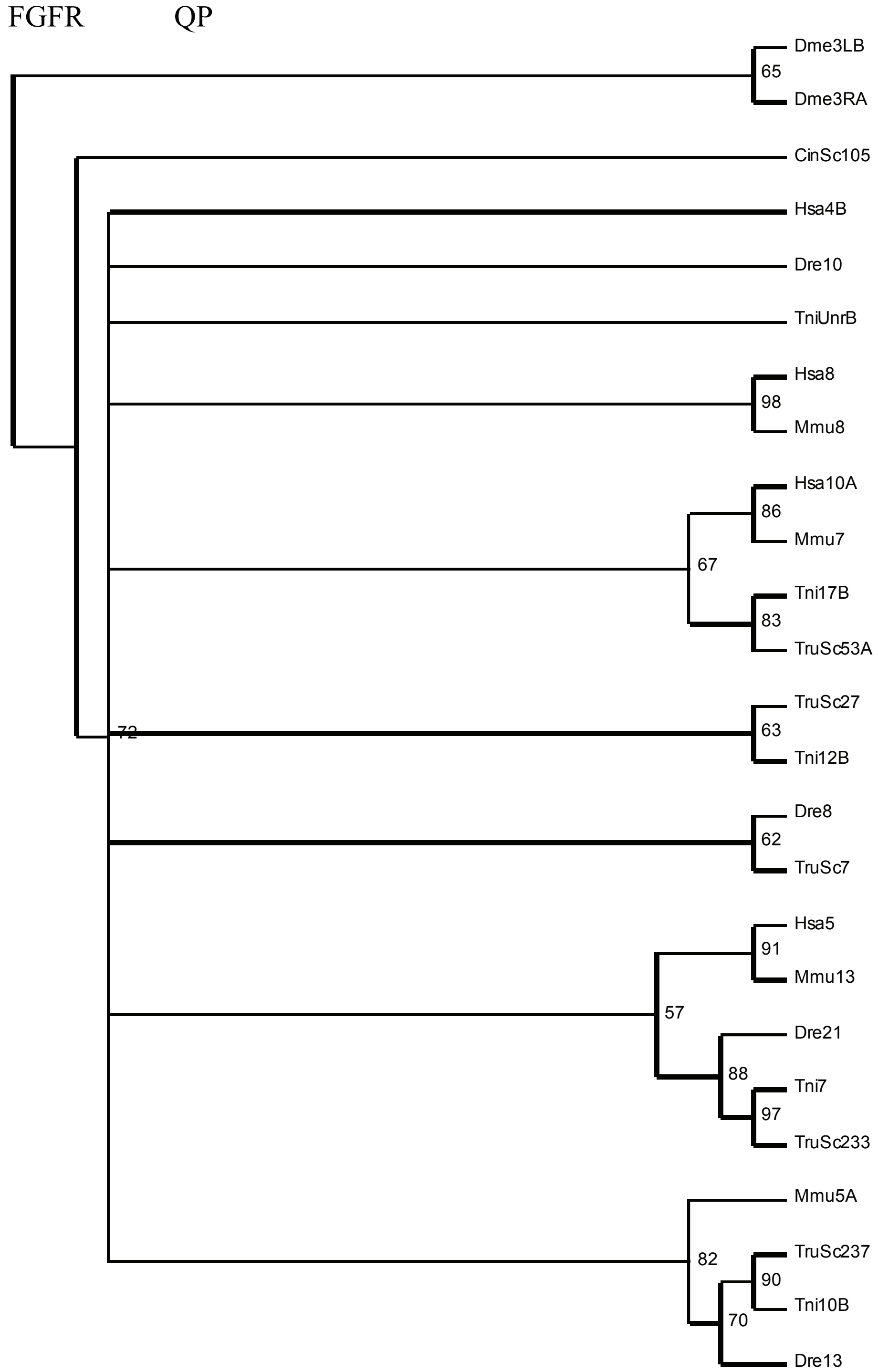
FGFR
QP
Dme3LB
65
Dme3RA
CinSc105
Hsa4B
Dre10
TniUnrB
Hsa8
98
Mmu8
Hsa10A
86
Mmu7
67
Tni17B
83
TruSc53A
TruSc27
72
63
Tni12B
Dre8
62
TruSc7
Hsa5
91
Mmu13
57
Dre21
88
Tni7
97
TruSc233
Mmu5A
TruSc237
82
90
Tni10B
70
Dre13

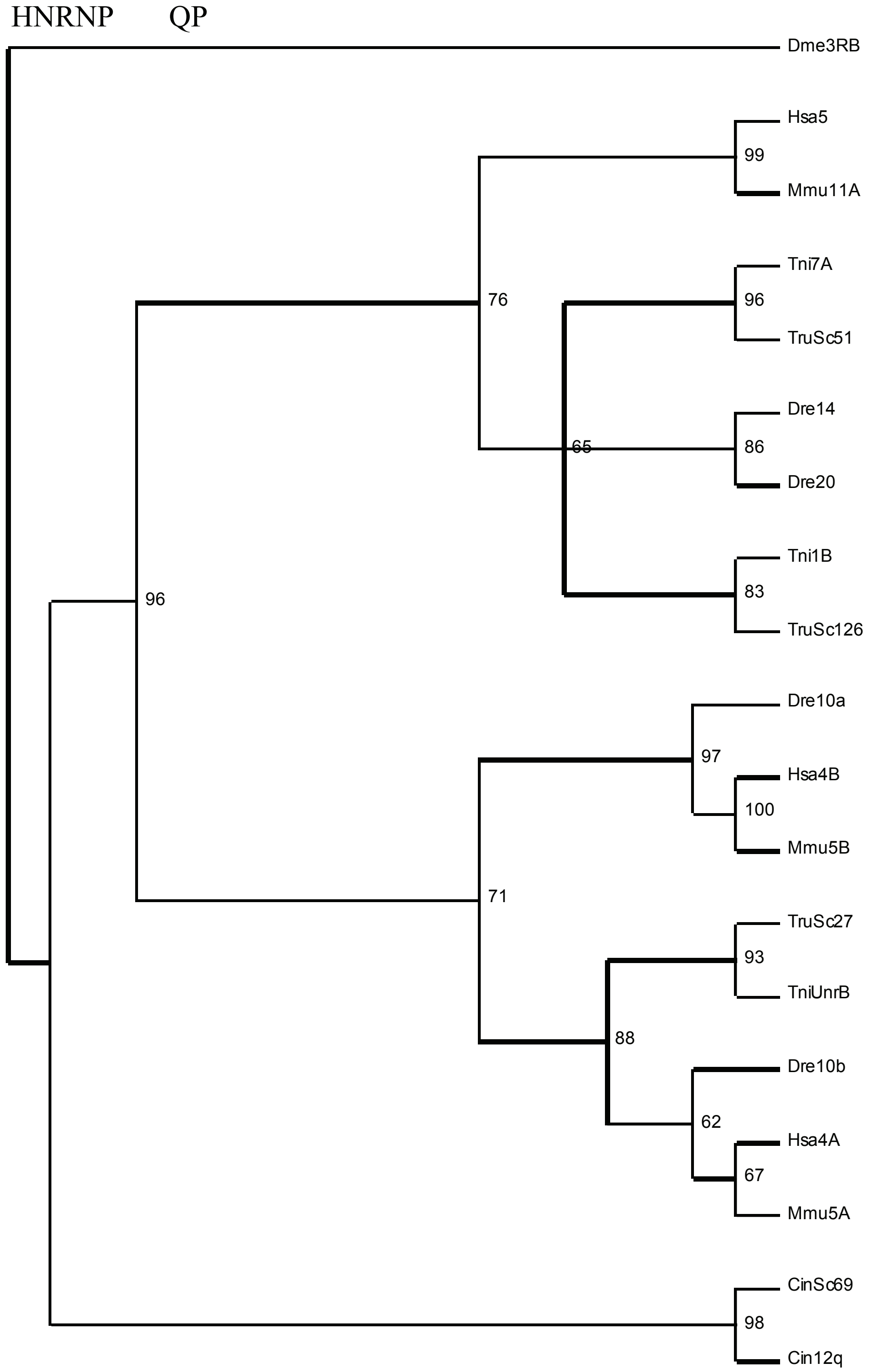
HNRNP
QP
Dme3RB
Hsa5
99
Mmu11A
Tni7A
76
96
TruSc51
Dre14
65
86
Dre20
Tni1B
96
83
TruSc126
Dre10a
97
Hsa4B
100
Mmu5B
71
TruSc27
93
TniUnrB
88
Dre10b
62
Hsa4A
67
Mmu5A
CinSc69
98
Cin12q

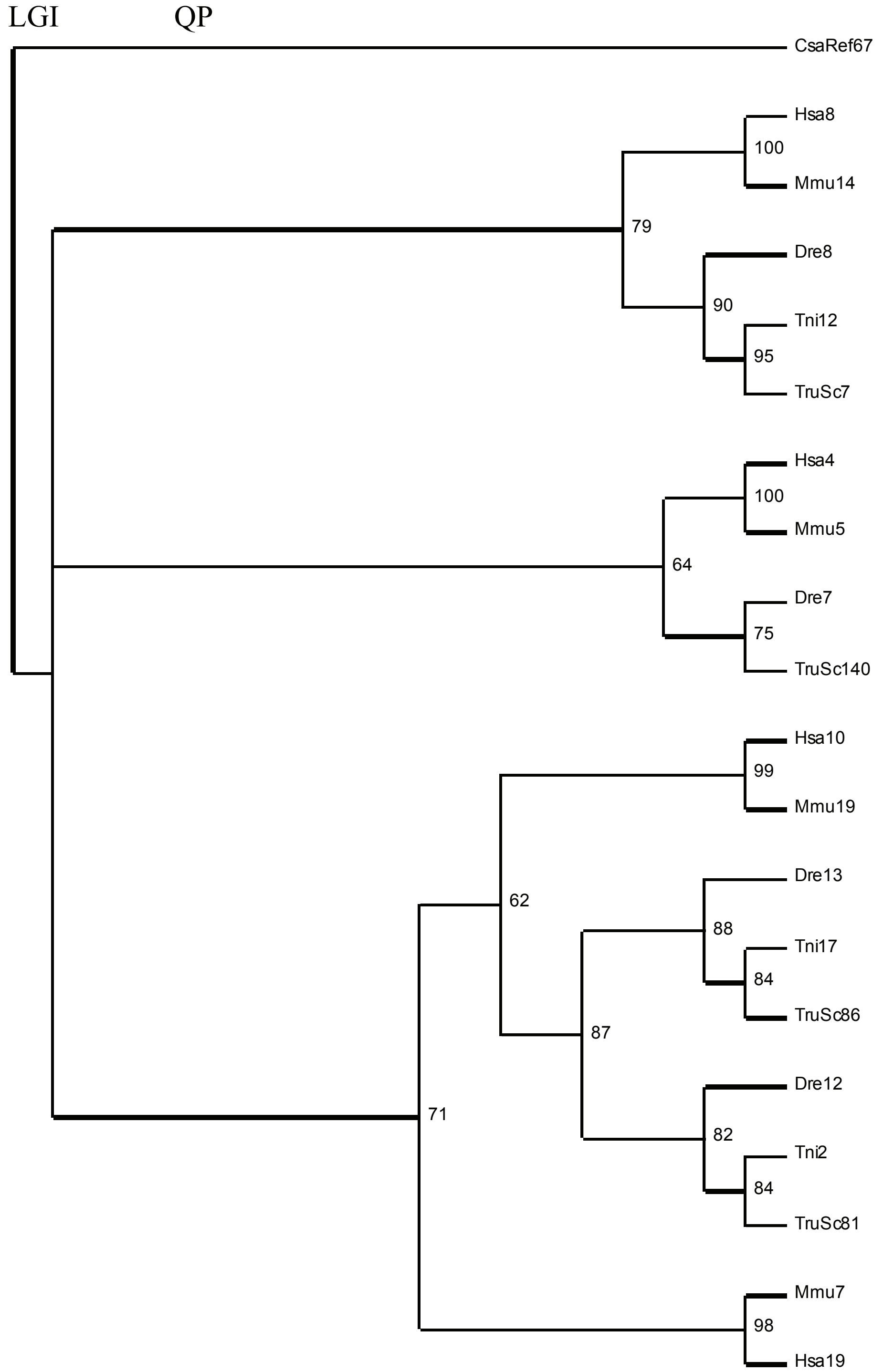

LGI
QP
CsaRef67
Hsa8
100
Mmu14
79
Dre8
90
Tni12
95
TruSc7
Hsa4
100
Mmu5
64
Dre7
75
TruSc140
Hsa10
99
Mmu19
62
Dre13
88
Tni17
84
TruSc86
87
Dre12
82
Tni2
84
TruSc81
71
Mmu7
98
Hsa19

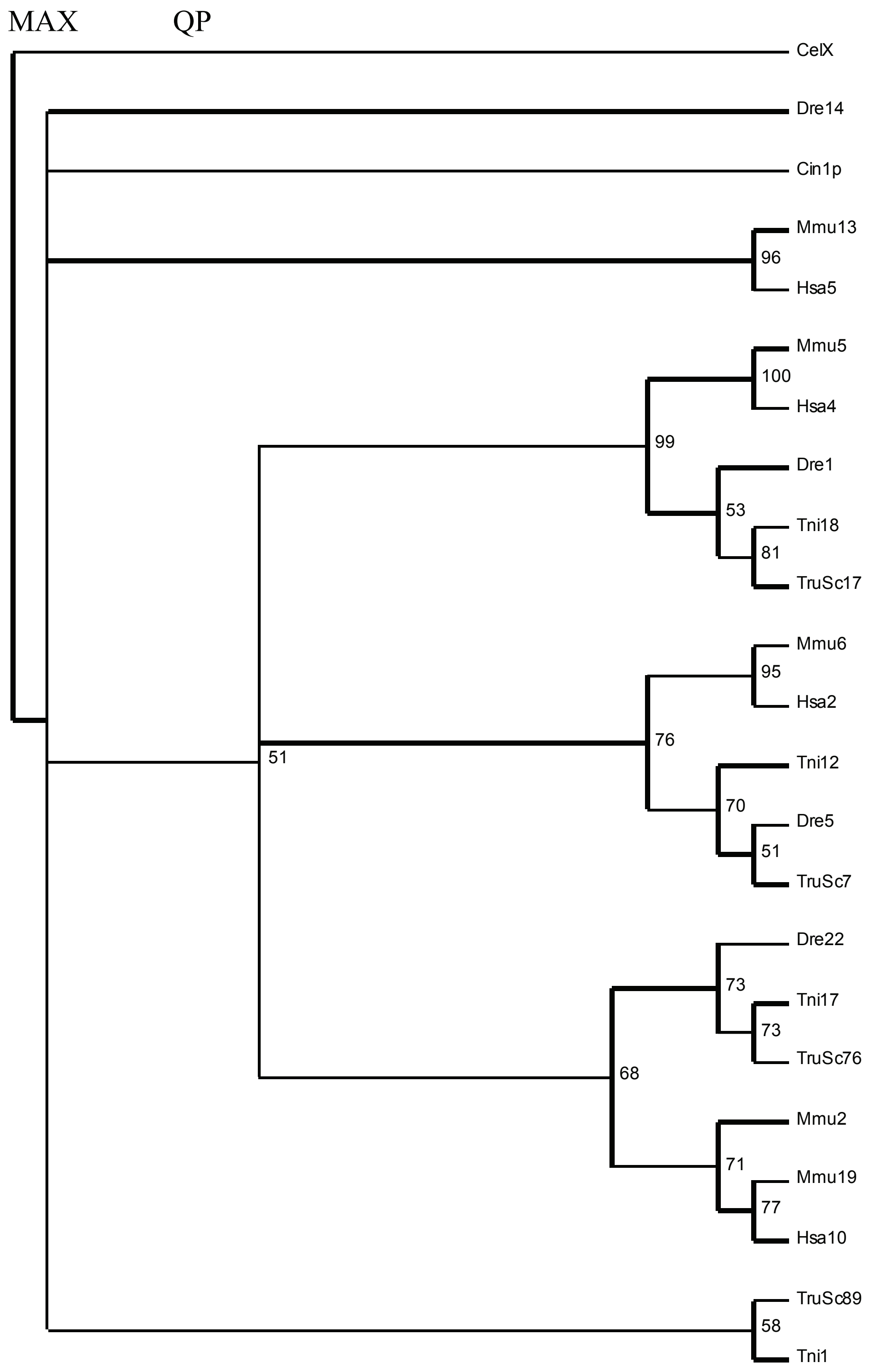
MAX
QP
CelX
Dre14
Cin1p
Mmu13
96
Hsa5
Mmu5
100
Hsa4
99
Dre1
53
Tni18
81
TruSc17
Mmu6
95
Hsa2
76
51
Tni12
70
Dre5
51
TruSc7
Dre22
73
Tni17
73
TruSc76
68
Mmu2
71
Mmu19
77
Hsa10
TruSc89
58
Tni1

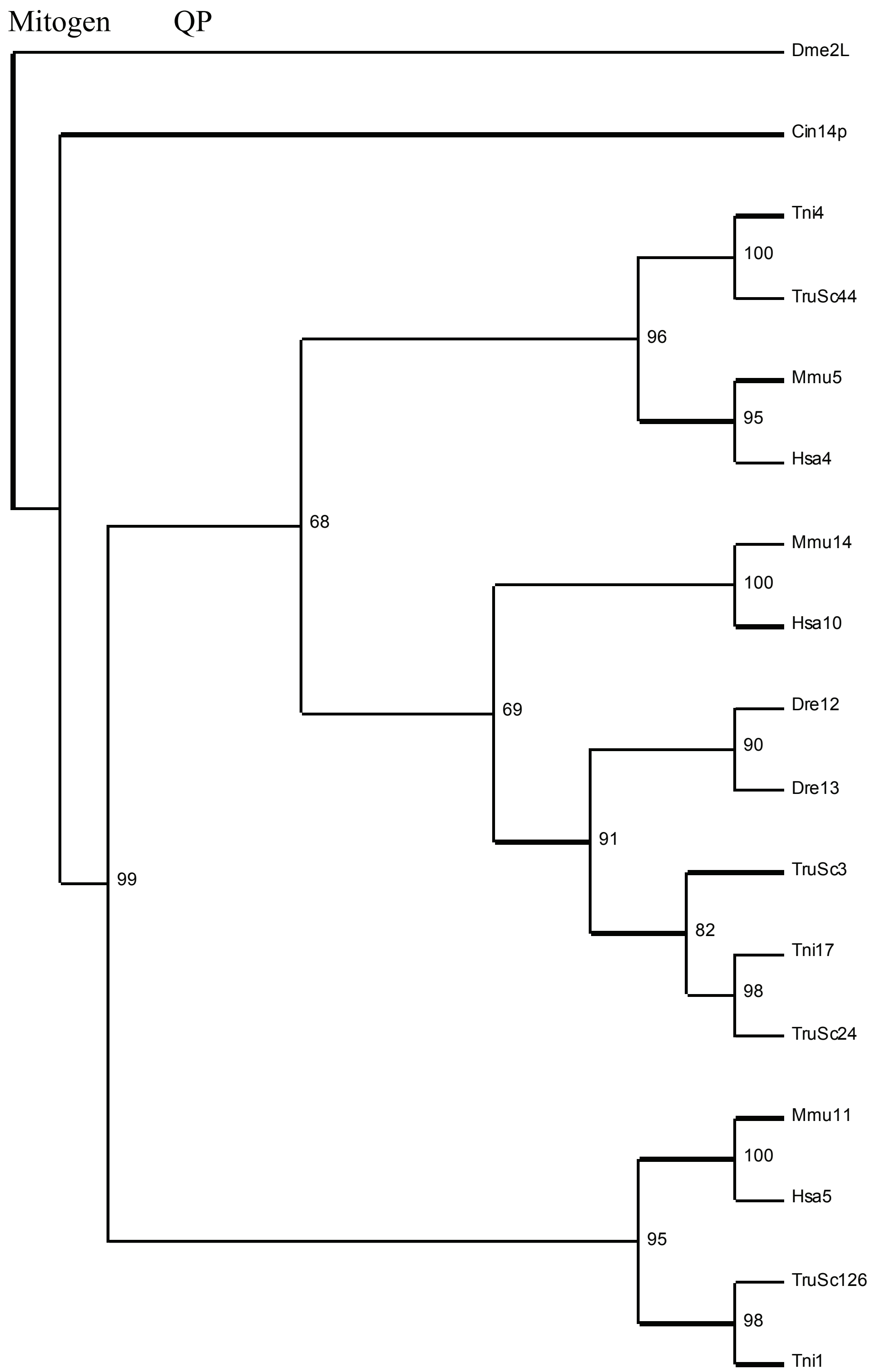
Mitogen
QP
Dme2L
Cin14p
Tni4
100
TruSc44
96
Mmu5
95
Hsa4
68
Mmu14
100
Hsa10
Dre12
69
90
Dre13
91
TruSc3
99
82
Tni17
98
TruSc24
Mmu11
100
Hsa5
95
TruSc126
98
Tni1

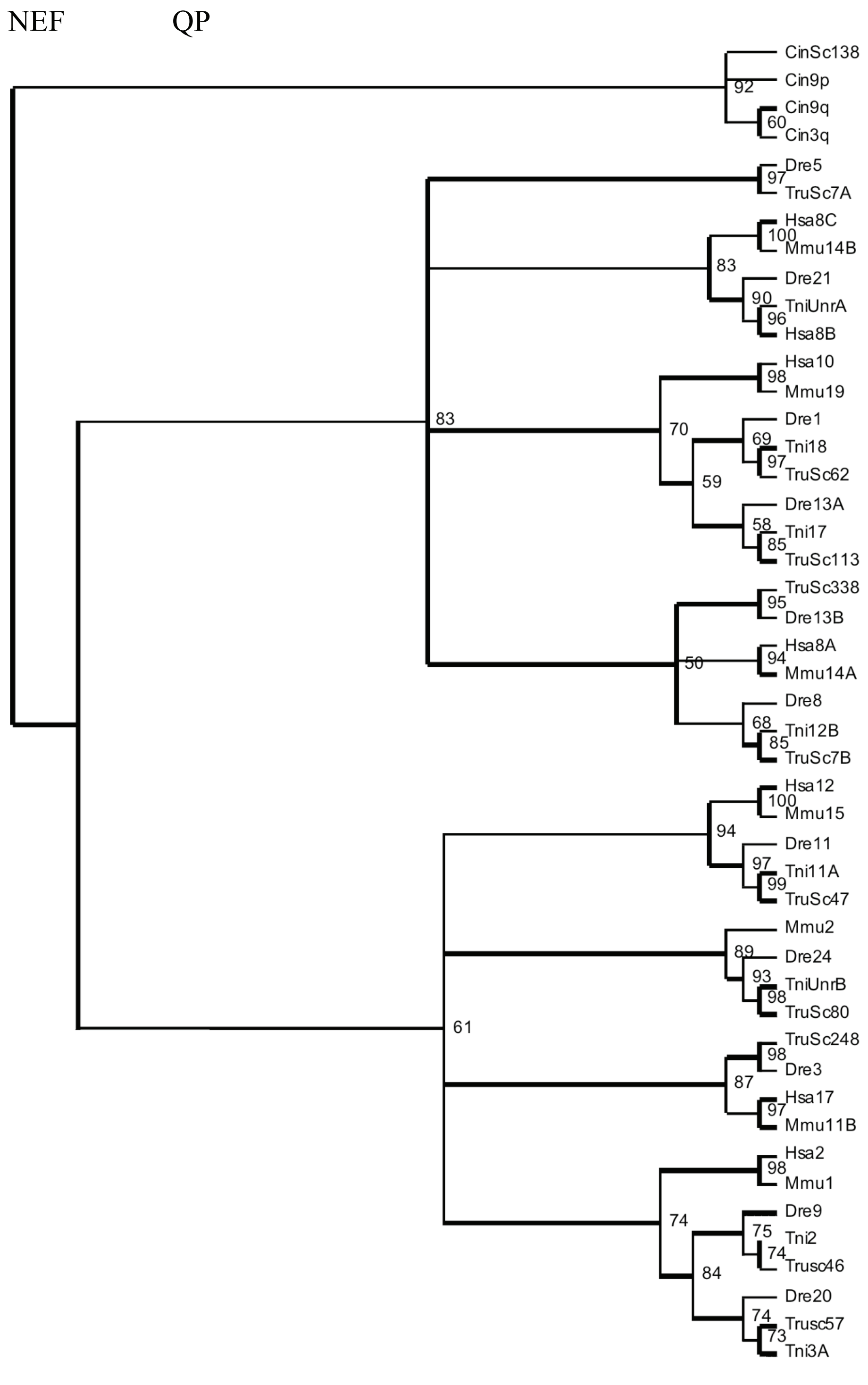
NEF
QP
CinSc138
Cin9p
92
Cin9q
60
Cin3q
Dre5
97
TruSc7A
Hsa8C
100
Mmu14B
83
Dre21
90
TniUnrA
96
Hsa8B
Hsa10
98
Mmu19
83
70
Dre1
69
Tni18
97
TruSc62
59
Dre13A
58
Tni17
85
TruSc113
TruSc338
95
Dre13B
Hsa8A
94
50
Mmu14A
Dre8
68
Tni12B
85
TruSc7B
Hsa12
100
Mmu15
94
Dre11
97
Tni11A
99
TruSc47
Mmu2
89
Dre24
93
TniUnrB
98
TruSc80
61
TruSc248
98
Dre3
87
Hsa17
97
Mmu11B
Hsa2
98
Mmu1
Dre9
74
75
Tni2
74
Trusc46
84
Dre20
74
Trusc57
73
Tni3A

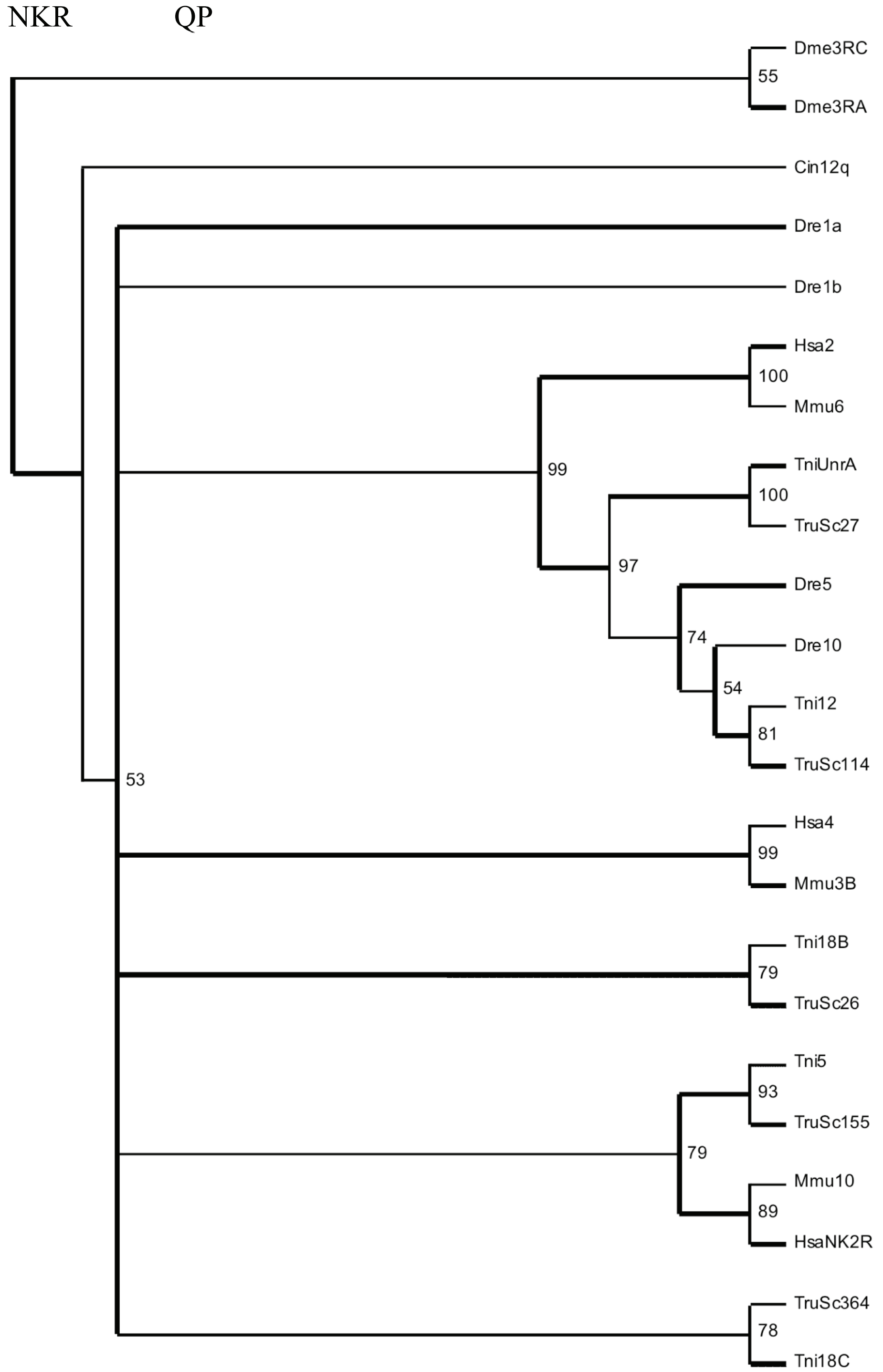

NKR
QP
Dme3RC
55
Dme3RA
Cin12q
Dre1a
Dre1b
Hsa2
100
Mmu6
99
TniUnrA
100
TruSc27
97
Dre5
74
Dre10
54
Tni12
81
TruSc114
53
Hsa4
99
Mmu3B
Tni18B
79
TruSc26
Tni5
93
TruSc155
79
Mmu10
89
HsaNK2R
TruSc364
78
Tni18C

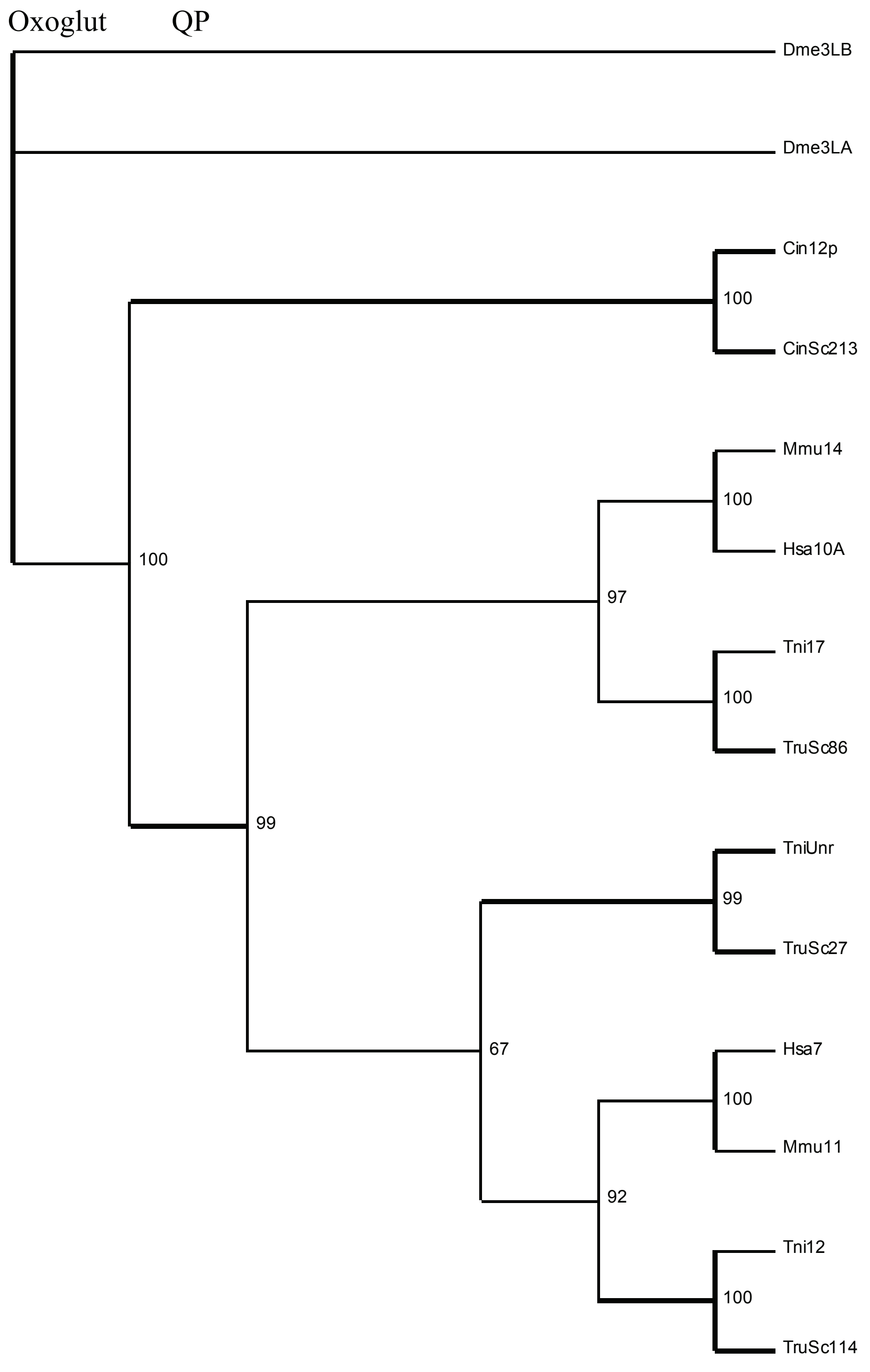
Oxoglut QP
Dme3LB
Dme3LA
Cin12p
100
CinSc213
Mmu14
100
Hsa10A
100
97
Tni17
100
TruSc86
99
TniUnr
99
TruSc27
67
Hsa7
100
Mmu11
92
Tni12
100
TruSc114

PDLIM3 QP

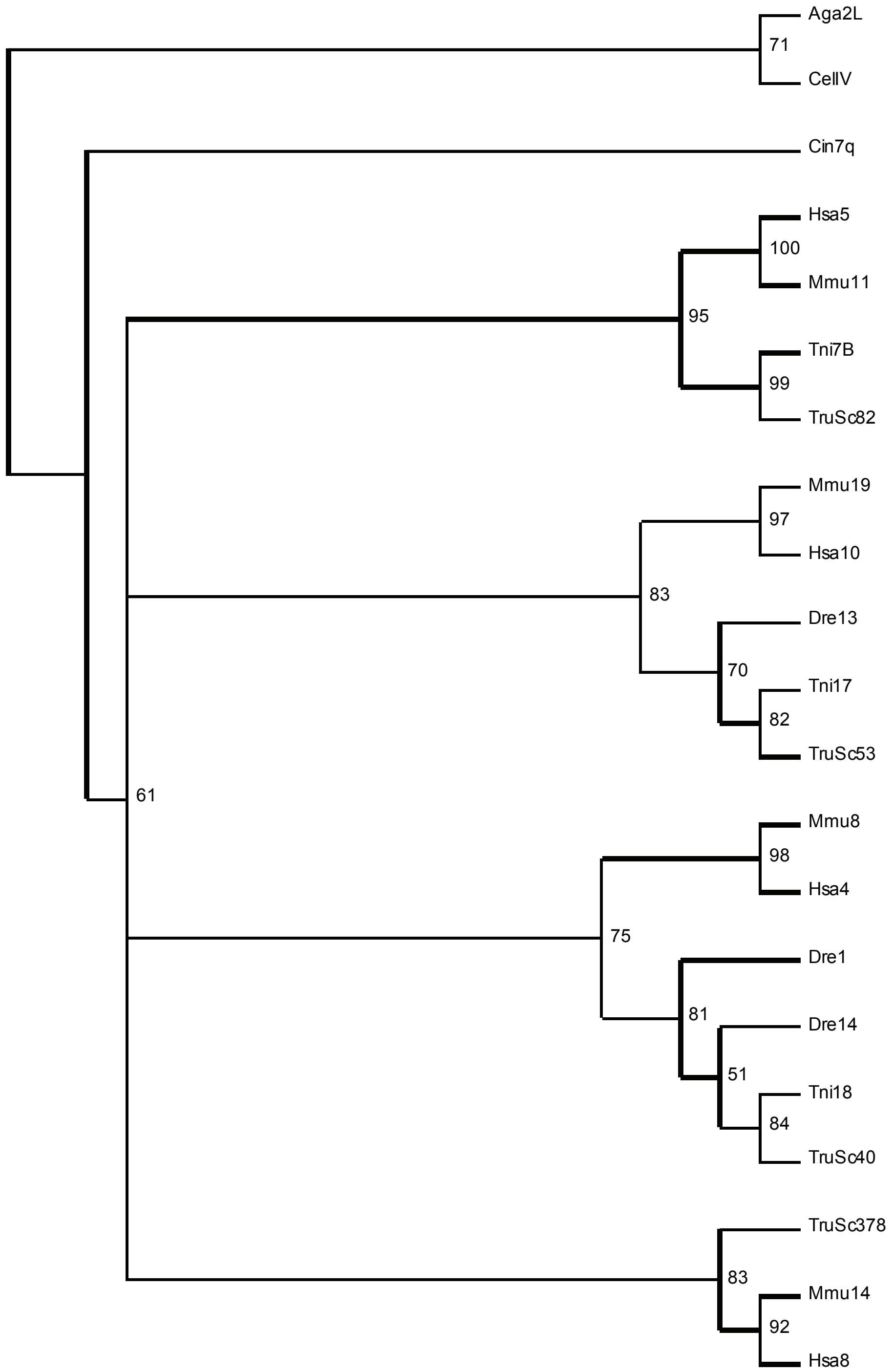

Aga2L
71
CellV
Cin7q
Hsa5
100
Mmu11
95
Tni7B
99
TruSc82
Mmu19
97
Hsa10
83
Dre13
70
Tni17
82
TruSc53
61
Mmu8
98
Hsa4
75
Dre1
81
Dre14
51
Tni18
84
TruSc40
TruSc378
83
Mmu14
92
Hsa8

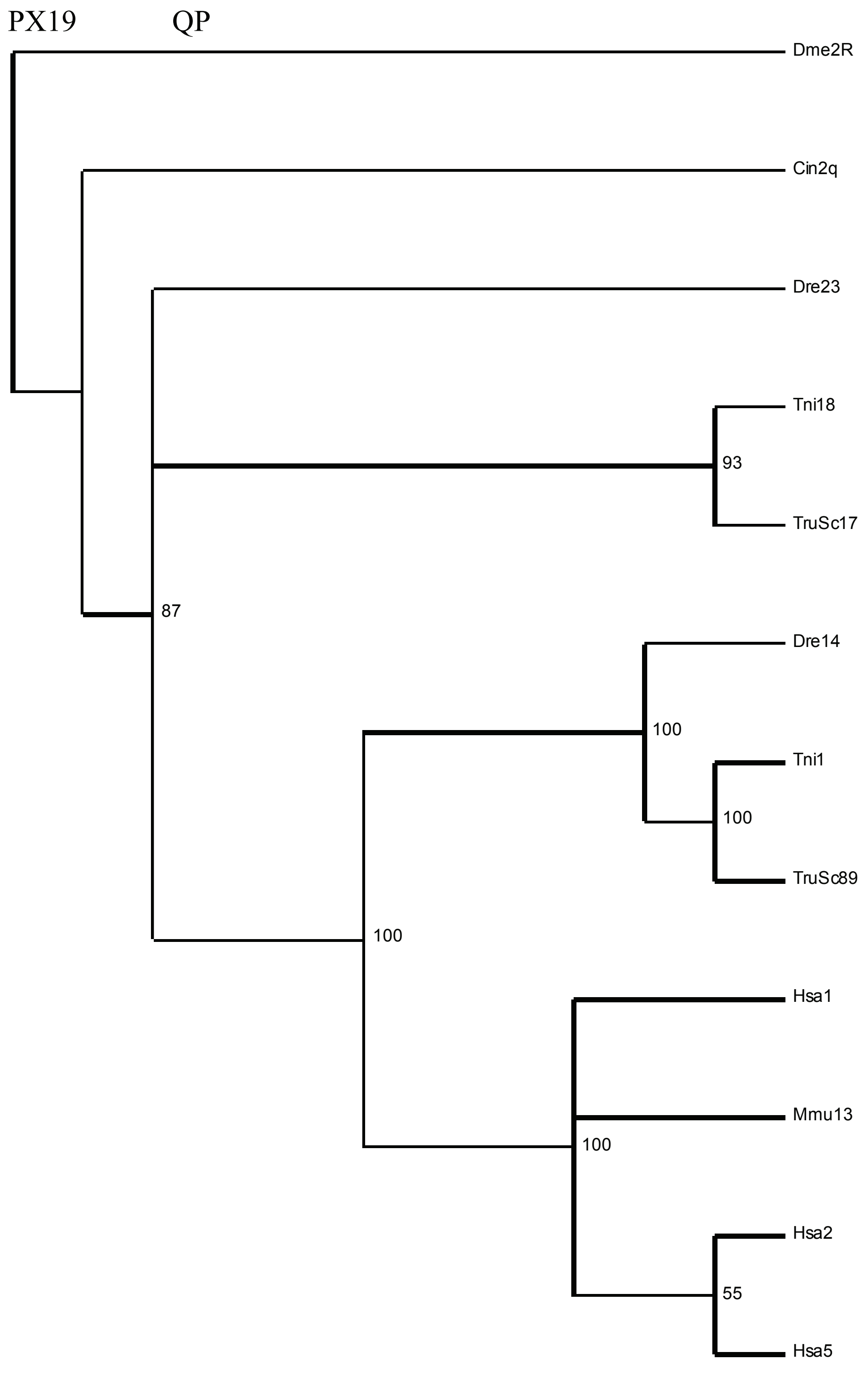
PX19
QP
Dme2R
Cin2q
Dre23
Tni18
93
TruSc17
87
Dre14
100
Tni1
100
TruSc89
100
Hsa1
Mmu13
100
Hsa2
55
Hsa5

SAMD8Sub1 QP

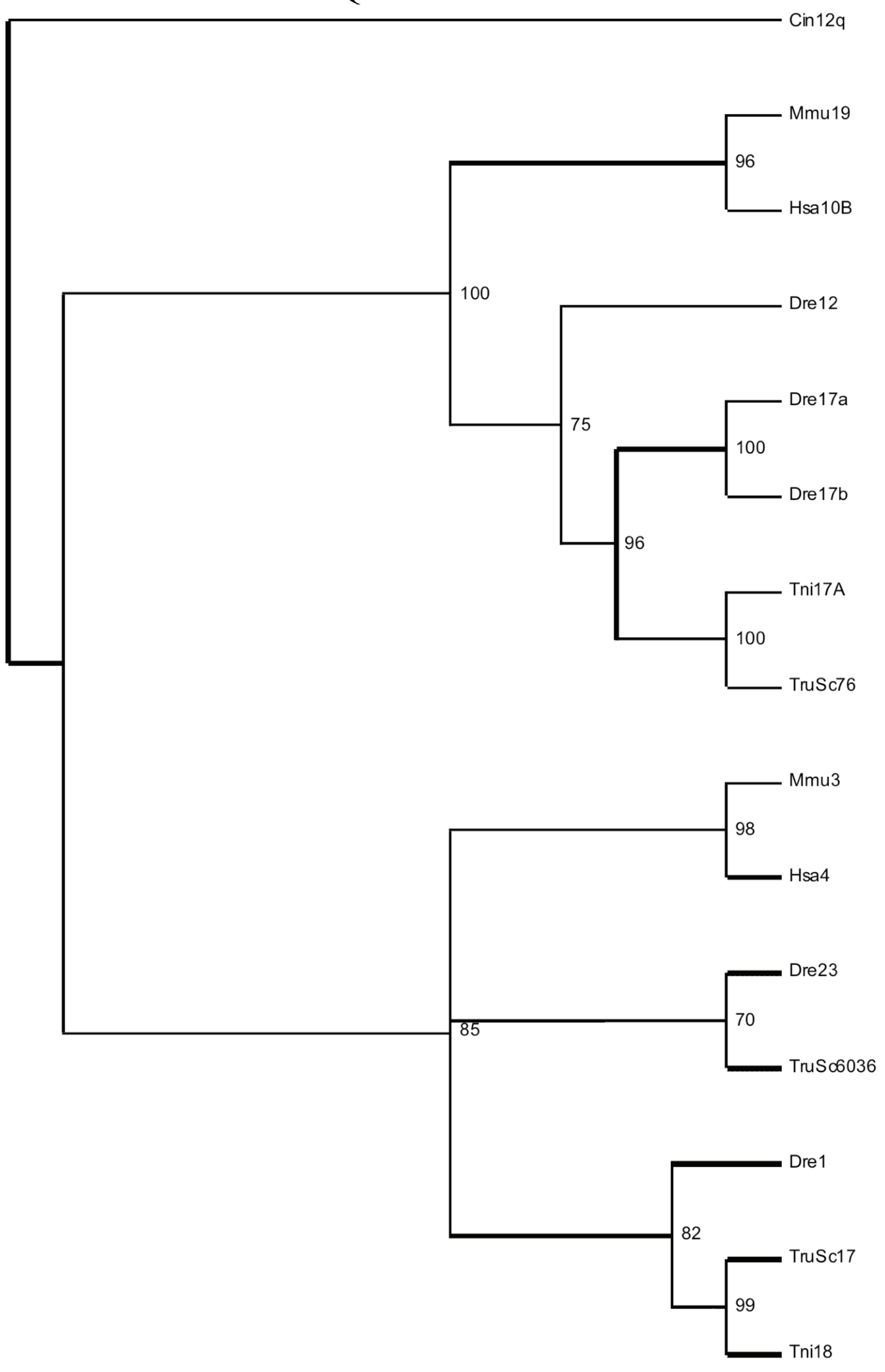

SAMD8Sub2 QP

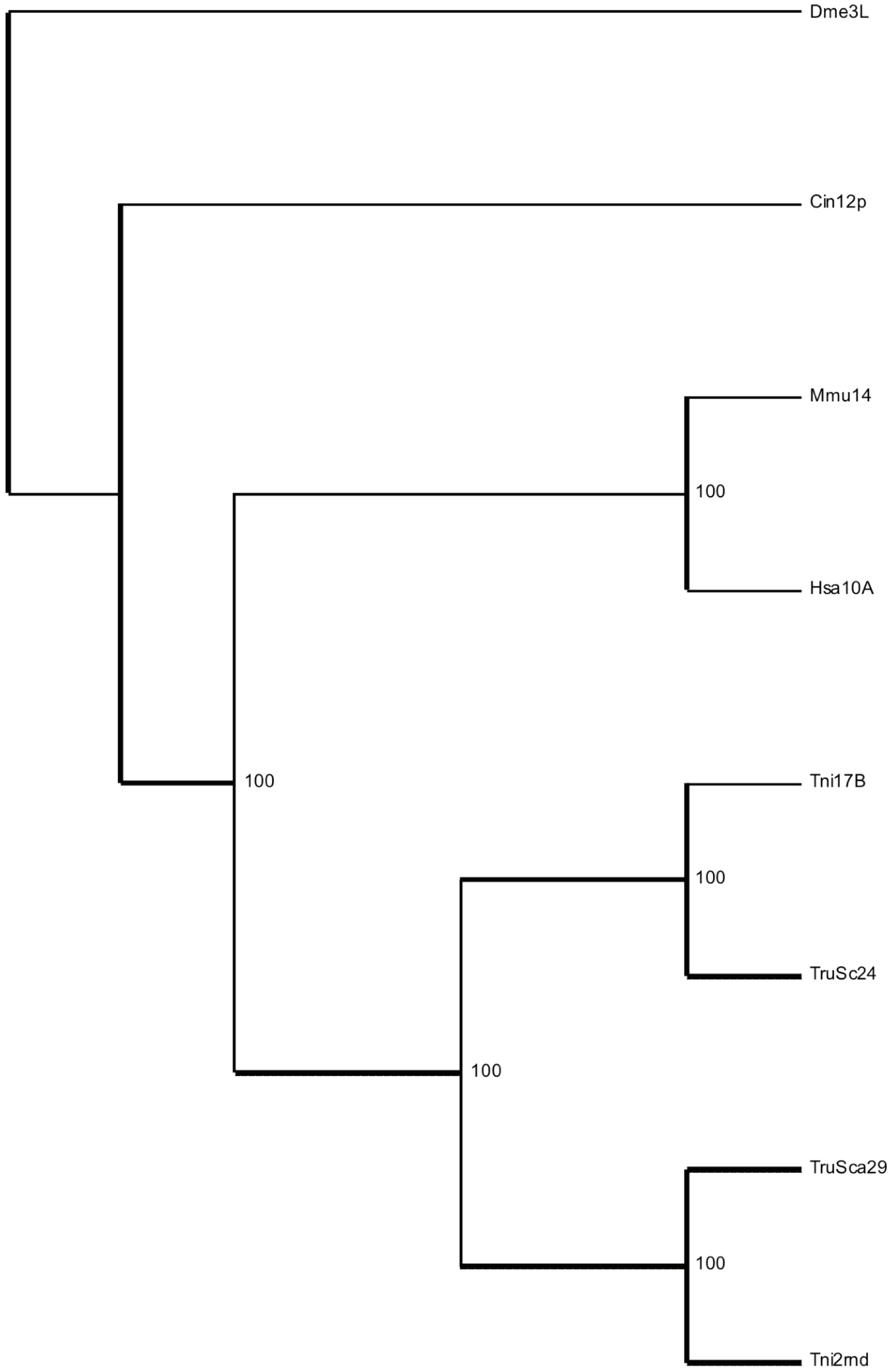

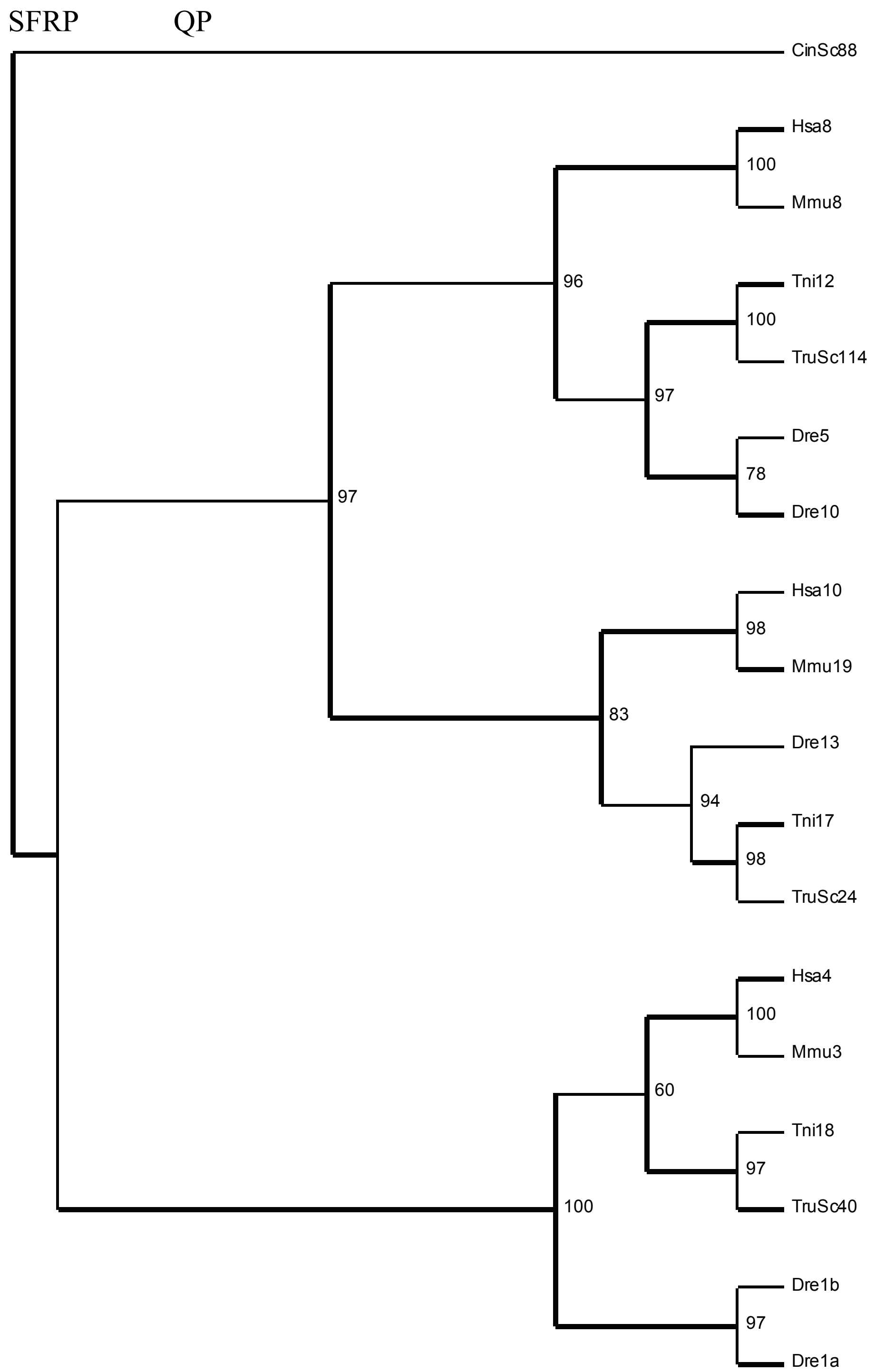

SFRP
QP
CinSc88
Hsa8
100
Mmu8
96
Tni12
100
TruSc114
97
Dre5
78
Dre10
97
Hsa10
98
Mmu19
83
Dre13
94
Tni17
98
TruSc24
Hsa4
100
Mmu3
60
Tni18
97
TruSc40
100
Dre1b
97
Dre1a

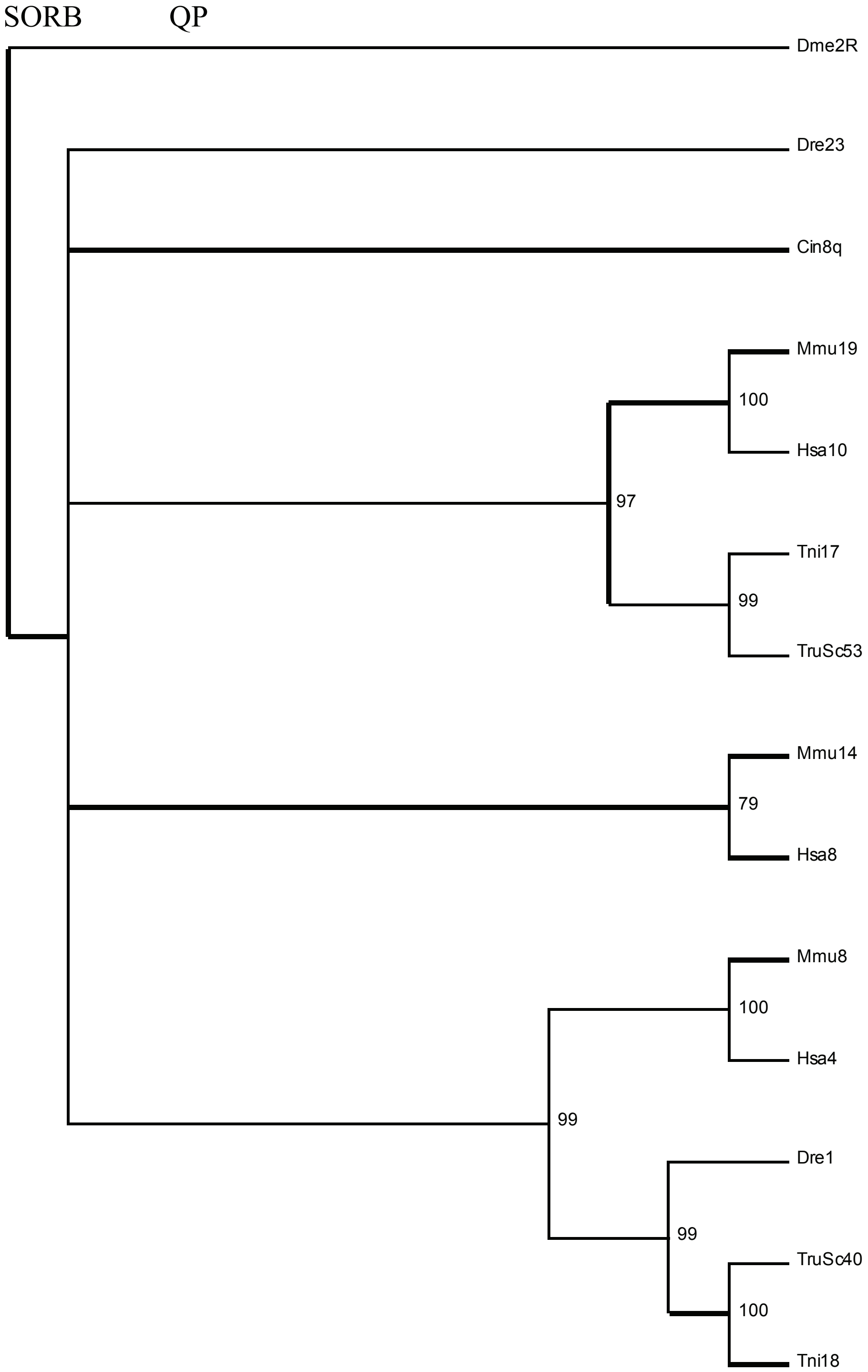
SORB
QP
Dme2R
Dre23
Cin8q
Mmu19
100
Hsa10
97
Tni17
99
TruSc53
Mmu14
79
Hsa8
Mmu8
100
Hsa4
99
Dre1
99
TruSc40
100
Tni18

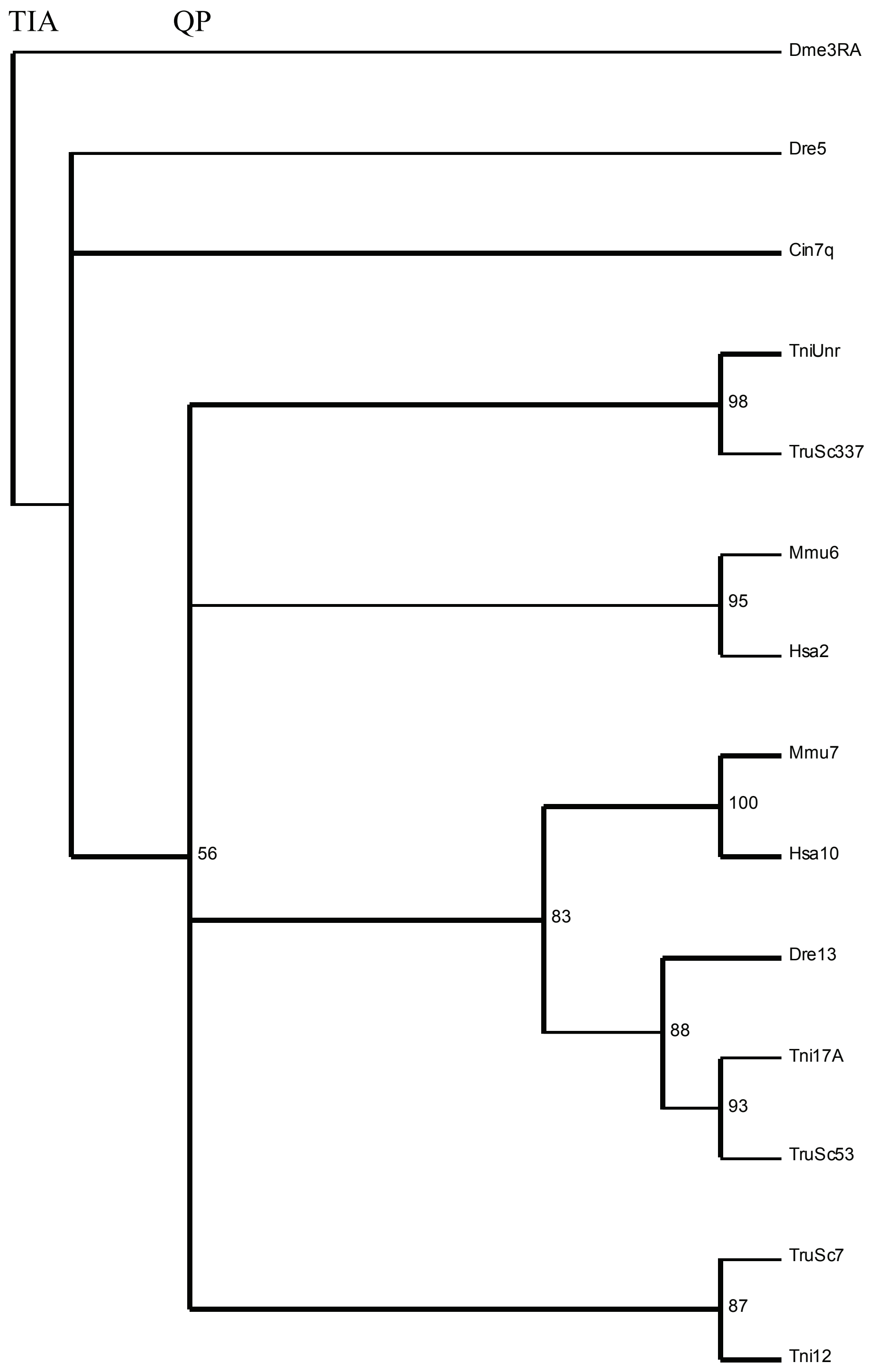
TIA
QP
Dme3RA
Dre5
Cin7q
TniUnr
98
TruSc337
Mmu6
95
Hsa2
Mmu7
100
Hsa10
56
83
Dre13
88
Tni17A
93
TruSc53
TruSc7
87
Tni12

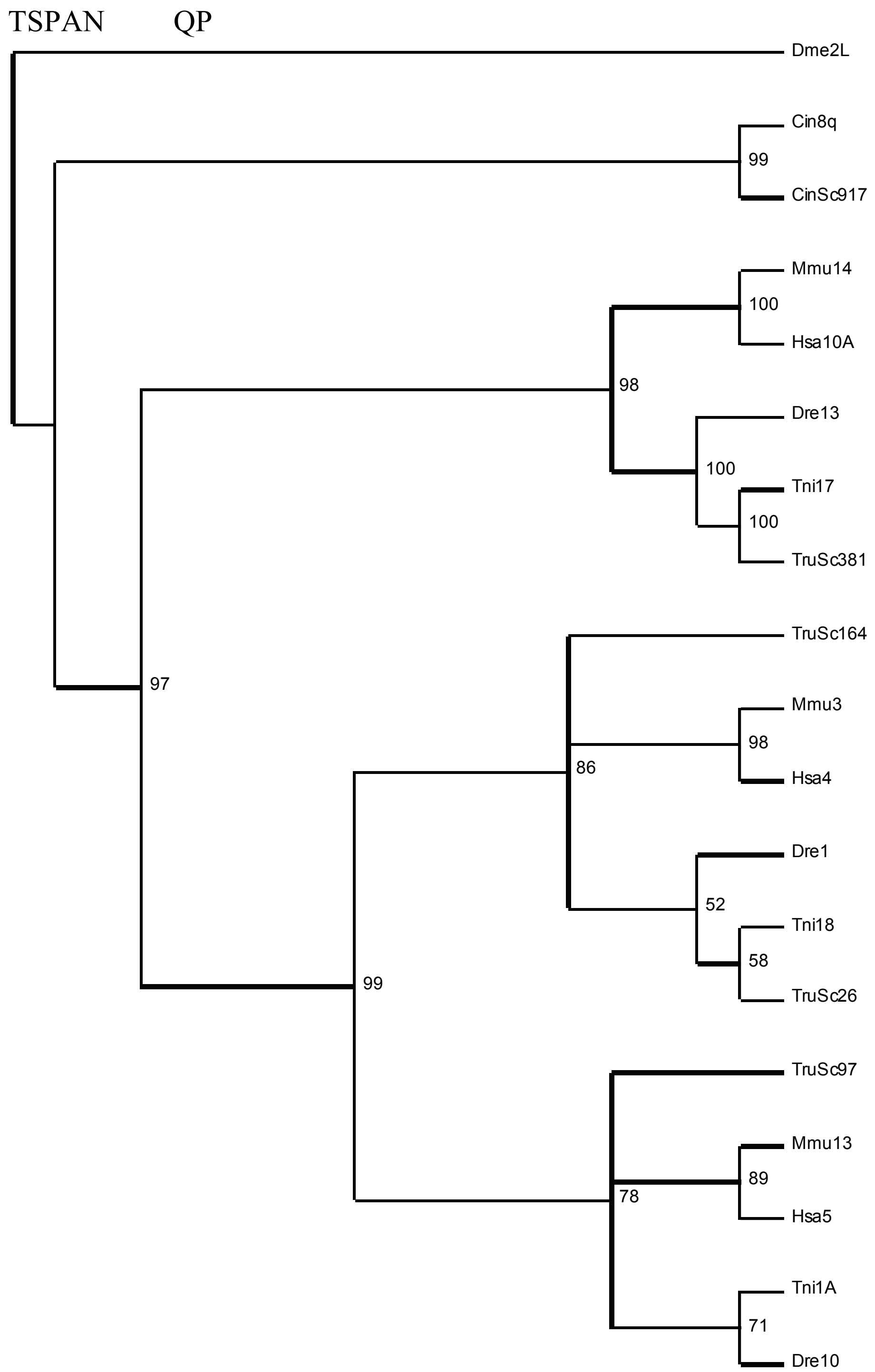
TSPAN
QP
Dme2L
Cin8q
99
CinSc917
Mmu14
100
Hsa10A
98
Dre13
100
Tni17
100
TruSc381
97
TruSc164
Mmu3
98
86
Hsa4
Dre1
52
Tni18
58
TruSc26
99
TruSc97
Mmu13
89
78
Hsa5
Tni1A
71
Dre10

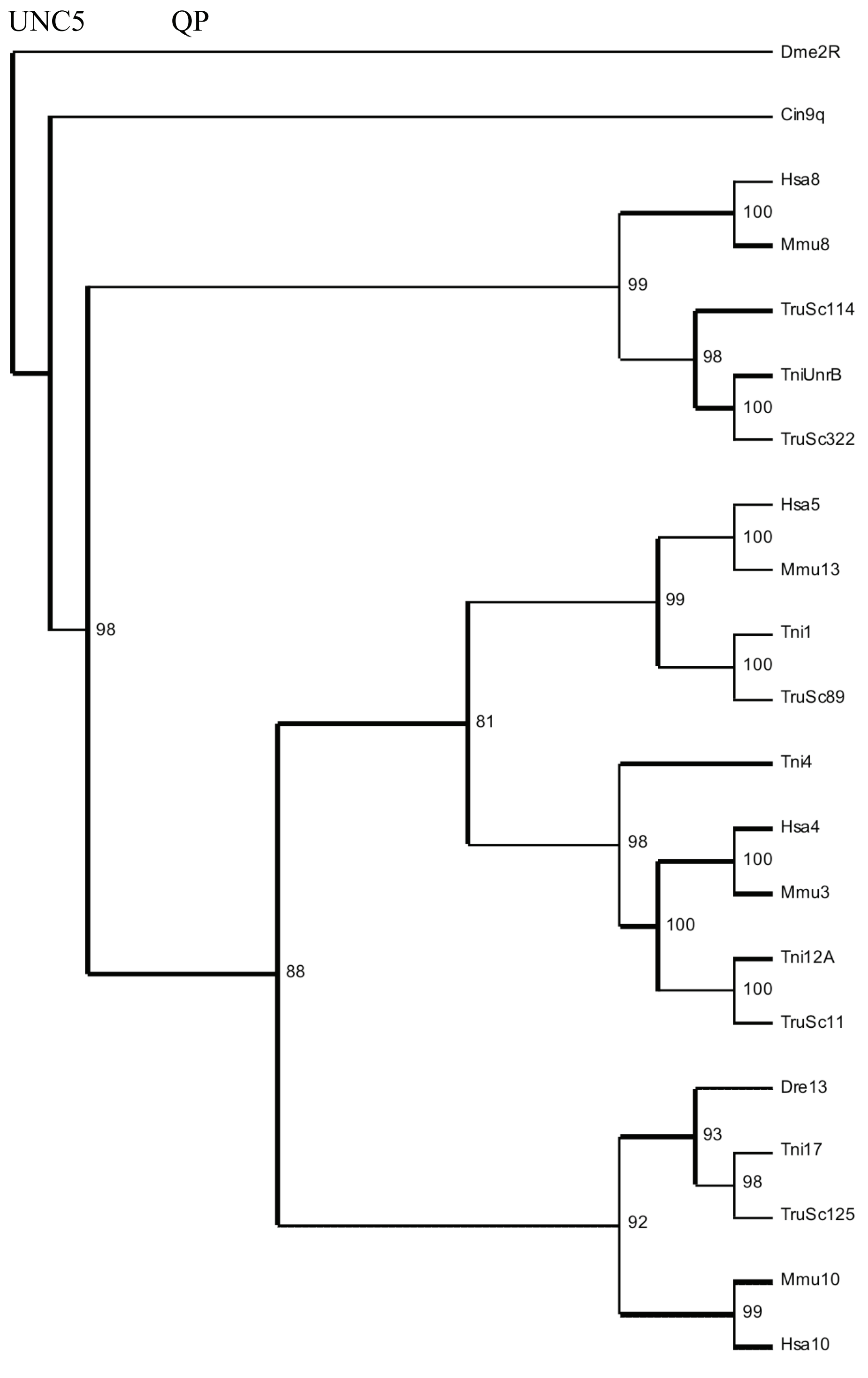
UNC5
QP
Dme2R
Cin9q
Hsa8
100
Mmu8
99
TruSc114
98
TniUnrB
100
TruSc322
98
Hsa5
100
Mmu13
99
Tni1
100
TruSc89
81
Tni4
98
Hsa4
100
Mmu3
100
Tni12A
100
TruSc11
88
Dre13
93
Tni17
98
TruSc125
92
Mmu10
99
Hsa10

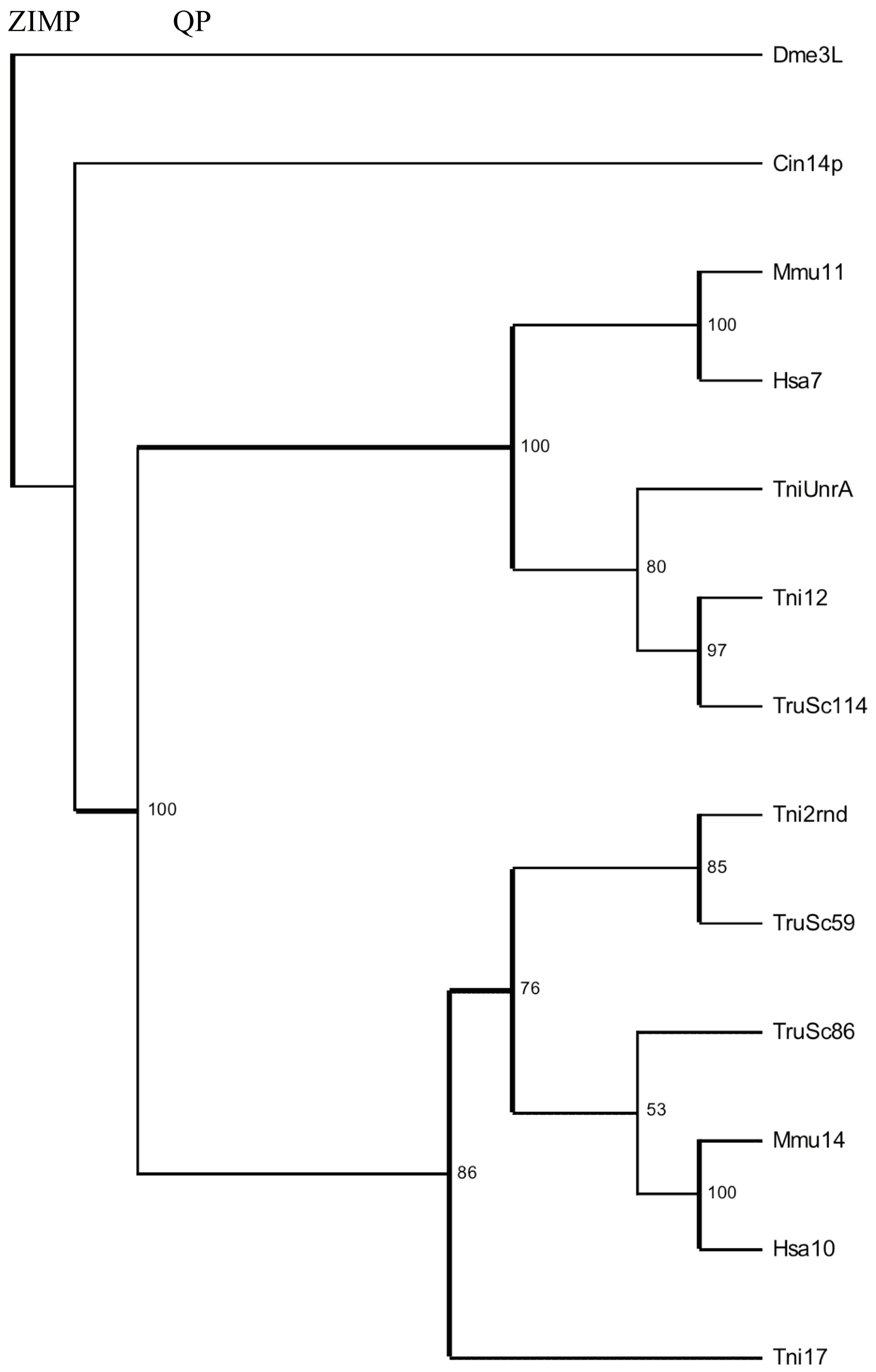
ZIMP
QP
Dme3L
Cin14p
Mmu11
100
Hsa7
100
TniUnrA
80
Tni12
97
TruSc114
100
Tni2rnd
85
TruSc59
76
TruSc86
53
Mmu14
100
Hsa10
86
Tni17

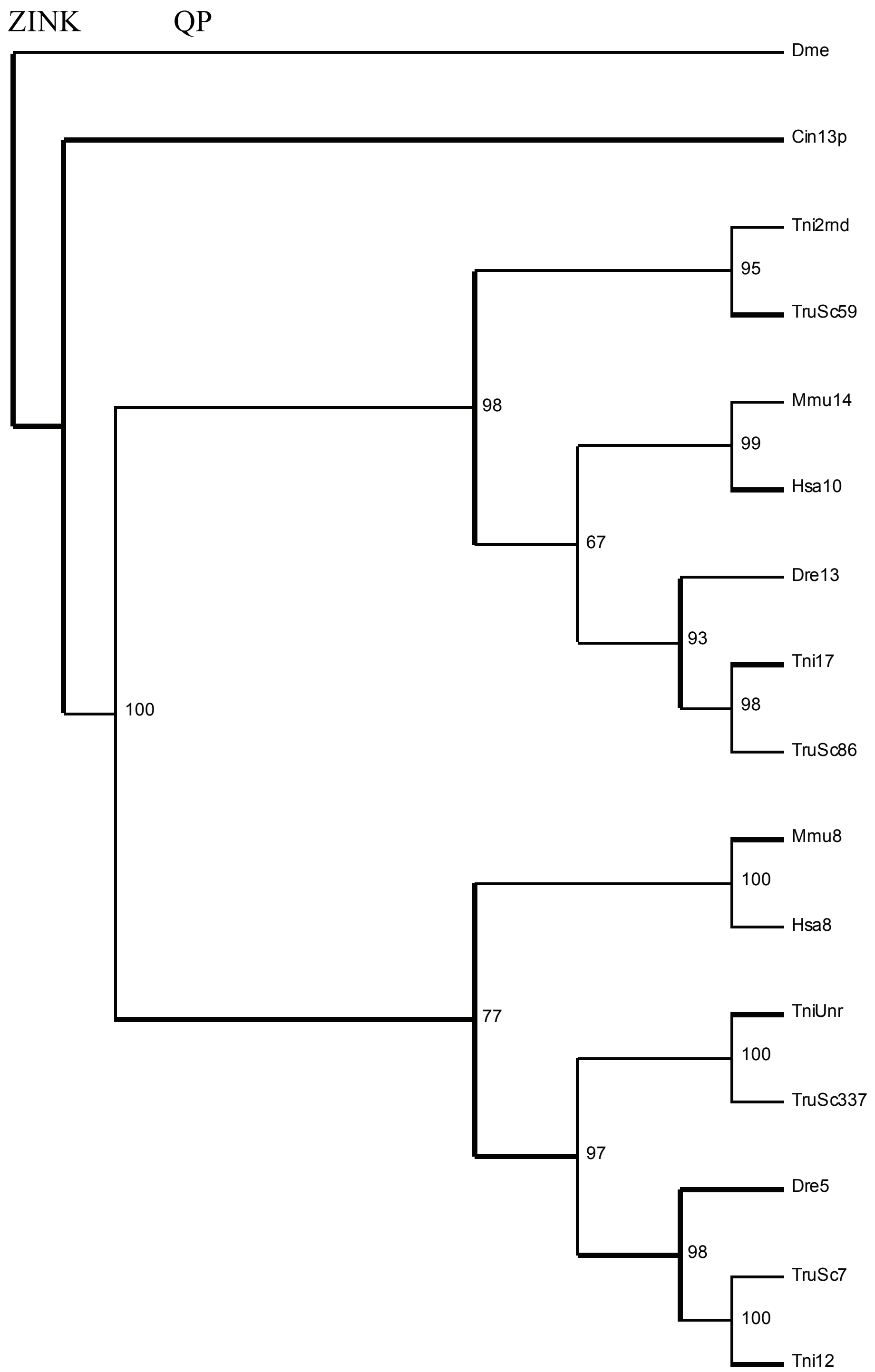
ZINK
QP
Dme
Cin13p
Tni2rnd
95
TruSc59
98
Mmu14
99
Hsa10
67
Dre13
93
Tni17
98
TruSc86
100
Mmu8
100
Hsa8
77
TniUnr
100
TruSc337
97
Dre5
98
TruSc7
100
Tni12
